# Bark and ambrosia beetle fungi emit a greater diversity of volatile organic compounds than other fungi

**DOI:** 10.1093/femsec/fiag115

**Published:** 2026-10-05

**Authors:** Maximilian Lehenberger, Dineshkumar Kandasamy, Pascal Edelmann, Michael Reichelt, Giulio Criscione, Pamela Lehenberger, Almuth Hammerbacher, Jonathan Gershenzon, Peter H W Biedermann

**Affiliations:** Max Planck Institute for Chemical Ecology, Department of Biochemistry, Jena, 07745, Germany; Department of Biology, Lund University, Lund, 22362, Sweden; Department of Terrestrial Ecology, Technical University of Munich, Freising, 85354, Germany; Max Planck Institute for Chemical Ecology, Department of Biochemistry, Jena, 07745, Germany; Department of Agriculture, Food and Environment (Di3A), University of Catania, Catania, 95123, Italy; Leibniz Institute for Natural Product Research and Infection Biology, Hans Knöll Institute, Jena, 07745, Germany; Department of Zoology and Entomology, Forestry and Agricultural Biotechnology Institute, University of Pretoria, Pretoria, 0083,South Africa; Max Planck Institute for Chemical Ecology, Department of Biochemistry, Jena, 07745, Germany; Faculty of Forestry and Wood Sciences, Czech University of Life Sciences Prague, Prague, 16500, Czech Republic; Department of Forest Entomology and Protection, University of Freiburg, Freiburg, 79085, Germany

**Keywords:** mutualistic fungi, fungal volatiles, VOC, aliphatic esters / alcohols, aromatic esters / alcohols, terpenes

## Abstract

Bark and ambrosia beetles are associated with various filamentous fungi, whose roles range from mutualists to antagonists. Mutualists can provide various benefits and are typically selectively dispersed by these wood-boring beetles. However, it is currently unknown how such bark and ambrosia beetles distinguish their fungal mutualists from other fungal associates, including insect pathogens. We hypothesized that volatile organic compounds might help maintain the mutualisms between beetles and specific fungal partners. Here, we compared the volatiles emitted by 30 fungi known to act as mutualists, commensals or antagonists for their beetle hosts. GC-MS analysis showed that all the fungi had characteristic volatile profiles with beneficial fungi emitting a greater diversity of volatiles than other fungal species. The volatile profiles of beneficial fungi were dominated by aliphatic and aromatic alcohols and esters in terms of both diversity and abundance. Furthermore, we demonstrated that the larvae and adults of two ambrosia beetle species can distinguish their mutualistic fungi from others based on volatile cues. Our results highlight the importance of aliphatic and aromatic alcohols and esters as recognition cues for mutualist fungi by wood-boring beetles. Volatiles likely play key roles in maintaining beetle associations with fungi that are essential to their insect host.

## Teaser

Beneficial bark and ambrosia beetle-associated fungi emit distinct volatile profiles rich in aliphatic and aromatic alcohols and esters that may play a key role in maintaining beetle-fungus mutualisms.

## Introduction

Insects that colonize woody tissue encounter challenges due to the low availability of nutrients and the high concentrations of chemical defense compounds (Krokene et al. 2003, Filipiak and Weiner 2014, Filipiak et al. 2016, Six and Elser 2019, Lehenberger et al. 2021). Yet, there are very successful groups of wood-colonizing insects, such as the bark and ambrosia beetles (Coleoptera: Scolytinae), which encompass more than 6000 species worldwide (Kirkendall et al. 2015). Indeed, these groups evolved more than 130–150 Mio years ago and are thus among the oldest groups of wood-feeding insects (Farrell et al. 2001, Mueller et al. 2005, Peris et al. 2021, Ferreira et al. 2026). Bark beetles typically colonize the inner bark (phloem) of trees, while ambrosia beetles drill their galleries deep into the sapwood (Batra 1985, Kirkendall et al. 2015). In recent decades, bark beetle species such as the Eurasian spruce bark beetle *Ips typographus* (Biedermann et al. 2019) and the ambrosia beetle *Xyleborus glabratus* (Rodríguez-Becerra et al. 2024) have received much attention due to their tree-killing capabilities. However, most bark and ambrosia beetles only colonize the wood of weakened or dead trees (Kirkendall et al. 2015).

Both bark and ambrosia beetles are well-known for their rich communities of ectosymbiotic filamentous fungi, yeasts, and bacteria, whose roles range from mutualism to antagonism (Batra 1963, Francke-Grosmann 1967, Hulcr and Stelinski 2017, Banos-Quintana et al. 2024). Mutualistic fungal associates contribute to the success of their insect host, for example, by increasing the nutritional quality of the substrate colonized by beetles (Batra 1967, Francke-Grosmann 1967, Six 2013, Netherer et al. 2016, Wadke et al. 2016, Six and Elser 2019, Lehenberger et al. 2025). In ambrosia beetles, mutualistic fungi are even the sole food source for the beetles and their larvae (Francke-Grosmann 1967). Beside direct nutritional aspects, fungal symbionts may benefit their insect associates by their pathogenicity to the host tree, by reducing the availability of chemical plant defenses (Hammerbacher et al. 2013, Wadke et al. 2016, Lichnock et al. 2026) and/or by repelling antagonistic microbes through releasing antimicrobial chemicals (Ranger et al. 2018, Lehenberger et al. 2021, 2025). In cases where beneficial fungi are obligate, beetle species possess specialized organs, called mycetangia (or mycangia), which maintain the fidelity of beetle-fungus relationships over generations through the transmission of fungal spores by adult offspring when they disperse from their natal nests (Francke-Grosmann 1956, Vazquez-Ortiz et al. 2022). When these dispersing beetles colonize new host-trees, they transmit mutualistic fungi from their mycetangia (Mayers et al. 2022), as well as a variety of hitchhiking commensal or antagonistic fungi (Popa et al. 2025). It has been demonstrated that beetles promote the growth of mutualists over other fungi, either indirectly by choosing substrates that select for their partners (Ranger et al. 2018, Lehenberger et al. 2021) or directly through active management (Diehl et al. 2022). The underlying mechanisms of fungal management remain unknown, as does the ability of beetles to recognize and distinguish between different fungi within their nests.

The mutualistic relationships between animals and their associated microbes are sometimes contingent on the release and perception of volatile organic compounds (Minerdi et al. 2021). A wide variety of volatiles has been identified from fungi (Müller et al. 2013, Hung et al. 2015), including from a few species of bark and ambrosia beetle mutualists (Kandasamy et al. 2016, 2019, Gugliuzzo et al. 2023, Kandasamy et al. 2023). These species were found to have volatile profiles rich in terpenoids and a set of aliphatic and aromatic compounds, which typically have a very pleasant fruity odor for humans (Kandasamy et al. 2019) and were first noticed more than 180 years ago when ambrosia beetles were originally described (Schmidberger 1836). The mutualistic fungi of *I. typographus*, for example, emit substantial amounts of isoamyl alcohol and 2-phenylethanol as well as their respective acetates (Kandasamy et al. 2019). Similar volatiles were measured in one of the ambrosia beetle fungi, *Ambrosiella grosmanniae* (Gugliuzzo et al. 2023) suggesting that such volatiles may be more generally emitted by mutualistic fungi.

The volatiles of mutualistic fungi have been demonstrated to attract their beetle hosts in several studies (Hulcr et al. 2011, Kandasamy et al. 2016, Ranger et al. 2021, Gugliuzzo et al. 2023). For instance, the ambrosia beetle *Xylosandrus germanus* was found to be attracted to volatiles of its mutualistic fungus *Ambrosiella grosmanniae* but not to antagonistic fungi such as *Beauveria bassiana* and *Trichoderma harzianum*. Moreover, the bark beetle *I. typograpus* as well as the ambrosia beetle *Trypodendron lineatum* were found to have specialized olfactory sensory neurons that detect fungus-derived compounds (Kandasamy et al. 2019, Biswas et al. 2023). The attractiveness of fungal volatiles to larvae, rather than adults, has been less studied (Luna et al. 2014), though larvae of ambrosia beetles are obligately dependent on their fungal mutualists for nutrition (Batra 1963, Francke-Grosmann 1967). It is also not clear if bark and ambrosia beetles can distinguish between their mutualistic fungi and related mutualists of other beetles, which may emit similar compounds.

Independent of their primary role, fungi provide beetles with nitrogen and other nutrients (Lehenberger et al. 2025) that they may even translocate from some distance (Six and Elser 2019, Lehenberger et al. 2021). In mutualisms between plants and animals, nutritional quality is often signaled by olfactory cues (Nevo et al. 2018, Ito et al. 2021). For example, plants signal the nutritional value of fruit by releasing volatiles, such as esters, to attract dispersal agents (Nevo et al. 2022). However, the volatile compounds mediating attractiveness to fungal mutualists remain poorly understood (Elliott et al. 2022). In fruits, many esters responsible for attractive aromas can function as honest indicators of nutritional quality (Nevo et al. 2022). Here, the biosynthesis of these volatiles relies on amino acid metabolism (Baldwin 2010, Maoz et al. 2022). Similarly, in fungi volatile aliphatic and aromatic alcohols and esters responsible for aromas are derived from amino acids, such as phenylalanine and leucine via the Ehrlich pathway (Rumpl et al. 2025). The abundance of free amino acids in fungal biomass, which affects the nutritional value of fungi, may be reflected in the production of volatile cues perceived by beetles.

Here, we hypothesized that the emission of aliphatic alcohols and esters is more common in mutualist fungi of bark and ambrosia beetles than in associated, but non-mutualist fungi. To test this hypothesis, we collected the headspace volatiles from 30 different fungi, both bark and ambrosia beetle mutualists and non-mutualistic fungi. We also analyzed the levels of free amino acids in the fungal biomass to determine if this is correlated with volatile emission. Finally, we performed behavioral assays with adults and larvae of the two ambrosia beetle species *Xyleborinus saxesenii* (Ratzeburg) and *Xylosandrus germanus* (Blandford) to test if these insects are attracted to their respective mutualistic fungi and can distinguish them from related mutualists of other ambrosia beetle species.

## Materials and methods

### Fungal isolates and culture conditions

We examined 25 common fungal associates of several widespread bark and ambrosia beetle species drawn from three ascomycete orders: Ophiostomatales (17 × Ophiostomataceae), Microascales (4 × Ceratocystidaceae, 1 × Microascaceae), and Hypocreales (2 × Nectriaceae), as well as from one Saccharomycete order: Saccharomycetales (1 × Alloascoideaceae). Further, we included four common fungal pathogens from the three ascomycete orders Hypocreales (1 × Cordycipitaceae), Sordariales (1 × Chaetomiaceae) and Eurotiales (2 × Aspergillaceae) as well as the nematophagous fungus *Esteya vermicola* from the Ophiostomatales (Ophiostomataceae), which can be also isolated from bark beetles (Kubátová et al. 2000). For an overview of all strains including additional information see Suppl. Table S1 and S2. All fungal isolates were stored at −80°C in glycerol (80%) and glycerol / peptone (80% / 1%, Roth; Germany) at the Max-Planck Institute for Chemical Ecology (MPI-CE, Jena, Germany). Prior to the experiment, all fungi were revived on potato dextrose agar (PDA; Sigma Aldrich, Germany) serving as our experimental culture medium, and maintained at 4°C until they were subcultured in fresh PDA at room temperature (RT) for 4-6 days before experimental use.

All fungi in this study were classified into groups based on their ecology, (Suppl. Table S1): (**a**) ambrosia beetle mutualists (named “ambrosia beetle fungi”; N = 10), (**b**) bark beetle mutualists (named “bark beetle fungi”; N = 11), and (**c**) beetle-associated fungi with antagonistic or commensal roles (named “other fungi” or “others”; N = 9). The latter group comprises a broad set of fungi not specifically associated with bark and ambrosia beetles, but that beetles may naturally encounter. These species have been described as saprophytes, plant pathogens, insect pathogens or as species with no known role.

### DNA extraction and LSU / ITS / βT barcoding

Fungi were identified via Sanger sequencing and sequences obtained were uploaded to the NCBI GenBank database. Accession numbers were added to Suppl. Table S3. See SI Methods for further details.

### Maximum likelihood analyses

To give an overview of the taxonomic and evolutionary relationships of the fungi studied, we performed maximum likelihood analyses of their large subunit rRNA (LSU, 28S) sequences and those of related species using the software R (version 4.5.1) and IQ-Tree (Version 2.2.2.). Overall, our phylogeny comprised 56 different fungal species of which 24 sequences were generated in this study while the remaining 32 sequences were obtained from the National Center for Biotechnology Information (NCBI GenBank database, see Suppl. Tables S2 and S3). The final tree was modified using the software Adobe Illustrator (version CS5). See SI Methods for further details.

### Analyses of volatile organic compounds (VOCs) from fungal cultures

The headspace volatiles from each of the 30 individual filamentous fungi studied were collected at two different time intervals, 6 and 12 days after inoculation on PDA medium. We embedded a sterile metal wire equipped with 2–3 polydimethylsiloxane (PDMS) tubes (Kallenbach et al. 2014) (∼5 mm long) per plate in the fungus-colonized PDA (performed on a UV-sterilized bench using oven-sterilized metal forceps). All plates were sealed with parafilm and headspace VOCs were collected for 30 min. Afterwards, each PDMS tube was carefully removed from the metal wires and transferred to a 1.5 ml brown glass vial and stored at −20°C until further processing [see also ref. (Kandasamy et al. 2023)]. For each species, 4–5 replicates were made with a sterile PDA culture as a control for both examined time points. VOCs identified from our control samples were excluded from the analyses.

Samples were analyzed on a GC-2010 plus gas chromatograph, which was coupled to a MS-QP2010 quadrupole mass spectrometer with a TD-20 thermal desorption unit (Shimadzu, Japan) and a GC Cryo-Trap (Tenax®). Analytes were injected onto a Rtx-5MS GC column using split mode [1 : 100]. Injector and detector settings, column conditions, and temperature program followed the method described in ref. (Kandasamy et al. 2019). Compounds were identified by comparing their mass spectra and retention times to those of authentic standards and to spectra in the Wiley / NIST reference library.

All VOCs were classified into nine chemical groups: aliphatic esters, aromatic esters, aliphatic alcohols, aromatic alcohols, aliphatic ketones, aromatics, monoterpenes, sesquiterpenes, and others (see also Suppl. Fig. S2-3 as well as Suppl. Table S6). Further, an additional dataset was generated containing the absolute number of VOCs that were emitted by each fungus within each chemical group (see also Suppl. Data Fig. 2).

To estimate fungal growth, images of all sampled plates were taken (Sony alpha 5000, 12 × in macro mode) after volatile collection at each time interval (for all replicates) and analyzed for fungal surface area using ImageJ software (version 1.52a). We divided the amount of each VOC collected (total ion counts) by the calculated surface area (mm^2^). See SI Methods for details on the statistical analyses.

### Quantitative analysis of fungal free amino acids over culture development

We analyzed the free amino acid composition of fungal biomass (N = 30, 4–5 replicates per fungus, see Suppl. Table S4) harvested at two different time points (6 and 12 days after culture inoculation). Fungi were cultured on PDA overlain with a disk of sterilized cellophane (6 × 6 cm, NeoLab, Germany), which was added to each petri dish immediately after the media had cooled down. Afterwards, we inoculated a plug of actively growing fungal mycelium (Ø 3 mm) on the center of each plate and incubated it tightly sealed with parafilm at RT. At the two examined time intervals (6 and 12 days), we removed pure fungal mycelium from each plate, and freeze-dried it to calculate dry weight. For the methanolic extractions, we added 300 µl of methanol (ultrapure, for liquid chromatography; Merck, Germany) and then homogenized each sample using a bead beater (SKANDEX S-7) for 10 min at moderate intensity. Afterwards, samples were centrifuged (10 000 r/m for 5 min) and the supernatant was removed for subsequent analyses.

We quantified the following 18 free amino acids for all examined fungi: alanine (Ala), serine (Ser), proline (Pro), valine (Val), threonine (Thr), isoleucine (Ile), leucine (Leu), aspartic acid (Asp), glutamic acid (Glu), methionine (Met), histidine (His), phenylalanine (Phe), arginine (Arg), tyrosine (Tyr), tryptophan, (Trp), asparagine (Asn), glutamine (Gln), and lysine (Lys). Quantification was performed by LC-MS/MS on a Zorbax Eclipse XDB-C18 column (50 mm × 4.6 mm, 1.8 μm, Agilent Technologies, Germany) using a TripleQuad-MS system (Sciex QTRAP6500), after diluting the extract (1 : 50) with water containing 10 µg ml^−1^ of a mix of ^15^ N/^13^C labelled amino acids (Isotec, Miamisburg, OH, USA) [for details on method see (Crocoll et al. 2016)]. We quantified amino acids (in nmol/mg) relative to the peak area of their corresponding labelled amino acids, except for tryptophan (relative to phenylalanine, response factor 0.42). Remaining culture supernatant was stored at −20°C.

Statistical analyses and visualizations via heatmaps and boxplots were performed as described above for the identification of headspace volatiles using global and pairwise PERMANOVAs, fitted GLMs as well as t-tests or Wilcoxon rank-sum tests. All plots were modified using the software Adobe Illustrator (CS5).

### Volatile attraction assay

To examine a potential attractive effect of mutualistic fungal associates or pathogens on two widespread ambrosia beetle species [*Xyleborinus saxesenii* (Ratzeburg) & *Xylosandrus germanus* (Blandford)], we utilized separate experimental setups for adult beetles and for their larvae. We compared the attractiveness of the associated mutualistic fungus to that of a closely related mutualist, a distantly-related mutualist, and two fungal pathogens.

Specifically, the experimental setup for adult beetles followed the approach described by (Kandasamy et al. 2019). Here, we used standard Petri dishes, in which we embedded three Eppendorf Tubes (1.5 ml, closed lid) into the bottom. Tubes were equidistant from each other and arranged in a triangle. Two tubes contained one hole (4 mm Ø) on the upper front side, which allowed beetles to enter the tube, but prevented them from leaving it. The third tube was missing holes and thus could not be entered by beetles, but was added to stabilize our experimental setup. Further, we drilled six small holes equally distributed on the outer walls of the petri dish to create air flow and ensure an odor gradient. The lid of the petri dish was closed tightly with parafilm, so that the beetles could not escape from the Petri dish. We roughed up the inner surface of the petri dish to prevent the beetles from slipping. One plug (8 mm Ø) of PDA, which was covered with a 4-day old culture of actively growing fungus, was added to one tube within our experimental setup. As a control, we used a plug of the same size with PDA and no fungus. One female beetle was placed on each plate and was tested only once. We transferred all plates (closed lid; N = 20 per tested fungus, randomized to exclude side-specific effects) to a laminar hood in darkness at 25°C and monitored choices (active decision for either control or PDA-Fungus plug) every 2 h for up to 6 h.

For investigating larvae, we followed the setup described by (Luna et al. 2014). Fungi for testing were pre-cultured on 4% PDA for 4 days at 25°C. Afterwards, we added a plug (8 mm Ø) of actively growing mycelium to one edge of our experimental medium (0.3% peptone, 0.3% glucose, 1.5% agar). Fungi were allowed to colonize the medium for 3 days. A 4% PDA plug of the same size was added to the opposite edge prior to the experiment and served as our control. We transferred one larva to the middle of the petri dish and incubated all plates (closed lid; N = 20 per tested fungus, randomized to exclude side-specific effects) in a laminar hood in darkness at 25°C. Each larva was only tested once. Larval choices (whether burrowing into the control medium or fungus-colonized medium) were monitored periodically for up to 12 h.

Beetles and larvae used for the experiment were laboratory reared using an artificial sawdust-based medium [see Supp. Table S5 and (Biedermann et al. 2013)]. For the experiments, we exclusively used adult female beetles that had naturally dispersed from independent colonies and were collected immediately after dispersal, thereby ensuring that the tested adults were of similar age and developmental stage. The dispersing beetles were removed from the colonies the day before starting the experiment and incubated overnight on moist tissue at 25°C. Larvae were removed from independent galleries 6–8 h before the experiment by actively opening individual nests and were stored at 25°C on moist tissue paper. Statistical differences were determined in R (version 4.5.1) using Fisher’s exact test (fungus choice versus control choice) including Bonferroni correction to account for multiple testing. The final plots were modified using the software Adobe Illustrator (CS5).

## Results

### Mutualists of bark and ambrosia beetles emit a greater diversity of volatile compounds than non-mutualistic fungi

We compared the volatile profiles of 30 different fungi, of which 25 species are common associates of bark and ambrosia beetles distributed over several orders of the Ascomycota (see Fig. 1 and Suppl. Table S1). A phylogeny based on gene sequences encoding the large subunit (28S) of rRNA is presented in Fig. 1, which is congruent with those previously published (Rollins et al. 2001, Alamouti et al. 2009, Dreaden et al. 2014, Vanderpool et al. 2018).

**Figure 1:**
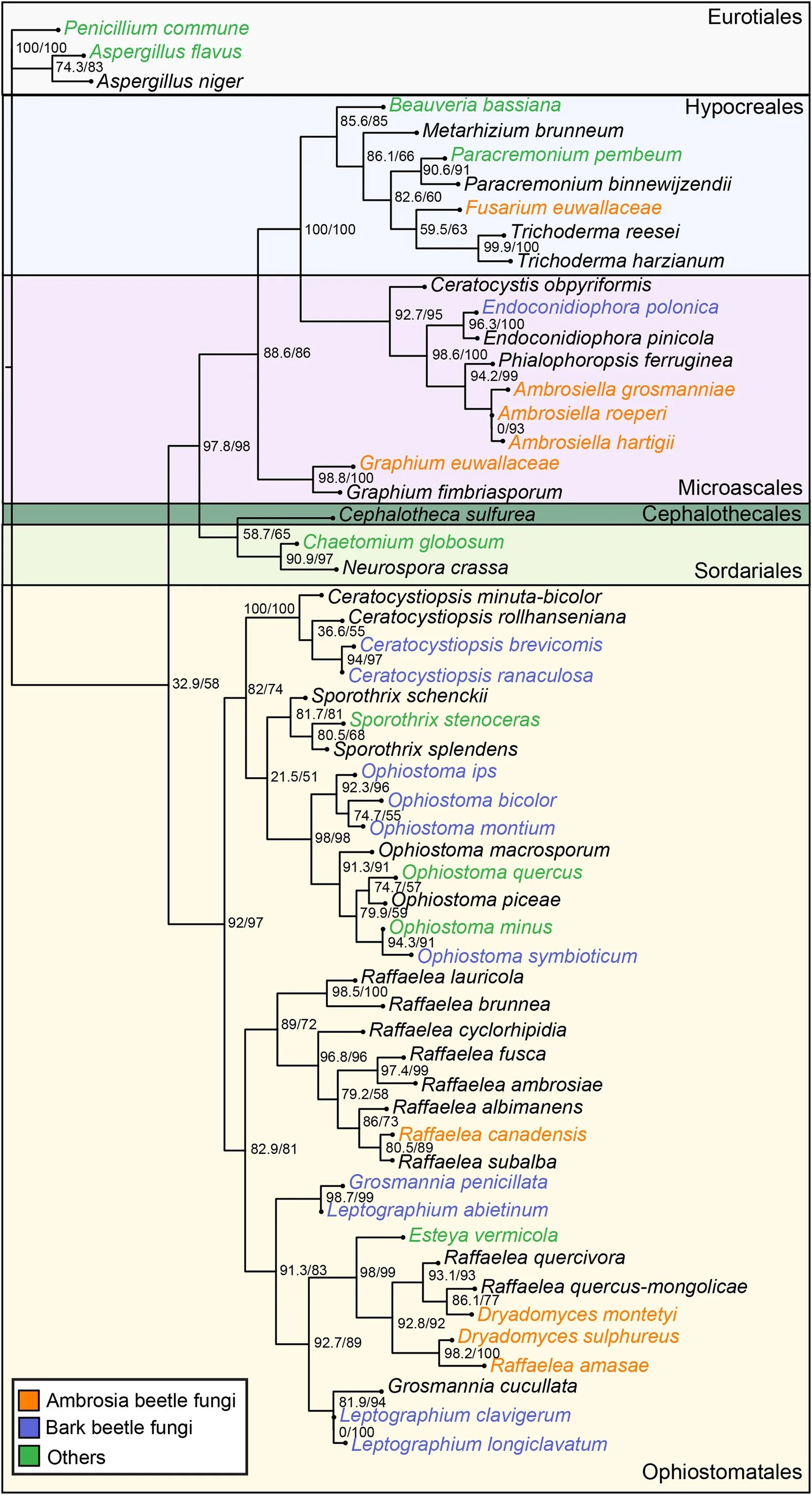
Maximum likelihood phylogeny of all fungi investigated in this study (except *Alloascoidea hylecoeti* in the Saccharomycetales) based on fungal LSU sequences. All studied fungi are highlighted in orange, blue, or green depending on their classification as ambrosia beetle fungi, bark beetle fungi or others, respectively. Shimodaira–Hasegawa approximate likelihood ratio test (SH-aLRT) support values (left number) and ultrafast bootstrap support values (right number) are given for phylogenetic replacement.

A global PERMANOVA revealed a significant difference in volatile composition among fungi classified as ambrosia beetle mutualists, bark beetle mutualists, or “other associates” (PERMANOVA: *F* = 3.38, *R*^2^ = 0.2, *P* = 0.001; Fig. 2a; Suppl. Table S6). Pairwise comparisons indicated that bark and ambrosia beetle mutualists emitted a significantly higher diversity of volatiles in comparison to fungi classified as others (*P* < 0.028, pairwise PERMANOVAs; Fig. 2a), but did not differ from each other. This finding was confirmed by the overall sum of emitted volatile compounds, where beetle mutualists emitted a larger total number of compounds than other fungi (*P* < 0.003, fitted GLMs with Tukey post-hoc test, Fig. 2b). Among compound classes, aliphatic and aromatic esters and alcohols were more abundant in the volatiles of mutualists than others (*P* < 0.0021), while ambrosia beetle mutualists did not differ from bark beetle mutualists. Ambrosia beetle mutualists emitted a significantly higher number of aliphatic and aromatic esters compared to other fungi (*P* < 0.0013; fitted GLMs with Tukey post-hoc test; Fig. 2c) without differing from bark beetle mutualists. Among the aliphatic and aromatic alcohols, bark beetle mutualists emitted a higher number of compounds than other fungi (*P* < 0.018), while there was no difference compared to ambrosia beetle mutualists. The majority of other fungi did not emit aromatic alcohols and none of them emitted aromatic esters. Taken together, these results show that bark and ambrosia beetle mutualists generally emit a higher number of volatile compounds compared to other fungi, with aliphatic and aromatic alcohols and esters being especially responsible for this difference.

**Figure 2:**
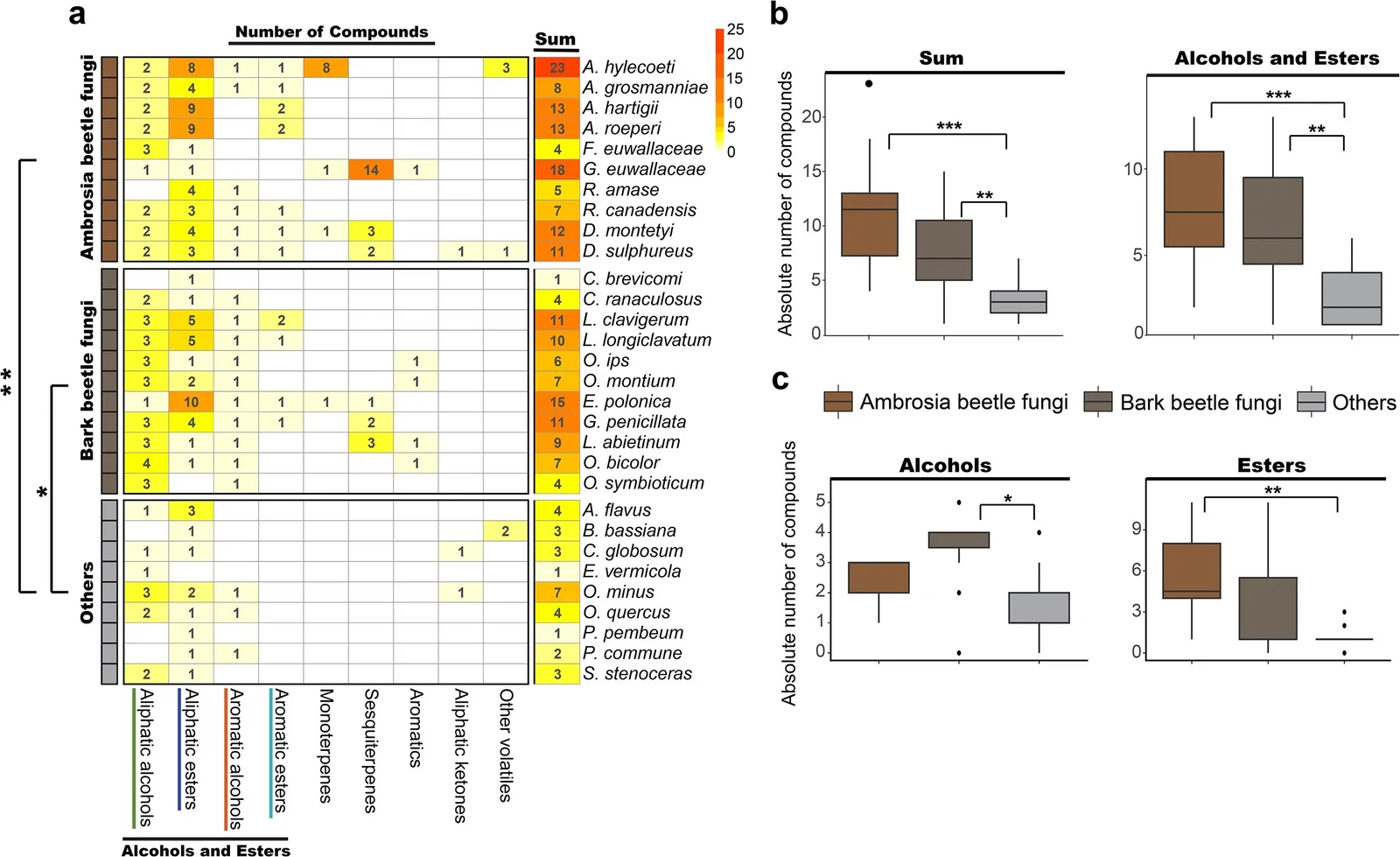
Ambrosia and bark beetle mutualists emit higher numbers of volatile compounds than other fungi, especially aliphatic and aromatic alcohols and their esters. In total, 81 volatile compounds were identified from fungi cultured on 6d old potato dextrose agar plates and were classified into nine different chemical groups. (a) Heatmap showing the total number of compounds identified from the volatile profiles of 30 tested fungi. Numbers in each cell represent the total number of volatile compounds produced by an individual fungus from each chemical group; empty cells indicate the absence of volatiles in this specific chemical group. Both ambrosia and bark beetle mutualists differed in the number of compounds emitted compared to other fungi. (Pairwise PERMANOVAs: ns—*P* > 0.05, *—*P* < 0.05, **—*P* < 0.01, ***—*P* < 0.001). (b, left) Number of volatiles representing all chemical groups from ambrosia beetle mutualists bark beetle mutualists and other fungi. Ambrosia and bark beetle mutualists emitted a larger number of volatiles compared to other fungi. (b, right) Number of volatiles representing only the four most abundant groups (aliphatic and aromatic alcohols and esters) from ambrosia beetle mutualists, bark beetle mutualists and other fungi. Ambrosia and bark beetle mutualists emitted a larger number of volatiles compared to other fungi. (c, left) Number of aliphatic and aromatic alcohols identified from ambrosia beetle mutualists, bark beetle mutualists and other fungi. Bark beetle mutualists released a higher number of such alcohols compared to other fungi, while no difference was detected compared to ambrosia beetle mutualists. (c, right) Number of aliphatic and aromatic esters identified from ambrosia beetle mutualists, bark beetle mutualists and other fungi. Ambrosia beetle mutualists released a higher number of such esters compared to other fungi, while no difference was detected towards bark beetle mutualists. Significant differences between groups are denoted by asterisks (ns—*P* > 0.05, *—*P* < 0.05, **—*P* < 0.01, ***—*P* < 0.001; fitted GLMs with Tukey post-hoc test).

### Fungal mutualists emit high amounts of certain aliphatic and aromatic alcohols and esters with species-specific differences

Comparing individual compounds, fungal mutualists emitted significantly higher amounts of major volatile compounds than other fungi. Bark beetle mutualists, in particular, emitted significantly higher amounts of ethanol and isobutyl alcohol, two aliphatic alcohols, compared to ambrosia beetle mutualists and other fungi (*P* < 0.035; fitted GLMs with adjusted pairwise contrasts or Fisher´s exact tests with Holm-adjusted pairwise comparisons; see Suppl. Table S6 for further details; Fig. 3a). The content of isoamyl alcohol was similar between bark and ambrosia beetle mutualists, but bark beetle mutualists had higher values than others (*P* < 0.031). Further, bark beetle mutualists emitted more of the aromatic alcohol 2-phenylethanol than other fungi (*P* < 0.014, Fig. 3b) without differing from ambrosia beetle mutualists. Aliphatic esters, such as ethyl acetate and isoamyl acetate, were especially prevalent in ambrosia beetle mutualists; these esters were emitted in significantly higher amounts than in other fungi (P < 0.038; Fig. 3c). Phenethyl acetate, an aromatic ester, was found to be abundant in a few of the mutualistic fungi but was completely absent in non-mutualistic fungi (Fig. 3d).

**Figure 3:**
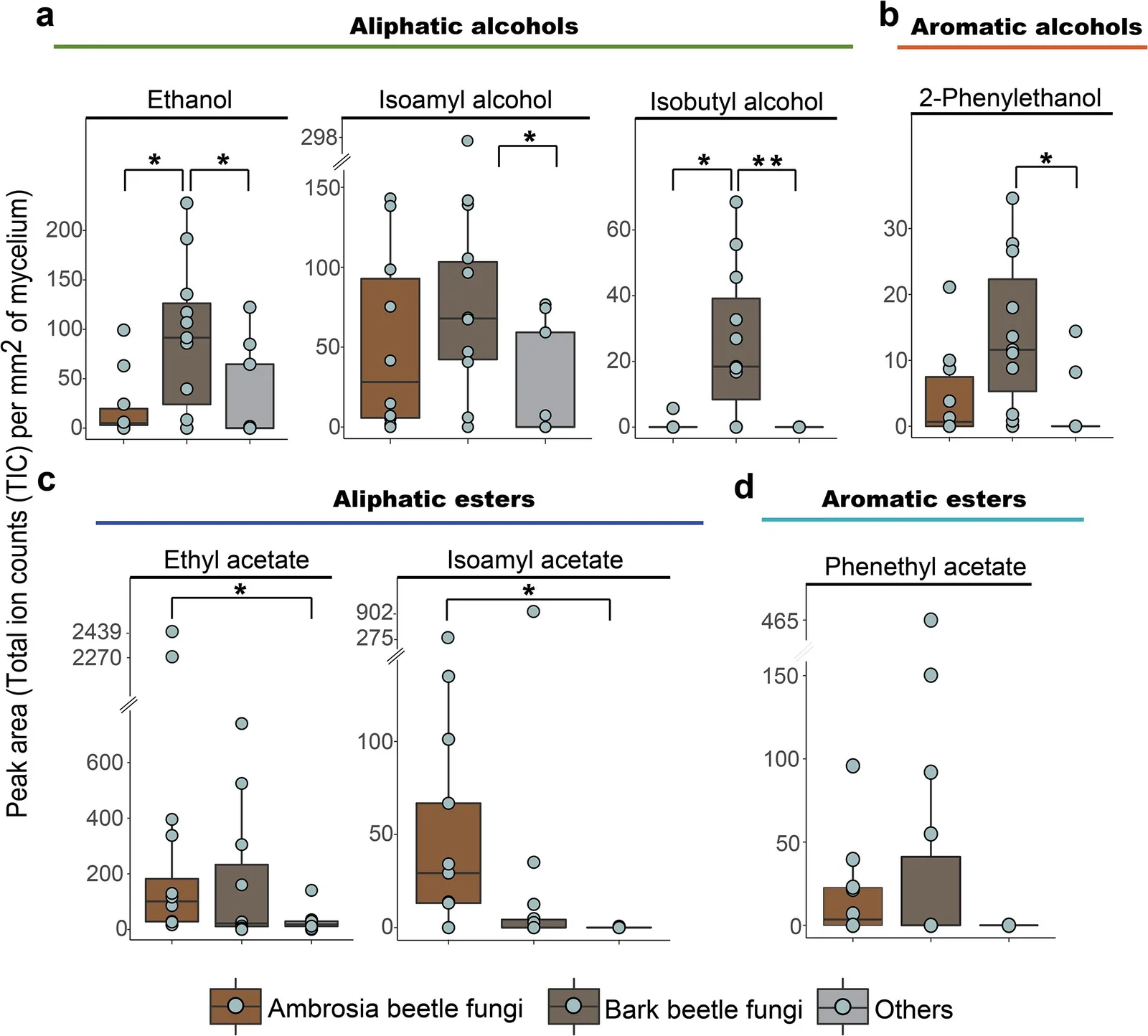
Bark and ambrosia beetle mutualists emit high amounts of specific aliphatic and aromatic alcohols and their esters (a) Emission of the major aliphatic alcohols, ethanol and isobutyl alcohol, were significantly higher from bark beetle mutualists compared to ambrosia beetle mutualists and other fungi, while isoamyl alcohol was more abundant in bark beetle mutualists compared to other fungi without differing from ambrosia beetle mutualists (b) Emission of the most abundant aromatic alcohol, 2-phenylethanol, was significantly higher for bark beetle mutualists compared to other fungi. (c) Emission of the two major aliphatic esters, ethyl acetate and isoamyl acetate, were both significantly higher from ambrosia beetle mutualists compared to other fungi. (d) Emission of phenethyl acetate, the major aromatic ester in bark and ambrosia beetle mutualists, was absent in other fungi. Each circle in boxplots indicates the mean number of volatiles collected from an individual fungus. Significant differences between groups are denoted by asterisks (ns—*P* > 0.05, *—*P* < 0.05, **—*P* < 0.01, ***—*P* < 0.001; fitted GLMs with adjusted pairwise contrasts or Fisher´s exact tests with Holm-adjusted pairwise comparisons).

The individual fungi within each group differed widely in the emission of specific compounds. For instance, ethanol was most abundant in *Raffaelea canadensis* and *Dryadomyces montetyi* within the ambrosia beetle fungi (*P* < 0.0029, fitted GLMs with adjusted pairwise contrasts; Fig. 4a), while *Grosmannia penicillata* was among the strongest bark beetle fungus emitters of this compound (*P* < 0.001). Non-mutualistic fungi mostly did not emit ethanol (five out of nine fungi), though *Ophiostoma quercus* and *Sporothrix stenoceras* emitted the highest amounts in this group (*P* < 0.01). Isoamyl alcohol was most abundant in *R. canadensis, D. montetyi, Alloascoidea hylecoeti*, and *D. sulphureus* (*P* < 0.026) for ambrosia beetle fungi, while *G. penicillata* was the strongest emitter within the bark beetle fungi (*P* < 0.001). Most other fungi did not emit isoamyl alcohol (five out of nine), though again *S. stenoceras, O. quercus*, and *O. minus* were strong emitters (*P* < 0.001). The aliphatic ester ethyl acetate was generally among the most abundant volatile compound. Here, *Ambrosiella roeperi* and *Ambrosiella hartigii* were the strongest emitters within ambrosia beetle fungi (*P* < 0.034, Fig. 4b), while *Endoconidiophora polonica* and *Leptographium clavigerum* emitted the highest amounts within the bark beetle fungi (*P* < 0.0059). Other fungi typically emitted lower amounts, with the exception of S*. stenoceras* (*P* < 0.0038). Further profiles of individual volatile compounds can be found in the supplementary material (see Suppl. Fig. S1).

**Figure 4:**
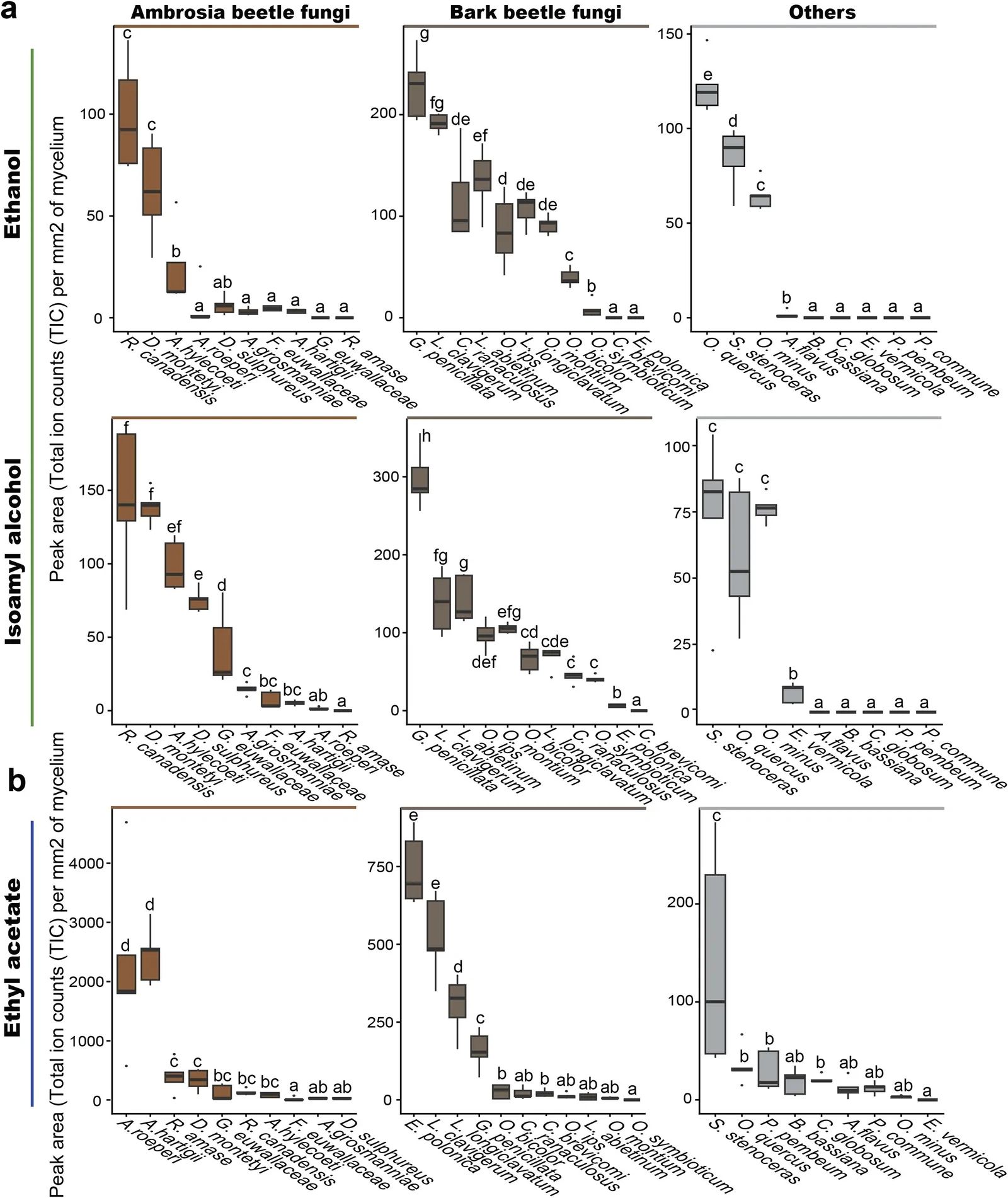
Individual species of bark and ambrosia beetle mutualists differ in their emission of major volatile compounds. Emission of the most abundant (a) aliphatic alcohols ethanol and isoamyl alcohol, and (b) the aliphatic ester ethyl acetate among the individual fungal species of each group (N = 4–5 for each fungus) grown on potato dextrose agar for 6 d. Most bark and ambrosia beetle mutualists emitted higher amounts of these volatile compounds compared to other fungi. The latter group contained multiple species from which such volatiles could not be detected. Significant differences within each individual group are denoted by letters above each boxplot (ns—*P* > 0.05, *—*P* < 0.05, **—*P* < 0.01, ***—*P* < 0.001; fitted GLMs with adjusted pairwise contrasts).

Overall, mutualists were stronger emitters of the major volatiles than other non-mutualistic fungi. The compounds involved were aliphatic and aromatic alcohols and esters, such as 2-phenylethanol, ethanol, isoamyl alcohol, isobutyl alcohol, ethyl acetate, and isoamyl acetate.

### Bark beetle mutualists accumulate higher amounts of free amino acids in their tissues than non-mutualistic fungi

A global PERMANOVA revealed a significant difference in the free amino acid profiles of our three fungal groups (PERMANOVA: *F* = 3.98, *R^2^* = 0.2, *P* = 0.007). Bark beetle mutualists differed significantly from the two other groups (*P* < 0.013; pairwise PERMANOVAs; Suppl. Fig. S4; see Suppl. Table S6 for further details). They accumulated significantly more amino acids than the other two groups after 6 d of incubation (*P* < 0.023; fitted GLMs with adjusted pairwise contrasts; Suppl. Fig. S5a), and also more of the amino acids that are abundant in woody tissue (Dietrichs 1969, Scurfield and Nicholls 1970, Gallet-Budynek et al. 2009) (*P* < 0.0042; Suppl. Fig. S5b). Bark beetle mutualists also accumulated higher amounts of essential and non-essential amino acids compared to other fungi. Significantly higher levels were found for the essential histidine, arginine and threonine (Fig. 5a) and the non-essential alanine, asparagine, glutamine, and serine (*P* < 0.034, Fig. 5b). On the other hand, the overall content of essential amino acids did not differ between the mutualists of bark beetles and those of ambrosia beetles. Ambrosia beetle mutualists accumulated significantly less non-essential amino acids than bark beetle mutualists (*P* < 0.0055), but did not differ from other fungi. Significantly lower levels were found for asparagine, glutamic acid, and glutamine (*P* < 0.019) compared to bark beetle mutualists.

**Figure 5:**
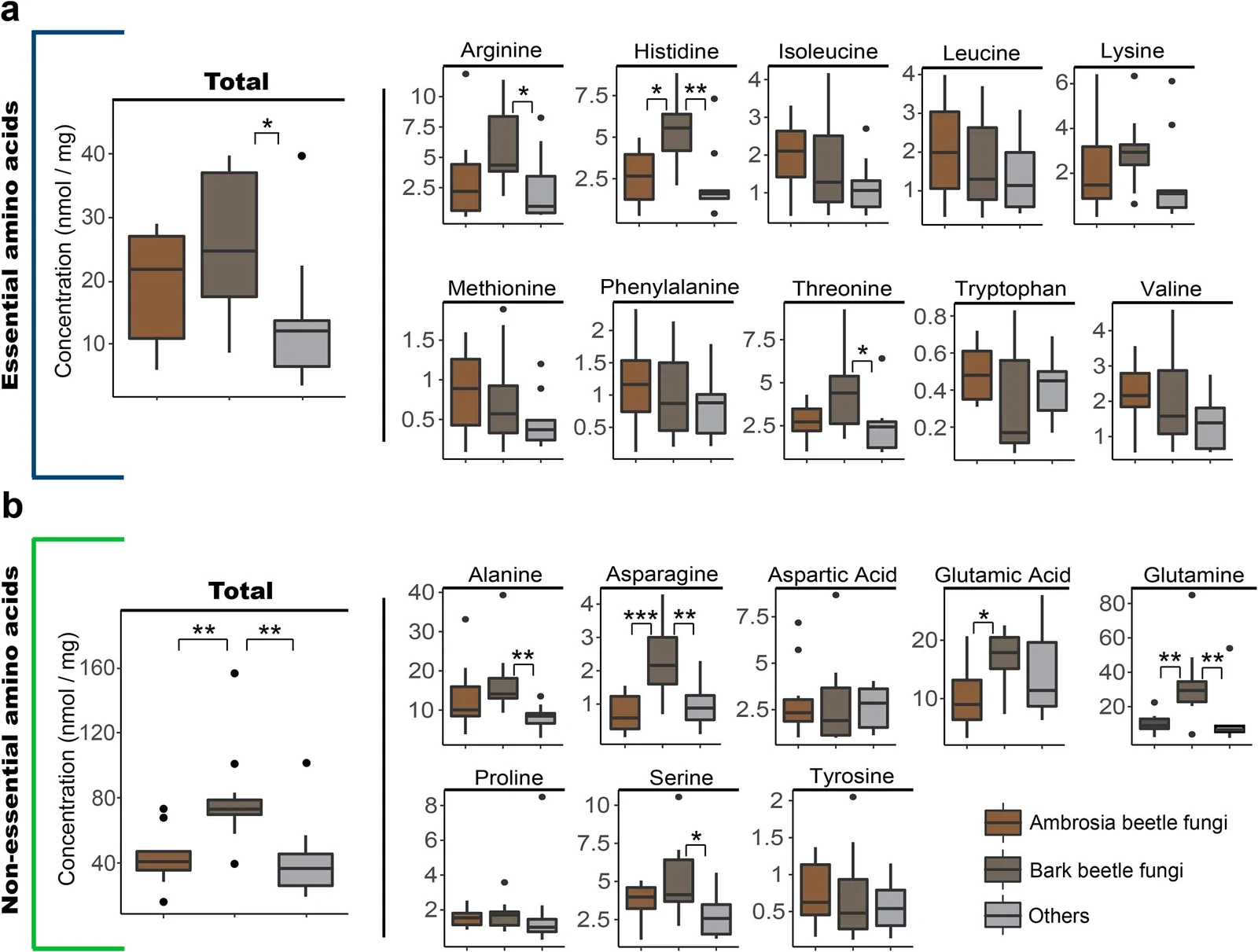
Bark beetle mutualists accumulate higher quantities of many essential and non-essential amino acids than the other groups. Concentration of free amino acids from 6 d old PDA cultures. (a) Bark beetle mutualists accumulated higher concentrations of total essential amino acids than other fungi, with arginine, histidine, and threonine driving this trend. (b) Bark beetle mutualists accumulated higher concentrations of total non-essential amino acids compared to ambrosia beetle mutualists and other fungi, with alanine, asparagine, glutamic acid, glutamine, and serine driving this trend. Asterisks indicate significant differences between groups (ns—*P* > 0.05, *—*P* < 0.05, **—*P* < 0.01, ***—*P* < 0.001; fitted GLMs with adjusted pairwise contrasts).

Due to the frequent utilization by yeast and filamentous fungi of the Ehrlich pathway to convert amino acids to their corresponding alcohols and acetate esters (Rumpl et al. 2025), we anticipated that phenylalanine (Phe) and leucine (Leu) might be correlated as precursors with the quantity of two of the major alcohols emitted from our cultures, 2-phenylethanol and isoamyl acetate, respectively. Comparing the two time points, however, there were no differences in total free amino acids, nor in Phe and Leu for all three groups, even though the content of Phe and Leu showed a tendency to decrease with development (*P* > 0.075; Wilcoxon’s test or t-test; see Suppl. Fig. S6 a-b). The emission of the aliphatic alcohols, ethanol and isoamyl alcohol, was significantly reduced after 12 d for bark beetle mutualists (*P* < 0.02; Wilcoxon’s test or t-test; see Suppl. Fig. S6 c-d), but not for ambrosia beetle mutualists (*P* > 0.07) nor for other fungi. On the other hand, the aliphatic ester ethyl acetate was less emitted after 12 d by ambrosia beetle mutualists and other fungi (P < 0.02; see Suppl. Fig. S6 e), but not by bark beetle mutualists (*P* > 0.07).

### Adults and larvae of ambrosia beetles are attracted to their mutualist fungi by volatile cues

Adult female *X. saxesenii* were significantly attracted to the odor of their mutualistic fungus *R. canadensis* (*P* < 0.008; Fisher’s exact test including Bonferroni correction; see Suppl. Table S6 for further details; Fig. 6a), while none of the other fungi tested were attractive. Larvae were highly attracted to *D. sulphureus* (*P* < 0.001), the second mutualistic fungus of *X. saxesenii*. In *X. germanus*, adult beetles (*P* < 0.02) as well as larvae (*P* < 0.001) were attracted by the volatile bouquet of their mutualist, *Ambrosiella grosmanniae* (Fig. 6b). Larvae were also attracted by *A. hartigii* (*P* < 0.001), a close relative of *A. grosmanniae*, while they were not attracted by the entomopathogen *Beauveria bassiana* (Fig. 6b).

**Figure 6:**
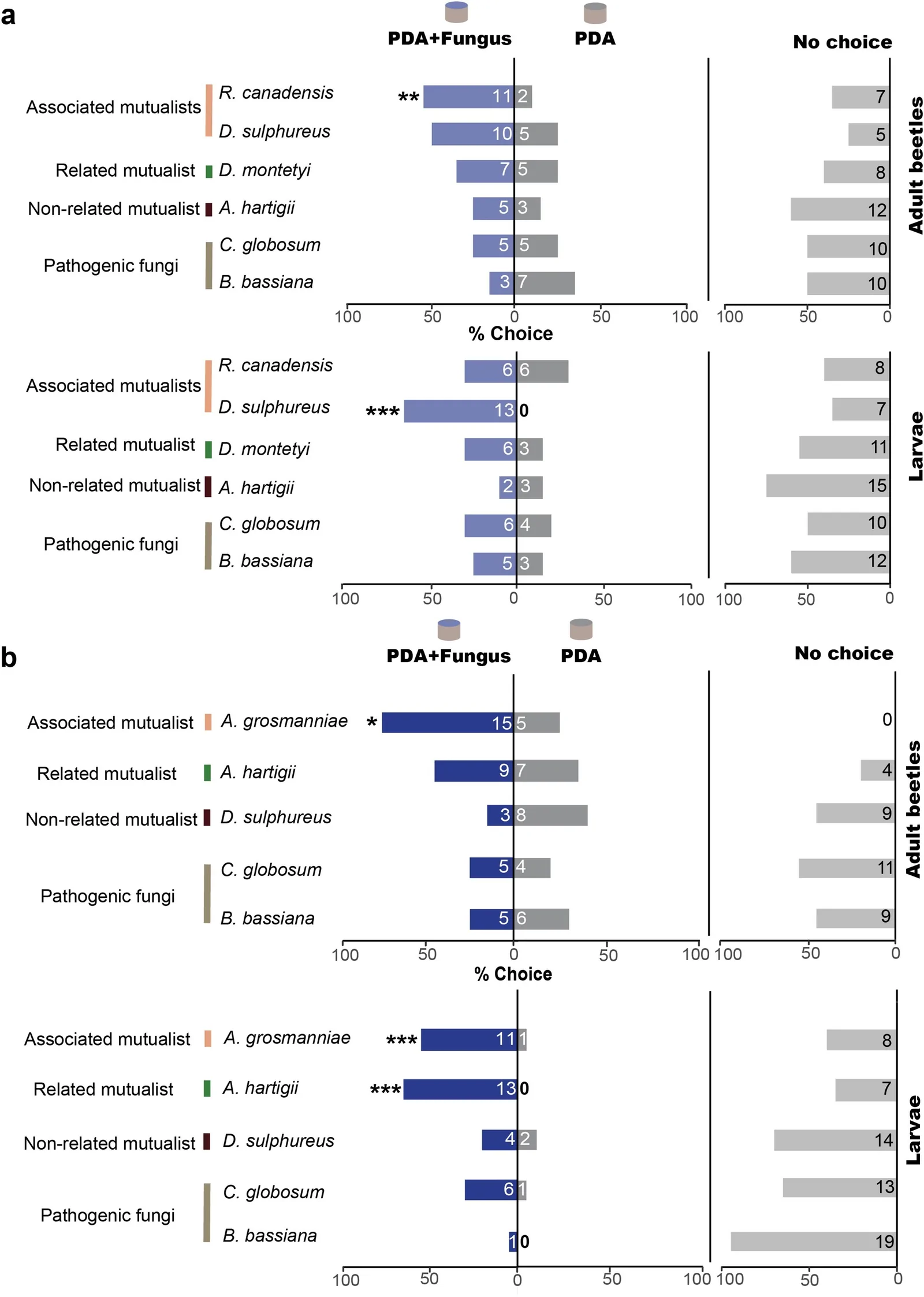
Larvae and adults of the ambrosia beetles *Xyleborinus saxesenii* and *Xylosandrus germanus* are attracted by volatile cues to their mutualist fungi. Adult beetles and larvae were allowed to choose between fungus-colonized agar of each species (PDA + Fungus, blue) and fungus-free agar (PDA, dark grey). Beetles that did not prefer either of the two choices were designated “no choice”. Numbers inside the bars indicate the total number of beetles and larvae making the indicated choice in each experiment. (a) *X. saxesenii*: Adult beetles were attracted only to the volatiles of the mutualist *R. canadensis*, but not to the volatiles of other mutualists (whether taxonomically related or unrelated), nor to insect pathogenic fungi (upper panel). In contrast, larvae of *Xyleborinus saxesenii* were attracted only to *D. sulphureus*, but not to *R. canadensis* and other fungi (lower panel). (b) *X. germanus*: Adult beetles were attracted only to the volatile profile of the mutualist *A. grosmanniae* but not to the volatile profile of closely related, unrelated and pathogenic fungi (upper panel). Larvae of *X. germanus* were attracted to *A. grosmanniae* and the closely related ambrosia beetle mutualist *A. hartigii*, but not to the other fungi tested (lower panel). Asterisks indicate significant differences between fungus-colonized agar and fungus-free agar (ns—*P* > 0.05, *P < 0.05, **P < 0.01, ***P < 0.001; Fisher’s exact test including Bonferroni correction). N = 20 with single beetle or larvae for each tested fungus.

## Discussion

Bark and ambrosia beetles are well-known for their close association with filamentous fungi mostly within the Ascomycota (Hulcr and Stelinski 2017). Many of these species are mutualists that support beetle fitness by providing nutrients and/or reducing the availability of chemical plant defenses (Batra 1967, Hammerbacher et al. 2013, Wadke et al. 2016, Six and Elser 2019, Lehenberger et al. 2021, 2025, Lichnock et al. 2026). To take full advantage of these contributions, beetles must be able to distinguish these mutualists from other fungal species. The volatile blends emitted from fungal mutualists have been shown to attract bark and ambrosia beetles (Hulcr et al. 2011, Egonyu and Torto 2018, Gugliuzzo et al. 2023), and a number of these compounds have been identified (Kandasamy et al. 2016, 2019, 2023, Ceriani-Nakamurakare et al. 2024). Yet, the detailed composition of those blends has only been studied in a few cases. Here, we examined the volatile profiles of 25 fungal mutualists of bark and ambrosia beetles and compared the blends emitted with those of commensal or antagonistic fungal associates. We found that mutualistic fungi as a group were strong emitters of aliphatic and aromatic alcohols and esters, and emitted a greater diversity of volatiles than non-mutualist fungi. Moreover, individual fungal species emitted characteristic blends of volatiles. Correspondingly, ambrosia beetle adults and larvae were shown to be specifically attracted to their fungal mutualists and not to mutualists of other beetles or to non-mutualistic associates.

Many species of filamentous fungi are known to emit volatile organic compounds, with various chemical classes reported (Schnürer et al. 1999, Morath et al. 2012, Müller et al. 2013, Hung et al. 2015, Kandasamy et al. 2016, 2019, Inamdar et al. 2020). In this study, the major classes emitted by bark and ambrosia beetle mutualists were aliphatic and aromatic alcohols and their esters. This finding is consistent with the scattered reports in the existing literature (Schnürer et al. 1999, Morath et al. 2012, Müller et al. 2013, Hung et al. 2015, Kandasamy et al. 2016, 2019, Inamdar et al. 2020, Ranger et al. 2021). These compound classes were released with greater diversity and in higher amounts (for individual compounds) compared to the non-mutualistic beetle symbionts. Aliphatic and aromatic alcohols and esters are widespread in filamentous fungi and yeasts (Pires et al. 2014). They are also emitted by yeast symbionts of insects, for instance in the fruit fly *Drosophila*, where they act as attractants (Christiaens et al. 2014). Apart from fungal attraction, some of these compounds may be also used by plants to attract animals to nectar and fruits [e.g. (Gibernau et al. 1997, Laska and Seibt 2002, Ehlers and Schulz 2023)]. These compound classes possess properties such as the requisite volatility and the widespread occurrence of olfactory sensory neurons responsive to them, which allows them to be frequently recruited for communication in interkingdom nutritional mutualisms between animals and plants or fungi (Mansourian and Stensmyr 2015, Biswas et al. 2023). A recent study identified other correlations between fungal lifestyles and the chemical classes of volatiles they emit (Guo et al. 2021). Beyond the aliphatic and aromatic compounds, bark and ambrosia beetle mutualists released a greater general diversity of volatile compounds than non-mutualist associates. Our results suggest that these substances likely play a role in maintaining mutualisms between insects and their symbiotic fungi. However, the breadth of our volatile profiles is limited by the use of pure PDMS tubes as sorbents, which may underrepresent more polar volatiles, such as ethanol, and overrepresent more non-polar volatiles, such as monoterpenes and sesquiterpenes.The majority of the beetle mutualists we examined were characterized by species-specific volatile profiles. For example, the closely related species in the genus *Raffaelea* and *Dryadomyces* each had a distinct volatile blend (Suppl. Figs S2 and S3). The two *Raffaelea* species had profiles dominated by aliphatic esters, but *R. canadensis* also had aliphatic alcohols and aromatic compounds. The two *Dryadomyces* species, *D. montetyi* and *D. sulphureus*, had aliphatic alcohols and esters, but also sesquiterpenes, with *D. sulphureus* emitting an aliphatic ketone in addition. Similar differences have been previously reported among fungal species (Larsen and Frisvad 1995, Müller et al. 2013), and even among strains of a single species (Buśko et al. 2014).

The specificity of these fungal volatile blends may contribute to the attraction of bark and ambrosia beetles, as demonstrated in previous studies (Hulcr et al. 2011, Kandasamy et al. 2019, Ranger et al. 2021, Gugliuzzo et al. 2023, Tobin et al. 2024). Here, we demonstrated that adult ambrosia beetles (*Xyleb. saxesenii* and *Xylos. germanus*) are attracted to the olfactory cues of their nutritional mutualists, as previously observed for *X. germanus* (Ranger et al. 2021), but not to the mutualists of other ambrosia beetles, regardless of whether their fungi are taxonomically related. The beetles were also not attracted to insect-pathogenic fungi (Nuotcl et al. 2019). These behaviors likely rely on fungal-specific volatile profiles that enable beetles to distinguish between fungal taxa allowing them to maintain associations with those best suited to their needs. The ambrosia beetle *X. saxesenii* is associated with two nutritionally important mutualistic fungi, *R. canadensis* and *D. sulphureus* (Biedermann and Taborsky 2011, Biedermann et al. 2013), but the individual benefit of these fungi for beetle fitness remains unknown. Adult *X. saxesenii* were highly attracted to *R. canadensis*, while larval beetles preferred *D. sulphureus*. This is in line with the different modes of feeding between larvae and adult *X. saxesenii* (De Fine Licht and Biedermann 2012) and the described succession of the two nutritional fungi within beetle nests (Diehl et al. 2023): *D. sulphureus* grows faster and appears first in the fungus gardens around the time when most larvae are developing, whereas *R. canadensis* occurs later in the breeding tunnels when adults are feeding. Whether these volatiles also attract the beetles in nature, for example, to already colonized host trees remain unknown. However, it has been shown that the ambrosia beetle *X. germanus* uses fungal volatiles as mass aggregation cues (possibly to overcome host-tree defenses) (Gugliuzzo et al. 2023). In contrast, field trails with individual volatiles did not enhance the attraction of *X. germanus* to ethanol and, in some cases, even reduced attraction to ethanol (Ranger et al. 2021).

Besides their function as attractants for beetle mutualists, the volatiles of bark and ambrosia beetle fungi may also serve as defenses to suppress the growth of substrate-competing and pathogenic fungi (Nuotcl et al. 2019), or even natural enemies such as nematodes. Indeed, various volatiles emitted by fungi are known to have antifungal (Schalchli et al. 2011, Hung et al. 2015, Schalchli et al. 2015), anti-bacterial and nematicidal (Denyer et al. 1986, Lee et al. 2023) activities. Among the most abundant volatiles identified in this study, the activity of 2-phenylethanol, ethanol, isoamyl alcohol, ethyl acetate and isoamyl acetate against fungi and bacteria have been previously described (Ando et al. 2015, Lens et al. 2016, Majdabadi et al. 2018, Ruiz-Moyano et al. 2020, Kleinwächter et al. 2021, Lehenberger et al. 2021). Other fungal volatiles, such as the monoterpene alcohols citronellol and nerol (emitted from the ambrosia beetle mutualist *A. hylecoeti*), have well-known activities against microbes as well (Pereira et al. 2015, Santos et al. 2017, Wang et al. 2020, Lehenberger et al. 2025). The individual roles of specific compounds and possible dual roles of the blend of bark and ambrosia beetle mutualists in attraction and defense need further study.

In this study, we also tested for changes in volatile emission as fungal cultures aged, since this might occur under natural conditions as the nutrient content of the substrate declines and influence volatile communication with beetle symbionts. Ambrosia beetle mutualists colonize the beetle tunnels within the xylem, but are believed to penetrate the woody tissues only for a few millimeters (Francke-Grosmann 1967, Lehenberger et al. 2021). There, they extend their mycelia and depolymerize the major woody polysaccharides and ingest nutrients and translocate them to nutritional structures on the tunnel walls (Batra 1963, Francke-Grosmann 1967, Batra 1985, De Fine Licht and Biedermann 2012, Lehenberger et al. 2018). Once this thin layer of substrate is depleted, the beetles and their larvae must constantly enlarge the breeding system to renew the availability of fresh surfaces for nutrient acquisition. On a Petri plate in a laboratory setting, fungi are expected to run out of nutrients and slow their growth and rate of metabolism (Wallis et al. 2012, Pires et al. 2014, Holt et al. 2019). However, we found no correlation between the availability of free amino acids in the fungal mycelium and the emission of volatile compounds over time in our study. In particular, we looked for a link between the levels of amino acids, such as phenylalanine (Phe) and leucine (Leu), which are close precursors of 2-phenylethanol and isoamyl alcohol, respectively, and emissions of these volatiles. However, we found no significant relationships. Nevertheless, under natural conditions, reduced volatile emissions might be correlated with reduced nutrient availability, and could thus function as a location-specific cue for beetles and larvae to extend their tunnels or disperse from their nests. Adult *X. saxesenii* are known to leave their mother’s nest when fungal growth and larval numbers decline [e.g. (Biedermann and Taborsky 2011)]. Possibly they sense these declines through changes in the volatile profiles of their mutualistic fungi.

In comparing amino acid content among the fungal species tested, we found that mutualist fungi of bark beetles had significantly higher levels of essential amino acids than both ambrosia beetle mutualists and non-mutualist fungal associates. This may suggest that bark beetles receive more amino acids from their fungal symbionts than ambrosia beetles receive from theirs. This would be surprising since ambrosia beetles feed almost exclusively on fungal tissues whereas bark beetles are regarded to feed on phloem as well as fungi (Six 2012). Comparisons of other nutrients between these fungal groups, such as vitamins, sugars, sterols and fatty acids, would help in establishing the relative importance of these fungi in supplying the nutritional needs of their beetle partners.

Our results show that bark and ambrosia beetle mutualists emit a higher diversity of volatile compounds than non-mutualist fungi. Their volatile bouquets consist especially of aliphatic and aromatic alcohols and esters, and individual compounds are emitted in greater amounts than in the other fungi analyzed. Individual fungal species produced distinct volatile mixtures, allowing them to be distinguished by their beetle hosts. In fact, we found that two ambrosia beetle species were specifically attracted to their fungal mutualists based on their volatile cues. This suggests that the major classes of volatiles released by the fungal mutualists of bark and ambrosia beetles may be important signals in maintaining these mutualisms. However, as all fungi were grown on PDA, the volatile profiles of this study may not fully reflect VOC production under natural host-associated conditions, highlighting the need for future studies using host-derived substrates or woody material for fungal inoculation and volatile collection. Whether these volatiles also protect the mutualism from competing microbes or natural enemies, and how fungal strain specificity affects volatile composition, remains to be investigated.

## Supplementary Material

fiag115_Supplemental_Files

## Data Availability

All data generated or analyzed during this study are included in this published article (and its Supplementary material files).
