# Supplementary material for: Bark and ambrosia beetle fungi emit a greater diversity of volatile organic compounds than other fungi
Source: FEMS Microbiol Ecol. 2026 Oct 5;102(11):fiag115. doi: 10.1093/femsec/fiag115 (PMC13673984; doi:10.1093/femsec/fiag115)
Supplement: Supplementary file 1 [file fiag115_supplemental_files.zip › SI Methods Lehenberger et al.docx]

**Methods**

**DNA extraction and LSU / ITS / βT barcoding**

Fungal samples were first homogenized by grinding in liquid nitrogen. DNA was extracted afterwards using the NORGEN Biotek Corp. fungi/yeast genomic DNA isolation kit. Primarily, we used the large subunit (LSU) of the ribosomal RNA gene for the identification and phylogenetic placement of isolates (Fig. 1, Suppl. Table S3). For amplification, we used the common primers LROR-F (GTACCCGCTGAACTTAAGC) and LR5-R (ATCCTGAGGGAAACTTCG) (1, 2). As annealing temperature, we applied 55.5°C. The resulting sequence lengths were about 830 to 880 bp.

As few samples repeatedly failed to amplify using the LSU primers, we either amplified the internal transcribed spacer (ITS) of the ribosomal RNA or the beta-tubulin gene (βT) to confirm the specimen’s identity. The ITS region was amplified following the protocol in (3). For amplifying βT, we used the primers T10-F (ACGATAGGTTCACCTCCAGAC) and Bt2b-R (ACCCTCAGTGTAGTGACCCTTGGC) (4, 5) and 57.5°C as annealing temperature. Sequence lengths contained around 300 to 400 bp.

We used a similar master-mix for all PCR reactions for barcoding the LSU, ITS and βT region ((PCR Master-mix for 50 µl: 25 µl 2x phusion high-fidelity PCR master mix with GC buffer (Thermo Scientific™, Germany), 2.5 µl forward primer 1 (10 µM, Eurofins Genomics, Germany), 2.5 µl reverse primer 2 (10 µM, Eurofins Genomics, Germany), 18 µl ddH20, 2 µl template (usually 1:10 diluted). We applied the following PCR conditions to all three genetic markers: 98°C for 30 s, followed by 35 cycles at 98°C for 10 s, 55.5°C, 54.5°C or 57.5°C (for LSU, ITS and βT, respectively) for 30 s and 72°C for 20s, ending with 72°C for 10 min including a storage temperature of 5°C.

For purifying extracted DNA, we used the Wizard® SV gel and PCR clean-up system (Promega, Germany) after performing gel electrophoresis. Sequencing was accomplished by Euroﬁns (Eurofins Genomics, Germany). Each sequence quality was verified using the "SnapGene® Viewer” 3.2 (SnapGene software, from GSL Biotech; available at snapgene.com) and afterwards aligned with the respective forward and reverse sequence for each fungal species using the R software (version 4.5.1) and the packages “Biostrings” and “stringr” (6, 7). Then, we BLASTed final sequences against the NCBI database to assign the taxonomy to species level (>99% identity match) (8).

**Maximum likelihood analyses**

We systematically compared multiple sequence alignment methods (MSA) in aim to identify the optimal alignment for downstream phylogenetic analyses using the software R (version 4.5.1). In general, four algorithms were tested using the package “msa” (9): Clustal Omega, ClustalW, Muscle, and MAFFT, which were all chosen for their widespread use and robust performance in DNA sequence alignments (10-12). To improve alignment quality by removing poorly aligned or ambiguously aligned regions, Gblocks Version 0.91b (13) was applied to each alignment using default parameters to filter out positions with high gaps or low conservation. Next, we assessed the quality of each alignment and its corresponding Gblocks-filtered version using a set of four quality metrics: mean Shannon entropy per position, gap percentage, proportion of fully informative positions, and log-likelihood values obtained from maximum likelihood phylogenetic tree inference using neighbor-joining starting trees as implemented in the packages “Biostrings”, “entropy”, “seqinr”, and “phangorn” (6, 14-20). The best Gblocks-filtered version (MAFFT) was selected based on the highest overall quality scores for further analyses. Then, we applied the model finder implemented in IQ-Tree Version 2.2.2 (21) to identify the best-fitting model for our sequence data (TIM3+F+I+G4). Phylogenetic trees were inferred using maximum likelihood with robust branch support estimation, including 8,000 ultrafast bootstrap replicates and Shimodaira-Hasegawa approximate likelihood ratio test (SH-aLRT) branch tests (22). We rooted the resulting tree based on a fungal outgroup (*Aspergillus flavus*, *A. niger*, *Penicillium commune*) using the “ape” package (23). Branch lengths were square-root transformed to improve visualization. Therefore, the scale bar was omitted because transformed lengths are not directly proportional to the number of substitutions per site. Additionally, the packages “dplyr”, “tidyverse”, and “stringr” (7, 24, 25) were used for additional data processing and filtering. For the visualization of the final maximum likelihood tree, we used the package ggtree (26) and the software FigTree (http://tree.bio.ed.ac.uk/software/ﬁgtree), as well as the software Adobe Illustrator (version CS5) for further modifications.

**Analyses of volatile organic compounds (VOCs) from fungal cultures**

To evaluate statistically significant differences between the emitted volatiles of fungal groups as well as individual fungal species, we used a series of fitted generalized linear models (see ref. (27) for further details on model evaluation and applied packages). When more than 35% of observations within a grouped VOC dataset were zeros (i.e., fungi that did not emit the respective volatile compound), the data were treated as binary (absence/presence). For statistical inference, we applied exact Fisher´s tests to account for sample sizes and potential imbalances between groups. A global Fisher test was first performed to evaluate overall differences among the groups. Subsequently, pairwise Fisher’s tests were conducted between all group combinations, with P values adjusted for multiple comparisons using the Holm method. For count data such as the absolute number of volatile compounds, we first performed global as well as pairwise Permutational Multivariate Analysis of Variance (PERMANOVA) with 999 permutations based on Bray-Curtis dissimilarities calculated from log1p-transformed data. To confirm that group centroids were not driven by differences in group dispersions, we additionally tested for multivariate homogeneity of group dispersions (beta-dispersion), also with 999 permutations. For pairwise PERMANOVA comparisons, P-values were adjusted for multiple testing using the Benjamini-Hochberg method implemented via the package “RVAideMemoire” (28). Then, we applied a GLM with a Poisson distribution and a log link function to explore further differences. Here, overdispersion was assessed using the dispersion test implemented in the “AER” package (29). When overdispersion was detected, the model was extended to a Negative Binomial GLM using the “MASS” package (30) to improve model fit. Model adequacy was evaluated by examining the dispersion parameter calculated from Pearson residuals and by comparing models based on the AICc. Post-hoc pairwise comparisons between treatments were conducted using Tukey’s test with adjustment for multiple comparisons. For the statistical analysis of individual volatile compounds as well as amino acids at two collection time points (6 d and 12 d), Student’s t-test, Welch’s t-test, or the Wilcoxon rank-sum test was applied, depending on data distribution and variance homogeneity.

Additionally, we visualized the abundance of each individual volatile within the chosen chemical groups as well as the respective absolute number of volatiles emitted using heatmaps and boxplots, where we applied the following packages: “plyr” (31), “dplyr” (24), “pheatmap” (32), “viridis” (33), “RColorBrewer” (34), “gplots” (35), and “reshape2” (36). Finally, all plots were modified using the software Adobe Illustrator (version CS5).

**References**

1.	Rehner SA and Samuels GJ. Taxonomy and phylogeny of Gliocladium analysed from nuclear large subunit ribosomal DNA sequences*.* *Mycological Research*. 1994. **98**(6): p. 625–634.

2.	Vilgalys R and Hester M. Rapid genetic identification and mapping of enzymatically amplified ribosomal DNA from several Cryptococcus species*.* *Journal of bacteriology*. 1990. **172**(8): p. 4238–4246.

3.	Lehenberger M, *Molecular identification and enzymatic profiling of Trypodendron (Curculionidae: Xyloterini) ambrosia beetle-associated fungi of the genus Phialophoropsis (Microascales: Ceratocystidaceae)*. 2018.

4.	Glass NL and Donaldson GC. Development of primer sets designed for use with the PCR to amplify conserved genes from filamentous ascomycetes*.* *Applied and environmental microbiology*. 1995. **61**(4): p. 1323–1330.

5.	O'Donnell K and Cigelnik E. Two divergent intragenomic rDNA ITS2 types within a monophyletic lineage of the fungusfusariumare nonorthologous*.* *Molecular phylogenetics and evolution*. 1997. **7**(1): p. 103–116.

6.	Pages H, Aboyoun P, Gentleman R, DebRoy S, Pagès MH, DataImport D, BSgenome S, XStringSet-class R, MaskedXString-class R, and XStringSet-io R. Package ‘Biostrings’*.* *Bioconductor*. 2013. **18129**: p. 501.

7.	Wickham H, *stringr: simple, consistent wrappers for common string operations. R package version 1.4. 0*. 2019.

8.	Altschul SF, Gish W, Miller W, Myers EW, and Lipman DJ. Basic local alignment search tool*.* *Journal of molecular biology*. 1990. **215**(3): p. 403–410.

9.	Bodenhofer U, Bonatesta E, Horejš-Kainrath C, and Hochreiter S. msa: an R package for multiple sequence alignment*.* *Bioinformatics*. 2015. **31**(24): p. 3997–3999.

10.	Sievers F, Wilm A, Dineen D, Gibson TJ, Karplus K, Li W, Lopez R, McWilliam H, Remmert M, and Söding J. Fast, scalable generation of high‐quality protein multiple sequence alignments using Clustal Omega*.* *Molecular systems biology*. 2011. **7**(1): p. 539.

11.	Katoh K and Standley DM. MAFFT multiple sequence alignment software version 7: improvements in performance and usability*.* *Molecular biology and evolution*. 2013. **30**(4): p. 772–780.

12.	Larkin MA, Blackshields G, Brown NP, Chenna R, McGettigan PA, McWilliam H, Valentin F, Wallace IM, Wilm A, and Lopez R. Clustal W and Clustal X version 2.0*.* *bioinformatics*. 2007. **23**(21): p. 2947–2948.

13.	Castresana J. Selection of conserved blocks from multiple alignments for their use in phylogenetic analysis*.* *Molecular biology and evolution*. 2000. **17**(4): p. 540–552.

14.	Schneider TD and Stephens RM. Sequence logos: a new way to display consensus sequences*.* *Nucleic acids research*. 1990. **18**(20): p. 6097–6100.

15.	Shannon CE. A mathematical theory of communication*.* *The Bell system technical journal*. 1948. **27**(3): p. 379–423.

16.	Yang Z. PAML 4: phylogenetic analysis by maximum likelihood*.* *Molecular biology and evolution*. 2007. **24**(8): p. 1586–1591.

17.	Paradis E, Claude J, and Strimmer K. APE: analyses of phylogenetics and evolution in R language*.* *Bioinformatics*. 2004. **20**(2): p. 289–290.

18.	Charif D, Lobry JR, Necsulea A, Palmeira L, Penel S, Perriere G, and Penel MS. Package ‘seqinr’*.* *Biological Sequences Retrieval and Analysis*. 2017.

19.	Schliep KP. phangorn: phylogenetic analysis in R*.* *Bioinformatics*. 2011. **27**(4): p. 592–593.

20.	Hausser J, Strimmer K, and Strimmer MK. Package ‘entropy’*.* *R Foundation for Statistical Computing: Vienna, Austria*. 2012.

21.	Minh BQ, Schmidt HA, Chernomor O, Schrempf D, Woodhams MD, Von Haeseler A, and Lanfear R. IQ-TREE 2: new models and efficient methods for phylogenetic inference in the genomic era*.* *Molecular biology and evolution*. 2020. **37**(5): p. 1530–1534.

22.	Hoang DT, Chernomor O, Von Haeseler A, Minh BQ, and Vinh LS. UFBoot2: improving the ultrafast bootstrap approximation*.* *Molecular biology and evolution*. 2018. **35**(2): p. 518–522.

23.	Paradis E, Blomberg S, Bolker B, Brown J, Claude J, Cuong HS, Desper R, and Didier G. Package ‘ape’*.* *Analyses of phylogenetics and evolution, version*. 2019. **2**(4): p. 47.

24.	Wickham H and Wickham MH. dplyr: A Grammar of Data Manipulation*.* *R package version 0.8.5*. 2020.

25.	Wickham H, Averick M, Bryan J, Chang W, McGowan LDA, François R, Grolemund G, Hayes A, Henry L, and Hester J. Welcome to the Tidyverse*.* *Journal of open source software*. 2019. **4**(43): p. 1686.

26.	Yu G, Smith DK, Zhu H, Guan Y, and Lam TTY. ggtree: an R package for visualization and annotation of phylogenetic trees with their covariates and other associated data*.* *Methods in ecology and evolution*. 2017. **8**(1): p. 28–36.

27.	Lehenberger M, Pan Y, Ungerer S, Reichelt M, Pemp D, Paetz C, Lehenberger J, Gentsch N, Feistel F, and Gros P. Fungal symbiont of an ambrosia beetle possesses high nutrient content and suppresses competing fungi with antimicrobial compounds*.* *The ISME Journal*. 2025. **19**(1): p. wraf258.

28.	Herv M. RVAideMemoire: testing and plotting procedures for biostatistics*.* *R package version 0.9-69*. 2018. **3**.

29.	Kleiber C, Zeileis A, and Zeileis MA. Package ‘aer’*.* *R package version*. 2020. **1**(4).

30.	Venables W and Ripley B, *Modern Applied Statistics with S, Springer, New York: ISBN 0-387-95457-0*. 2002.

31.	Wickham H. The split-apply-combine strategy for data analysis*.* *Journal of statistical software*. 2011. **40**: p. 1–29.

32.	Kolde R, *pheatmap: Pretty Heatmaps. R package version 1.0. 12*. 2019.

33.	Garnier S, Ross N, Rudis B, Sciaini M, and Scherer C. viridis: Default Color Maps from ‘matplotlib’*.* *R package version 0.5*. 2018. **1**: p. 2018.

34.	Neuwirth E and others. RColorBrewer: colorbrewer palettes*.* *R package version*. 2014. **1**(2).

35.	Warnes GR, Bolker B, Bonebakker L, Gentleman R, Liaw WHA, Lumley T, Maechler M, Magnusson A, Moeller S, Schwartz M, and others. Gplots: various R programming tools for plotting data. 2016*.* *R package version*. 2014. **2**(0).

36.	Wickham H. Reshaping data with the reshape package*.* *Journal of statistical software*. 2007. **21**: p. 1–20.
