# Supplementary material for: Bark and ambrosia beetle fungi emit a greater diversity of volatile organic compounds than other fungi
Source: FEMS Microbiol Ecol. 2026 Oct 5;102(11):fiag115. doi: 10.1093/femsec/fiag115 (PMC13673984; doi:10.1093/femsec/fiag115)
Supplement: Supplementary file 1 [file fiag115_supplemental_files.zip › Suppl Table_S6.docx]

**Supplementary table S6_p-Values and statistics**

Statistical analyses are generally sorted by figures

**Overview chemical groups and individual compounds (Day 6 and Day 12)**

**1. Aliphatic alcohols**

Ethanol, 1-Methoxy-2-propanol, 1-Butanol-3-methyl, 3-Octanol, Isobutyl alcohol, 1-Butanol, 3-Methyl-2-pentanol, 3-Cyclohexen-1-ol

**2. Aliphatic esters**

Ethyl acetate, Isoamyl acetate, Hexyl acetate, Ethyl propionate, Isobutyl acetate, Propyl acetate, Ethyl isobutyrate, Methyl isovalerate, Ethyl butyrate, Octyl acetate, Butyl acetate, Lauryl acetate, Tetradecyl acetate, Isopropyl acetate, Methyl isobutyrate, Propyl propionate, Isobutyl propionate, Amyl propionate, Isoamyl propionate, 2-Methylbutyl acetate, 2-Methoxyethyl acetate, Ethyl 2-methylbutyrate, Nonyl acetate, Pentadecyl acetate, Gamma decalactone

**3. Aromatic alcohols**

Cresol, Butylhydroxy toluene, Phenylethyl alcohol

**4. Aromatic esters**

Methyl benzoate, Phenethyl acetate, Methylphenyl acetate, 2-Phenylethyl propionate

**5. Monoterpenes**

Beta Ocimene, Nerol, Linalool, Isopulegol, Rhodinol, Citronellol, Citral, Beta Phellandrene, Citronellic acid, Beta Linalool, Beta Myrcene, Beta Pinene, Linalyl oxide, Citronelly acetate, Neryl acetate, Geranyl acetate

**6. Sesquiterpenes**

Beta Elemene, Aristolene, Beta Gurjunene, Germacrene D, Beta Curcumene, Valencene, Alpha Selinene, Alpha Panasinsene, Caryophyllene 1, Caryophyllene 2, Caryophyllene oxide, Gamma Elemene, 2,3-Dihydrofarnesyl acetate

**7. Aromatics**

Styrene, Benzaldehyde

**8. Aliphatic ketones**

3-Octanone, 3-Methyl-2-pentanone, 2-Butanone

**9. Others**

RT 10.290, RT 11.933, RT 12.307, RT 14.207, RT 15.797, RT 19.313, RT 20.880, RT 21.030, RT 21.233, RT 24.407, RT 24.847, RT 24.883, RT 25.050, Decanal

**Fig. 2a**

Global PERMANOVA

| **Transformation** | **NMDS_Stress** | **PERMANOVA_F** | **PERMANOVA_R2** | **PERMANOVA_p** | **Betadisp_F** | **Betadisp_p** |
| --- | --- | --- | --- | --- | --- | --- |
| log1p | 0.159407005 | 3.383433923 | 0.200399631 | 0.001 | 0.588754008 | 0.583 |

Pairwise PERMANOVAS

| **Group1** | **Group2** | **PERMANOVA_F** | **PERMANOVA_R2** | **PERMANOVA_p_raw** | **Betadisp_F** | **Betadisp_p** | **PERMANOVA_p_adj** |
| --- | --- | --- | --- | --- | --- | --- | --- |
| Ambrosia | Bark | 1.801222 | 0.086592 | 0.146 | 0.018934 | 0.887 | 0.146 |
| Ambrosia | others | 4.419951 | 0.206347 | 0.001 | 0.691167 | 0.413 | 0.003 |
| Bark | others | 3.715368 | 0.171094 | 0.018 | 1.092903 | 0.32 | 0.027 |

**Fig. 2b (left figure)**

| Model | AIC | AICc |
| --- | --- | --- |
| Poisson | 172.8423 | 173.7654 |
| Negative Binomial | 165.3981 | 166.9981 |

| Model | Dispersion_Parameter |
| --- | --- |
| Poisson | 2.086723 |
| Negative Binomial | 1.021728 |

| Comparison | Estimate | Lower_CI | Upper_CI | z_value | p_value | Significance |
| --- | --- | --- | --- | --- | --- | --- |
| Bark beetle fungi - Ambrosia beetle fungi | -0.38886 | -0.88444 | 0.106727 | -1.8352 | 0.156284 | ns |
| Others - Ambrosia beetle fungi | -1.29863 | -1.92371 | -0.67356 | -4.85919 | 3.14E-06 | *** |
| Others - Bark beetle fungi | -0.90978 | -1.54282 | -0.27673 | -3.36132 | 0.002299 | ** |

**Right figure**

| Model | AIC | AICc |
| --- | --- | --- |
| Poisson | 154.5897 | 155.5128 |
| Negative Binomial | 153.1335 | 154.7335 |

| Model | Dispersion_Parameter |
| --- | --- |
| Poisson | 1.643352 |
| Negative Binomial | 1.042317 |

| Comparison | Estimate | Lower_CI | Upper_CI | z_value | p_value | Significance |
| --- | --- | --- | --- | --- | --- | --- |
| Bark beetle fungi - Ambrosia beetle fungi | -0.16069 | -0.65273 | 0.33134 | -0.76365 | 0.723097 | ns |
| Others - Ambrosia beetle fungi | -1.08603 | -1.72361 | -0.44846 | -3.98297 | 0.000164 | *** |
| Others - Bark beetle fungi | -0.92534 | -1.56288 | -0.2878 | -3.3938 | 0.002043 | ** |

**Fig. 2c**

**Esters**

| Model | AIC | AICc | Dispersion |
| --- | --- | --- | --- |
| Poisson | 147.6738 | 148.5968 | 2.362514 |
| NegBinomial | 136.7698 | 138.3698 | 1.032335 |

| term | estimate | std.error | statistic | p.value |
| --- | --- | --- | --- | --- |
| (Intercept) | 1.704748 | 0.231627 | 7.359879 | 1.84E-13 |
| ClassificationBark beetle fungi | -0.51912 | 0.337155 | -1.53972 | 0.123629 |
| ClassificationOthers | -1.50408 | 0.428918 | -3.50668 | 0.000454 |

| Model | Comparison | Estimate | Lower_CI | Upper_CI | z_value | p_value | Significance |
| --- | --- | --- | --- | --- | --- | --- | --- |
| Negative Binomial | Bark beetle fungi - Ambrosia beetle fungi | -0.51912 | -1.30682 | 0.26857 | -1.53972 | 0.269654 | ns |
| Negative Binomial | Others - Ambrosia beetle fungi | -1.50408 | -2.50616 | -0.502 | -3.50668 | 0.001243 | ** |
| Negative Binomial | Others - Bark beetle fungi | -0.98495 | -2.00424 | 0.034331 | -2.25761 | 0.060852 | ns |

**Alcohols**

| Model | AIC | AICc | Dispersion |
| --- | --- | --- | --- |
| Poisson | 104.8373 | 105.7604 | 0.664218 |
| NegBinomial | 106.8379 | 108.4379 | 0.664194 |

| term | estimate | std.error | statistic | p.value |
| --- | --- | --- | --- | --- |
| (Intercept) | 0.875469 | 0.204124 | 4.288903 | 1.8E-05 |
| ClassificationBark beetle fungi | 0.364222 | 0.260734 | 1.396908 | 0.162441 |
| ClassificationOthers | -0.50774 | 0.344369 | -1.47442 | 0.140368 |

| Model | Comparison | Estimate | Lower_CI | Upper_CI | z_value | p_value | Significance |
| --- | --- | --- | --- | --- | --- | --- | --- |
| Poisson | Bark beetle fungi - Ambrosia beetle fungi | 0.364222 | -0.24471 | 0.973151 | 1.396908 | 0.339234 | ns |
| Poisson | Others - Ambrosia beetle fungi | -0.50774 | -1.312 | 0.296507 | -1.47442 | 0.300095 | ns |
| Poisson | Others - Bark beetle fungi | -0.87197 | -1.62236 | -0.12157 | -2.7138 | 0.01791 | * |

**Fig. 3**

**2-Phenylethanol**

| Statistic | p_value |
| --- | --- |
|  | 0.0075188 |

| Group1 | Group2 | p.value | p.adj | Signif |
| --- | --- | --- | --- | --- |
| Ambrosia beetle | Bark beetle | 0.06346749 | 0.12693498 |  |
| Ambrosia beetle | Others | 0.3498452 | 0.3498452 |  |
| Bark beetle | Others | 0.00452489 | 0.01357466 | * |

**Phenethyl acetate**

| Statistic | p_value |
| --- | --- |
|  | 0.04708569 |

| Group1 | Group2 | p.value | p.adj |
| --- | --- | --- | --- |
| Ambrosia beetle | Bark beetle | 0.66992141 | 0.66992141 |
| Ambrosia beetle | Others | 0.03250774 | 0.09752322 |
| Bark beetle | Others | 0.09411765 | 0.18823529 |

**Ethanol**

Modell

Sqrt_Normal

| Resid. Df | Resid. Dev | Df | Deviance | Pr(>Chi) |
| --- | --- | --- | --- | --- |
| 29 | 709.8156 |  |  |  |
| 27 | 545.8559 | 2 | 163.9598 | 0.017335 |

| contrast | estimate | SE | df | t.ratio | p.value | Signif |
| --- | --- | --- | --- | --- | --- | --- |
| Ambrosia beetle - Bark beetle | -4.82979 | 1.964583 | 27 | -2.45843 | 0.034388 | * |
| Ambrosia beetle - Others | 0.045069 | 2.065916 | 27 | 0.021815 | 0.982756 |  |
| Bark beetle - Others | 4.874856 | 2.020945 | 27 | 2.412166 | 0.034388 | * |

**Isoamyl alcohol**

Modell

Sqrt_Normal

| Resid. Df | Resid. Dev | Df | Deviance | Pr(>Chi) |
| --- | --- | --- | --- | --- |
| 29 | 675.2638 |  |  |  |
| 27 | 531.3859 | 2 | 143.878 | 0.025855 |

| contrast | estimate | SE | df | t.ratio | p.value | Signif |
| --- | --- | --- | --- | --- | --- | --- |
| Ambrosia beetle - Bark beetle | -2.59278 | 1.938369 | 27 | -1.33761 | 0.387257 |  |
| Ambrosia beetle - Others | 2.794994 | 2.03835 | 27 | 1.371204 | 0.369717 |  |
| Bark beetle - Others | 5.387774 | 1.993979 | 27 | 2.702022 | 0.030612 | * |

**Isobutyl alcohol**

| Statistic | p_value |
| --- | --- |
|  | 0.00041308 |

| Group1 | Group2 | p.value | p.adj | Signif |
| --- | --- | --- | --- | --- |
| Ambrosia beetle | Bark beetle | 0.0075188 | 0.01503759 | * |
| Ambrosia beetle | Others | 1 | 1 |  |
| Bark beetle | Others | 0.00138128 | 0.00414384 | ** |

**Ethyl acetate**

Modell

Sqrt_Normal

| Resid. Df | Resid. Dev | Df | Deviance | Pr(>Chi) |
| --- | --- | --- | --- | --- |
| 29 | 4410.789 |  |  |  |
| 27 | 3495.395 | 2 | 915.3934 | 0.029146 |

| contrast | estimate | SE | df | t.ratio | p.value | Signif |
| --- | --- | --- | --- | --- | --- | --- |
| Ambrosia beetle - Bark beetle | 8.707917 | 4.97141 | 27 | 1.751599 | 0.205007 |  |
| Ambrosia beetle - Others | 13.63069 | 5.227834 | 27 | 2.60733 | 0.037824 | * |
| Bark beetle - Others | 4.92277 | 5.114035 | 27 | 0.9626 | 0.60626 |  |

**Isoamyl acetate**

| Statistic | p_value |
| --- | --- |
|  | 0.01131168 |

| Group1 | Group2 | p.value | p.adj | Signif |
| --- | --- | --- | --- | --- |
| Ambrosia beetle | Bark beetle | 0.18266254 | 0.31424149 |  |
| Ambrosia beetle | Others | 0.00547749 | 0.01643248 | * |
| Bark beetle | Others | 0.15712074 | 0.31424149 |  |
|  |  |  |  |  |

**Fig. 4_Isoamyl alcohol**

Ambrosia beetle fungi

Modell

Sqrt_Normal

| Resid. Df | Resid. Dev | Df | Deviance | Pr(>Chi) | p.value_formatted | Significance |
| --- | --- | --- | --- | --- | --- | --- |
| 49 | 938.7621 |  |  |  | NA |  |
| 40 | 45.68882 | 9 | 893.0733 | 1.7E-162 | 1.6931853292e-162 | *** |

| contrast | estimate | SE | df | t.ratio | p.value | p.value_formatted | Significance |
| --- | --- | --- | --- | --- | --- | --- | --- |
| Alloascoidea_hylecoeti - Ambrosiella_grosmanniae | 6.098704 | 0.675935 | 40 | 9.022618 | 1.51E-09 | 1.5050302116e-09 | *** |
| Alloascoidea_hylecoeti - Ambrosiella_hartigii | 7.627124 | 0.675935 | 40 | 11.28381 | 2.85E-12 | 2.8523849949e-12 | *** |
| Alloascoidea_hylecoeti - Ambrosiella_roeperi | 8.694953 | 0.675935 | 40 | 12.86359 | 5.14E-13 | 5.1425530501e-13 | *** |
| Alloascoidea_hylecoeti - Fusarium_euwallaceae | 7.406382 | 0.675935 | 40 | 10.95724 | 6.25E-12 | 6.2481131380e-12 | *** |
| Alloascoidea_hylecoeti - Graphium_euwallaceae | 3.682998 | 0.675935 | 40 | 5.448745 | 0.000112 | 1.1229169911e-04 | *** |
| Alloascoidea_hylecoeti - Raffaelea_amase | 9.902437 | 0.675935 | 40 | 14.64998 | 4.66E-13 | 4.6584958113e-13 | *** |
| Alloascoidea_hylecoeti - Raffaelea_canadensis | -1.88982 | 0.675935 | 40 | -2.79586 | 0.172544 | 1.7254367669e-01 | |
| Alloascoidea_hylecoeti - Raffaelea_montetyi | -1.84999 | 0.675935 | 40 | -2.73693 | 0.193906 | 1.9390645005e-01 | |
| Alloascoidea_hylecoeti - Raffaelea_sulphurea | 1.236118 | 0.675935 | 40 | 1.828753 | 0.713734 | 7.1373423074e-01 | |
| Ambrosiella_grosmanniae - Ambrosiella_hartigii | 1.52842 | 0.675935 | 40 | 2.261194 | 0.434846 | 4.3484579257e-01 | |
| Ambrosiella_grosmanniae - Ambrosiella_roeperi | 2.59625 | 0.675935 | 40 | 3.840975 | 0.013855 | 1.3855389982e-02 | * |
| Ambrosiella_grosmanniae - Fusarium_euwallaceae | 1.307678 | 0.675935 | 40 | 1.934621 | 0.646674 | 6.4667376656e-01 | |
| Ambrosiella_grosmanniae - Graphium_euwallaceae | -2.41571 | 0.675935 | 40 | -3.57387 | 0.028247 | 2.8247058038e-02 | * |
| Ambrosiella_grosmanniae - Raffaelea_amase | 3.803733 | 0.675935 | 40 | 5.627364 | 6.38E-05 | 6.3799973708e-05 | *** |
| Ambrosiella_grosmanniae - Raffaelea_canadensis | -7.98853 | 0.675935 | 40 | -11.8185 | 1.05E-12 | 1.0547118734e-12 | *** |
| Ambrosiella_grosmanniae - Raffaelea_montetyi | -7.94869 | 0.675935 | 40 | -11.7595 | 1.15E-12 | 1.1517453657e-12 | *** |
| Ambrosiella_grosmanniae - Raffaelea_sulphurea | -4.86259 | 0.675935 | 40 | -7.19387 | 4.31E-07 | 4.3115225346e-07 | *** |
| Ambrosiella_hartigii - Ambrosiella_roeperi | 1.06783 | 0.675935 | 40 | 1.579781 | 0.849816 | 8.4981642736e-01 | |
| Ambrosiella_hartigii - Fusarium_euwallaceae | -0.22074 | 0.675935 | 40 | -0.32657 | 0.999999 | 9.9999900337e-01 | |
| Ambrosiella_hartigii - Graphium_euwallaceae | -3.94413 | 0.675935 | 40 | -5.83507 | 3.3E-05 | 3.2959002620e-05 | *** |
| Ambrosiella_hartigii - Raffaelea_amase | 2.275313 | 0.675935 | 40 | 3.366171 | 0.047792 | 4.7791533555e-02 | * |
| Ambrosiella_hartigii - Raffaelea_canadensis | -9.51695 | 0.675935 | 40 | -14.0797 | 4.68E-13 | 4.6807002718e-13 | *** |
| Ambrosiella_hartigii - Raffaelea_montetyi | -9.47711 | 0.675935 | 40 | -14.0207 | 4.69E-13 | 4.6851411639e-13 | *** |
| Ambrosiella_hartigii - Raffaelea_sulphurea | -6.39101 | 0.675935 | 40 | -9.45506 | 4.16E-10 | 4.1644399040e-10 | *** |
| Ambrosiella_roeperi - Fusarium_euwallaceae | -1.28857 | 0.675935 | 40 | -1.90635 | 0.664902 | 6.6490158886e-01 | |
| Ambrosiella_roeperi - Graphium_euwallaceae | -5.01196 | 0.675935 | 40 | -7.41485 | 2.14E-07 | 2.1425086860e-07 | *** |
| Ambrosiella_roeperi - Raffaelea_amase | 1.207483 | 0.675935 | 40 | 1.786389 | 0.739404 | 7.3940414101e-01 | |
| Ambrosiella_roeperi - Raffaelea_canadensis | -10.5848 | 0.675935 | 40 | -15.6595 | 4.64E-13 | 4.6407322429e-13 | *** |
| Ambrosiella_roeperi - Raffaelea_montetyi | -10.5449 | 0.675935 | 40 | -15.6005 | 4.64E-13 | 4.6407322429e-13 | *** |
| Ambrosiella_roeperi - Raffaelea_sulphurea | -7.45884 | 0.675935 | 40 | -11.0348 | 5.14E-12 | 5.1418869162e-12 | *** |
| Fusarium_euwallaceae - Graphium_euwallaceae | -3.72338 | 0.675935 | 40 | -5.50849 | 9.3E-05 | 9.2973528575e-05 | *** |
| Fusarium_euwallaceae - Raffaelea_amase | 2.496055 | 0.675935 | 40 | 3.692744 | 0.020669 | 2.0668637126e-02 | * |
| Fusarium_euwallaceae - Raffaelea_canadensis | -9.2962 | 0.675935 | 40 | -13.7531 | 4.73E-13 | 4.7273296389e-13 | *** |
| Fusarium_euwallaceae - Raffaelea_montetyi | -9.25637 | 0.675935 | 40 | -13.6942 | 4.75E-13 | 4.7450932072e-13 | *** |
| Fusarium_euwallaceae - Raffaelea_sulphurea | -6.17026 | 0.675935 | 40 | -9.12849 | 1.1E-09 | 1.0964806840e-09 | *** |
| Graphium_euwallaceae - Raffaelea_amase | 6.219439 | 0.675935 | 40 | 9.201237 | 8.83E-10 | 8.8274476617e-10 | *** |
| Graphium_euwallaceae - Raffaelea_canadensis | -5.57282 | 0.675935 | 40 | -8.24461 | 1.61E-08 | 1.6052070784e-08 | *** |
| Graphium_euwallaceae - Raffaelea_montetyi | -5.53298 | 0.675935 | 40 | -8.18567 | 1.93E-08 | 1.9255544270e-08 | *** |
| Graphium_euwallaceae - Raffaelea_sulphurea | -2.44688 | 0.675935 | 40 | -3.61999 | 0.025046 | 2.5046284412e-02 | * |
| Raffaelea_amase - Raffaelea_canadensis | -11.7923 | 0.675935 | 40 | -17.4458 | 4.64E-13 | 4.6362913508e-13 | *** |
| Raffaelea_amase - Raffaelea_montetyi | -11.7524 | 0.675935 | 40 | -17.3869 | 4.64E-13 | 4.6362913508e-13 | *** |
| Raffaelea_amase - Raffaelea_sulphurea | -8.66632 | 0.675935 | 40 | -12.8212 | 5.2E-13 | 5.2047255394e-13 | *** |
| Raffaelea_canadensis - Raffaelea_montetyi | 0.039836 | 0.675935 | 40 | 0.058935 | 1 | 1.0000000000e+00 | |
| Raffaelea_canadensis - Raffaelea_sulphurea | 3.125941 | 0.675935 | 40 | 4.624617 | 0.00144 | 1.4401300719e-03 | ** |
| Raffaelea_montetyi - Raffaelea_sulphurea | 3.086105 | 0.675935 | 40 | 4.565683 | 0.001719 | 1.7193341637e-03 | ** |

| Sample | emmean | SE | df | lower.CL | upper.CL | .group |
| --- | --- | --- | --- | --- | --- | --- |
| Raffaelea_amase | 5.33E-15 | 0.477958 | 40 | -1.41596 | 1.415964 | a |
| Ambrosiella_roeperi | 1.207483 | 0.477958 | 40 | -0.20848 | 2.623447 | ab |
| Ambrosiella_hartigii | 2.275313 | 0.477958 | 40 | 0.859349 | 3.691277 | bc |
| Fusarium_euwallaceae | 2.496055 | 0.477958 | 40 | 1.080091 | 3.912019 | bc |
| Ambrosiella_grosmanniae | 3.803733 | 0.477958 | 40 | 2.387769 | 5.219697 | c |
| Graphium_euwallaceae | 6.219439 | 0.477958 | 40 | 4.803475 | 7.635403 | d |
| Raffaelea_sulphurea | 8.666319 | 0.477958 | 40 | 7.250354 | 10.08228 | e |
| Alloascoidea_hylecoeti | 9.902437 | 0.477958 | 40 | 8.486472 | 11.3184 | ef |
| Raffaelea_montetyi | 11.75242 | 0.477958 | 40 | 10.33646 | 13.16839 | f |
| Raffaelea_canadensis | 11.79226 | 0.477958 | 40 | 10.3763 | 13.20822 | f |

Bark beetle fungi

Modell

Sqrt_Normal

| Resid. Df | Resid. Dev | Df | Deviance | Pr(>Chi) | p.value_formatted | Significance |
| --- | --- | --- | --- | --- | --- | --- |
| 54 | 1134.976 |  |  |  | NA |  |
| 44 | 39.82412 | 10 | 1095.152 | 1E-253 | 1.0097236474e-253 | *** |

| contrast | estimate | SE | df | t.ratio | p.value | p.value_formatted | Significance |
| --- | --- | --- | --- | --- | --- | --- | --- |
| Ceratocystiopsis_brevicomi - Ceratocystiopsis_ranaculosus | -6.80217 | 0.601696 | 44 | -11.305 | 1.59E-12 | 1.5948353749e-12 | *** |
| Ceratocystiopsis_brevicomi - Endoconidiophora_polonica | -2.42949 | 0.601696 | 44 | -4.03774 | 0.008703 | 8.7032267539e-03 | ** |
| Ceratocystiopsis_brevicomi - Grosmannia_clavigera | -11.6908 | 0.601696 | 44 | -19.4298 | 8.25E-13 | 8.2456264039e-13 | *** |
| Ceratocystiopsis_brevicomi - Grosmannia_penicillata | -17.2262 | 0.601696 | 44 | -28.6294 | 8.25E-13 | 8.2456264039e-13 | *** |
| Ceratocystiopsis_brevicomi - Leptographium_abietinum | -11.8559 | 0.601696 | 44 | -19.7041 | 8.25E-13 | 8.2456264039e-13 | *** |
| Ceratocystiopsis_brevicomi - Leptographium_longiclavatum | -8.23084 | 0.601696 | 44 | -13.6794 | 8.31E-13 | 8.3144602314e-13 | *** |
| Ceratocystiopsis_brevicomi - Ophiostoma_bicolor | -8.14458 | 0.601696 | 44 | -13.536 | 8.31E-13 | 8.3144602314e-13 | *** |
| Ceratocystiopsis_brevicomi - Ophiostoma_ips | -9.78749 | 0.601696 | 44 | -16.2665 | 8.25E-13 | 8.2478468499e-13 | *** |
| Ceratocystiopsis_brevicomi - Ophiostoma_montium | -10.2676 | 0.601696 | 44 | -17.0645 | 8.25E-13 | 8.2456264039e-13 | *** |
| Ceratocystiopsis_brevicomi - Trichosporium_symbioticum | -6.37397 | 0.601696 | 44 | -10.5933 | 6.83E-12 | 6.8335337389e-12 | *** |
| Ceratocystiopsis_ranaculosus - Endoconidiophora_polonica | 4.372677 | 0.601696 | 44 | 7.267259 | 2.44E-07 | 2.4444794178e-07 | *** |
| Ceratocystiopsis_ranaculosus - Grosmannia_clavigera | -4.88867 | 0.601696 | 44 | -8.12482 | 1.42E-08 | 1.4238519142e-08 | *** |
| Ceratocystiopsis_ranaculosus - Grosmannia_penicillata | -10.424 | 0.601696 | 44 | -17.3244 | 8.25E-13 | 8.2456264039e-13 | *** |
| Ceratocystiopsis_ranaculosus - Leptographium_abietinum | -5.05371 | 0.601696 | 44 | -8.39912 | 5.8E-09 | 5.8046666362e-09 | *** |
| Ceratocystiopsis_ranaculosus - Leptographium_longiclavatum | -1.42867 | 0.601696 | 44 | -2.37441 | 0.405312 | 4.0531235353e-01 | |
| Ceratocystiopsis_ranaculosus - Ophiostoma_bicolor | -1.34241 | 0.601696 | 44 | -2.23104 | 0.496403 | 4.9640293735e-01 | |
| Ceratocystiopsis_ranaculosus - Ophiostoma_ips | -2.98532 | 0.601696 | 44 | -4.96152 | 0.000515 | 5.1533769865e-04 | *** |
| Ceratocystiopsis_ranaculosus - Ophiostoma_montium | -3.46547 | 0.601696 | 44 | -5.7595 | 3.8E-05 | 3.8027396833e-05 | *** |
| Ceratocystiopsis_ranaculosus - Trichosporium_symbioticum | 0.428199 | 0.601696 | 44 | 0.711654 | 0.999694 | 9.9969440665e-01 | |
| Endoconidiophora_polonica - Grosmannia_clavigera | -9.26134 | 0.601696 | 44 | -15.3921 | 8.25E-13 | 8.2478468499e-13 | *** |
| Endoconidiophora_polonica - Grosmannia_penicillata | -14.7967 | 0.601696 | 44 | -24.5916 | 8.25E-13 | 8.2456264039e-13 | *** |
| Endoconidiophora_polonica - Leptographium_abietinum | -9.42639 | 0.601696 | 44 | -15.6664 | 8.25E-13 | 8.2478468499e-13 | *** |
| Endoconidiophora_polonica - Leptographium_longiclavatum | -5.80135 | 0.601696 | 44 | -9.64167 | 1.11E-10 | 1.1064016370e-10 | *** |
| Endoconidiophora_polonica - Ophiostoma_bicolor | -5.71509 | 0.601696 | 44 | -9.4983 | 1.73E-10 | 1.7283652287e-10 | *** |
| Endoconidiophora_polonica - Ophiostoma_ips | -7.358 | 0.601696 | 44 | -12.2288 | 9E-13 | 9.0039087297e-13 | *** |
| Endoconidiophora_polonica - Ophiostoma_montium | -7.83814 | 0.601696 | 44 | -13.0268 | 8.39E-13 | 8.3932860662e-13 | *** |
| Endoconidiophora_polonica - Trichosporium_symbioticum | -3.94448 | 0.601696 | 44 | -6.55561 | 2.66E-06 | 2.6553585718e-06 | *** |
| Grosmannia_clavigera - Grosmannia_penicillata | -5.53532 | 0.601696 | 44 | -9.19954 | 4.43E-10 | 4.4252923548e-10 | *** |
| Grosmannia_clavigera - Leptographium_abietinum | -0.16505 | 0.601696 | 44 | -0.2743 | 1 | 9.9999996344e-01 | |
| Grosmannia_clavigera - Leptographium_longiclavatum | 3.459994 | 0.601696 | 44 | 5.750406 | 3.92E-05 | 3.9192703019e-05 | *** |
| Grosmannia_clavigera - Ophiostoma_bicolor | 3.546259 | 0.601696 | 44 | 5.893777 | 2.43E-05 | 2.4333173624e-05 | *** |
| Grosmannia_clavigera - Ophiostoma_ips | 1.903344 | 0.601696 | 44 | 3.163302 | 0.087221 | 8.7221293307e-02 | . |
| Grosmannia_clavigera - Ophiostoma_montium | 1.423201 | 0.601696 | 44 | 2.365318 | 0.41088 | 4.1088022292e-01 | |
| Grosmannia_clavigera - Trichosporium_symbioticum | 5.316866 | 0.601696 | 44 | 8.836473 | 1.41E-09 | 1.4096021017e-09 | *** |
| Grosmannia_penicillata - Leptographium_abietinum | 5.370275 | 0.601696 | 44 | 8.925238 | 1.06E-09 | 1.0603370404e-09 | *** |
| Grosmannia_penicillata - Leptographium_longiclavatum | 8.995314 | 0.601696 | 44 | 14.94994 | 8.26E-13 | 8.2556184111e-13 | *** |
| Grosmannia_penicillata - Ophiostoma_bicolor | 9.08158 | 0.601696 | 44 | 15.09331 | 8.25E-13 | 8.2511775190e-13 | *** |
| Grosmannia_penicillata - Ophiostoma_ips | 7.438665 | 0.601696 | 44 | 12.36284 | 8.78E-13 | 8.7840845708e-13 | *** |
| Grosmannia_penicillata - Ophiostoma_montium | 6.958522 | 0.601696 | 44 | 11.56486 | 1.2E-12 | 1.2033707364e-12 | *** |
| Grosmannia_penicillata - Trichosporium_symbioticum | 10.85219 | 0.601696 | 44 | 18.03601 | 8.25E-13 | 8.2456264039e-13 | *** |
| Leptographium_abietinum - Leptographium_longiclavatum | 3.625039 | 0.601696 | 44 | 6.024706 | 1.57E-05 | 1.5723914770e-05 | *** |
| Leptographium_abietinum - Ophiostoma_bicolor | 3.711304 | 0.601696 | 44 | 6.168077 | 9.74E-06 | 9.7362957011e-06 | *** |
| Leptographium_abietinum - Ophiostoma_ips | 2.06839 | 0.601696 | 44 | 3.437602 | 0.044673 | 4.4673111103e-02 | * |
| Leptographium_abietinum - Ophiostoma_montium | 1.588246 | 0.601696 | 44 | 2.639618 | 0.26049 | 2.6049030345e-01 | |
| Leptographium_abietinum - Trichosporium_symbioticum | 5.481911 | 0.601696 | 44 | 9.110773 | 5.87E-10 | 5.8653037982e-10 | *** |
| Leptographium_longiclavatum - Ophiostoma_bicolor | 0.086265 | 0.601696 | 44 | 0.143371 | 1 | 9.9999999994e-01 | |
| Leptographium_longiclavatum - Ophiostoma_ips | -1.55665 | 0.601696 | 44 | -2.5871 | 0.286197 | 2.8619707774e-01 | |
| Leptographium_longiclavatum - Ophiostoma_montium | -2.03679 | 0.601696 | 44 | -3.38509 | 0.051 | 5.1000265657e-02 | . |
| Leptographium_longiclavatum - Trichosporium_symbioticum | 1.856873 | 0.601696 | 44 | 3.086067 | 0.104167 | 1.0416711621e-01 | |
| Ophiostoma_bicolor - Ophiostoma_ips | -1.64291 | 0.601696 | 44 | -2.73048 | 0.219749 | 2.1974906769e-01 | |
| Ophiostoma_bicolor - Ophiostoma_montium | -2.12306 | 0.601696 | 44 | -3.52846 | 0.035363 | 3.5363435699e-02 | * |
| Ophiostoma_bicolor - Trichosporium_symbioticum | 1.770607 | 0.601696 | 44 | 2.942696 | 0.142823 | 1.4282310834e-01 | |
| Ophiostoma_ips - Ophiostoma_montium | -0.48014 | 0.601696 | 44 | -0.79798 | 0.999171 | 9.9917086493e-01 | |
| Ophiostoma_ips - Trichosporium_symbioticum | 3.413522 | 0.601696 | 44 | 5.673171 | 5.06E-05 | 5.0627173389e-05 | *** |
| Ophiostoma_montium - Trichosporium_symbioticum | 3.893665 | 0.601696 | 44 | 6.471155 | 3.53E-06 | 3.5253711376e-06 | *** |

| Sample | emmean | SE | df | lower.CL | upper.CL | .group |
| --- | --- | --- | --- | --- | --- | --- |
| Ceratocystiopsis_brevicomi | 2.75E-15 | 0.425463 | 44 | -1.26882 | 1.268817 | a |
| Endoconidiophora_polonica | 2.429492 | 0.425463 | 44 | 1.160675 | 3.698309 | b |
| Trichosporium_symbioticum | 6.37397 | 0.425463 | 44 | 5.105153 | 7.642788 | c |
| Ceratocystiopsis_ranaculosus | 6.802169 | 0.425463 | 44 | 5.533352 | 8.070987 | c |
| Ophiostoma_bicolor | 8.144577 | 0.425463 | 44 | 6.87576 | 9.413395 | cd |
| Leptographium_longiclavatum | 8.230843 | 0.425463 | 44 | 6.962026 | 9.49966 | cde |
| Ophiostoma_ips | 9.787492 | 0.425463 | 44 | 8.518675 | 11.05631 | def |
| Ophiostoma_montium | 10.26764 | 0.425463 | 44 | 8.998818 | 11.53645 | efg |
| Grosmannia_clavigera | 11.69084 | 0.425463 | 44 | 10.42202 | 12.95965 | fg |
| Leptographium_abietinum | 11.85588 | 0.425463 | 44 | 10.58706 | 13.1247 | g |
| Grosmannia_penicillata | 17.22616 | 0.425463 | 44 | 15.95734 | 18.49497 | h |

Others

Modell

Sqrt_Normal

| Resid. Df | Resid. Dev | Df | Deviance | Pr(>Chi) | p.value_formatted | Significance |
| --- | --- | --- | --- | --- | --- | --- |
| 44 | 672.9037 |  |  |  | NA |  |
| 36 | 32.08356 | 8 | 640.8202 | 5.7E-150 | 5.6756365787e-150 | *** |

| contrast | estimate | SE | df | t.ratio | p.value | p.value_formatted | Significance |
| --- | --- | --- | --- | --- | --- | --- | --- |
| Aspergillus_flavus - Beauveria_bassiana | 3.28E-15 | 0.597063 | 36 | 5.49E-15 | 1 | 1.0000000000e+00 | |
| Aspergillus_flavus - Chaetomium_globosum | 5.57E-15 | 0.597063 | 36 | 9.33E-15 | 1 | 1.0000000000e+00 | |
| Aspergillus_flavus - Esteya_vermicola | -2.62409 | 0.597063 | 36 | -4.39499 | 0.002708 | 2.7079346566e-03 | ** |
| Aspergillus_flavus - Ophiostoma_minus | -8.7557 | 0.597063 | 36 | -14.6646 | 0 | 0.0000000000e+00 | *** |
| Aspergillus_flavus - Ophiostoma_quercus | -7.53442 | 0.597063 | 36 | -12.6191 | 6.2E-14 | 6.1950444774e-14 | *** |
| Aspergillus_flavus - Paracremonium_pembeum | 1.43E-15 | 0.597063 | 36 | 2.4E-15 | 1 | 1.0000000000e+00 | |
| Aspergillus_flavus - Penicillium_commune | 2.92E-15 | 0.597063 | 36 | 4.89E-15 | 1 | 1.0000000000e+00 | |
| Aspergillus_flavus - Sporothrix_stenoceras | -8.41718 | 0.597063 | 36 | -14.0977 | 0 | 0.0000000000e+00 | *** |
| Beauveria_bassiana - Chaetomium_globosum | 2.3E-15 | 0.597063 | 36 | 3.85E-15 | 1 | 1.0000000000e+00 | |
| Beauveria_bassiana - Esteya_vermicola | -2.62409 | 0.597063 | 36 | -4.39499 | 0.002708 | 2.7079346566e-03 | ** |
| Beauveria_bassiana - Ophiostoma_minus | -8.7557 | 0.597063 | 36 | -14.6646 | 0 | 0.0000000000e+00 | *** |
| Beauveria_bassiana - Ophiostoma_quercus | -7.53442 | 0.597063 | 36 | -12.6191 | 6.2E-14 | 6.1950444774e-14 | *** |
| Beauveria_bassiana - Paracremonium_pembeum | -1.8E-15 | 0.597063 | 36 | -3.1E-15 | 1 | 1.0000000000e+00 | |
| Beauveria_bassiana - Penicillium_commune | -3.6E-16 | 0.597063 | 36 | -6E-16 | 1 | 1.0000000000e+00 | |
| Beauveria_bassiana - Sporothrix_stenoceras | -8.41718 | 0.597063 | 36 | -14.0977 | 0 | 0.0000000000e+00 | *** |
| Chaetomium_globosum - Esteya_vermicola | -2.62409 | 0.597063 | 36 | -4.39499 | 0.002708 | 2.7079346566e-03 | ** |
| Chaetomium_globosum - Ophiostoma_minus | -8.7557 | 0.597063 | 36 | -14.6646 | 0 | 0.0000000000e+00 | *** |
| Chaetomium_globosum - Ophiostoma_quercus | -7.53442 | 0.597063 | 36 | -12.6191 | 6.2E-14 | 6.1950444774e-14 | *** |
| Chaetomium_globosum - Paracremonium_pembeum | -4.1E-15 | 0.597063 | 36 | -6.9E-15 | 1 | 1.0000000000e+00 | |
| Chaetomium_globosum - Penicillium_commune | -2.7E-15 | 0.597063 | 36 | -4.4E-15 | 1 | 1.0000000000e+00 | |
| Chaetomium_globosum - Sporothrix_stenoceras | -8.41718 | 0.597063 | 36 | -14.0977 | 0 | 0.0000000000e+00 | *** |
| Esteya_vermicola - Ophiostoma_minus | -6.13161 | 0.597063 | 36 | -10.2696 | 1.06E-10 | 1.0631362457e-10 | *** |
| Esteya_vermicola - Ophiostoma_quercus | -4.91033 | 0.597063 | 36 | -8.22414 | 3.02E-08 | 3.0193051614e-08 | *** |
| Esteya_vermicola - Paracremonium_pembeum | 2.624088 | 0.597063 | 36 | 4.394995 | 0.002708 | 2.7079346566e-03 | ** |
| Esteya_vermicola - Penicillium_commune | 2.624088 | 0.597063 | 36 | 4.394995 | 0.002708 | 2.7079346566e-03 | ** |
| Esteya_vermicola - Sporothrix_stenoceras | -5.7931 | 0.597063 | 36 | -9.70266 | 4.83E-10 | 4.8323145485e-10 | *** |
| Ophiostoma_minus - Ophiostoma_quercus | 1.221286 | 0.597063 | 36 | 2.045489 | 0.523841 | 5.2384147115e-01 | |
| Ophiostoma_minus - Paracremonium_pembeum | 8.755702 | 0.597063 | 36 | 14.66462 | 0 | 0.0000000000e+00 | *** |
| Ophiostoma_minus - Penicillium_commune | 8.755702 | 0.597063 | 36 | 14.66462 | 0 | 0.0000000000e+00 | *** |
| Ophiostoma_minus - Sporothrix_stenoceras | 0.338519 | 0.597063 | 36 | 0.566973 | 0.999671 | 9.9967116656e-01 | |
| Ophiostoma_quercus - Paracremonium_pembeum | 7.534417 | 0.597063 | 36 | 12.61914 | 6.2E-14 | 6.1950444774e-14 | *** |
| Ophiostoma_quercus - Penicillium_commune | 7.534417 | 0.597063 | 36 | 12.61914 | 6.2E-14 | 6.1950444774e-14 | *** |
| Ophiostoma_quercus - Sporothrix_stenoceras | -0.88277 | 0.597063 | 36 | -1.47852 | 0.857936 | 8.5793639435e-01 | |
| Paracremonium_pembeum - Penicillium_commune | 1.49E-15 | 0.597063 | 36 | 2.49E-15 | 1 | 1.0000000000e+00 | |
| Paracremonium_pembeum - Sporothrix_stenoceras | -8.41718 | 0.597063 | 36 | -14.0977 | 0 | 0.0000000000e+00 | *** |
| Penicillium_commune - Sporothrix_stenoceras | -8.41718 | 0.597063 | 36 | -14.0977 | 0 | 0.0000000000e+00 | *** |

| Sample | emmean | SE | df | lower.CL | upper.CL | .group |
| --- | --- | --- | --- | --- | --- | --- |
| Chaetomium_globosum | -2E-15 | 0.422187 | 36 | -1.24179 | 1.241785 | a |
| Beauveria_bassiana | 2.55E-16 | 0.422187 | 36 | -1.24179 | 1.241785 | a |
| Penicillium_commune | 6.13E-16 | 0.422187 | 36 | -1.24179 | 1.241785 | a |
| Paracremonium_pembeum | 2.1E-15 | 0.422187 | 36 | -1.24179 | 1.241785 | a |
| Aspergillus_flavus | 3.53E-15 | 0.422187 | 36 | -1.24179 | 1.241785 | a |
| Esteya_vermicola | 2.624088 | 0.422187 | 36 | 1.382303 | 3.865873 | b |
| Ophiostoma_quercus | 7.534417 | 0.422187 | 36 | 6.292631 | 8.776202 | c |
| Sporothrix_stenoceras | 8.417184 | 0.422187 | 36 | 7.175398 | 9.658969 | c |
| Ophiostoma_minus | 8.755702 | 0.422187 | 36 | 7.513917 | 9.997488 | c |

**Ethanol**

Ambrosia beetle fungi

**Modell**

Sqrt_Normal

| **Resid. Df** | **Resid. Dev** | **Df** | **Deviance** | **Pr(>Chi)** | **p.value_formatted** | **Significance** |
| --- | --- | --- | --- | --- | --- | --- |
| 49 | 539.7347 |  |  |  | NA |  |
| 40 | 51.28879 | 9 | 488.4459 | 1.59E-76 | 1.5928125115e-76 | *** |

| **contrast** | **estimate** | **SE** | **df** | **t.ratio** | **p.value** | **p.value_formatted** | **Significance** |
| --- | --- | --- | --- | --- | --- | --- | --- |
| Alloascoidea_hylecoeti - Ambrosiella_grosmanniae | 2.985741 | 0.716162 | 40 | 4.169087 | 0.005516 | 5.5164751253e-03 | ** |
| Alloascoidea_hylecoeti - Ambrosiella_hartigii | 2.856529 | 0.716162 | 40 | 3.988663 | 0.009205 | 9.2053775780e-03 | ** |
| Alloascoidea_hylecoeti - Ambrosiella_roeperi | 3.163501 | 0.716162 | 40 | 4.417298 | 0.002675 | 2.6748010607e-03 | ** |
| Alloascoidea_hylecoeti - Fusarium_euwallaceae | 2.533931 | 0.716162 | 40 | 3.53821 | 0.030974 | 3.0974068389e-02 | * |
| Alloascoidea_hylecoeti - Graphium_euwallaceae | 4.663963 | 0.716162 | 40 | 6.512442 | 3.78E-06 | 3.7831291819e-06 | *** |
| Alloascoidea_hylecoeti - Raffaelea_amase | 4.663963 | 0.716162 | 40 | 6.512442 | 3.78E-06 | 3.7831291819e-06 | *** |
| Alloascoidea_hylecoeti - Raffaelea_canadensis | -5.22301 | 0.716162 | 40 | -7.29305 | 3.15E-07 | 3.1487774899e-07 | *** |
| Alloascoidea_hylecoeti - Raffaelea_montetyi | -3.145 | 0.716162 | 40 | -4.39147 | 0.002887 | 2.8867800415e-03 | ** |
| Alloascoidea_hylecoeti - Raffaelea_sulphurea | 2.372234 | 0.716162 | 40 | 3.312427 | 0.054511 | 5.4511424343e-02 | . |
| Ambrosiella_grosmanniae - Ambrosiella_hartigii | -0.12921 | 0.716162 | 40 | -0.18042 | 1 | 9.9999999482e-01 | |
| Ambrosiella_grosmanniae - Ambrosiella_roeperi | 0.177759 | 0.716162 | 40 | 0.248211 | 1 | 9.9999991140e-01 | |
| Ambrosiella_grosmanniae - Fusarium_euwallaceae | -0.45181 | 0.716162 | 40 | -0.63088 | 0.999726 | 9.9972644000e-01 | |
| Ambrosiella_grosmanniae - Graphium_euwallaceae | 1.678222 | 0.716162 | 40 | 2.343355 | 0.385269 | 3.8526928990e-01 | |
| Ambrosiella_grosmanniae - Raffaelea_amase | 1.678222 | 0.716162 | 40 | 2.343355 | 0.385269 | 3.8526928990e-01 | |
| Ambrosiella_grosmanniae - Raffaelea_canadensis | -8.20875 | 0.716162 | 40 | -11.4621 | 1.96E-12 | 1.9551027464e-12 | *** |
| Ambrosiella_grosmanniae - Raffaelea_montetyi | -6.13074 | 0.716162 | 40 | -8.56056 | 6.09E-09 | 6.0891416354e-09 | *** |
| Ambrosiella_grosmanniae - Raffaelea_sulphurea | -0.61351 | 0.716162 | 40 | -0.85666 | 0.99696 | 9.9695997886e-01 | |
| Ambrosiella_hartigii - Ambrosiella_roeperi | 0.306972 | 0.716162 | 40 | 0.428635 | 0.999989 | 9.9998940102e-01 | |
| Ambrosiella_hartigii - Fusarium_euwallaceae | -0.3226 | 0.716162 | 40 | -0.45045 | 0.999984 | 9.9998376830e-01 | |
| Ambrosiella_hartigii - Graphium_euwallaceae | 1.807435 | 0.716162 | 40 | 2.523779 | 0.287216 | 2.8721552226e-01 | |
| Ambrosiella_hartigii - Raffaelea_amase | 1.807435 | 0.716162 | 40 | 2.523779 | 0.287216 | 2.8721552226e-01 | |
| Ambrosiella_hartigii - Raffaelea_canadensis | -8.07954 | 0.716162 | 40 | -11.2817 | 2.87E-12 | 2.8659297158e-12 | *** |
| Ambrosiella_hartigii - Raffaelea_montetyi | -6.00153 | 0.716162 | 40 | -8.38013 | 1.06E-08 | 1.0577832832e-08 | *** |
| Ambrosiella_hartigii - Raffaelea_sulphurea | -0.48429 | 0.716162 | 40 | -0.67624 | 0.99952 | 9.9951969419e-01 | |
| Ambrosiella_roeperi - Fusarium_euwallaceae | -0.62957 | 0.716162 | 40 | -0.87909 | 0.996311 | 9.9631107258e-01 | |
| Ambrosiella_roeperi - Graphium_euwallaceae | 1.500463 | 0.716162 | 40 | 2.095144 | 0.541311 | 5.4131139133e-01 | |
| Ambrosiella_roeperi - Raffaelea_amase | 1.500463 | 0.716162 | 40 | 2.095144 | 0.541311 | 5.4131139133e-01 | |
| Ambrosiella_roeperi - Raffaelea_canadensis | -8.38651 | 0.716162 | 40 | -11.7104 | 1.24E-12 | 1.2434497876e-12 | *** |
| Ambrosiella_roeperi - Raffaelea_montetyi | -6.3085 | 0.716162 | 40 | -8.80877 | 2.87E-09 | 2.8651938599e-09 | *** |
| Ambrosiella_roeperi - Raffaelea_sulphurea | -0.79127 | 0.716162 | 40 | -1.10487 | 0.981363 | 9.8136339626e-01 | |
| Fusarium_euwallaceae - Graphium_euwallaceae | 2.130032 | 0.716162 | 40 | 2.974233 | 0.118835 | 1.1883484187e-01 | |
| Fusarium_euwallaceae - Raffaelea_amase | 2.130032 | 0.716162 | 40 | 2.974233 | 0.118835 | 1.1883484187e-01 | |
| Fusarium_euwallaceae - Raffaelea_canadensis | -7.75694 | 0.716162 | 40 | -10.8313 | 8.64E-12 | 8.6401996668e-12 | *** |
| Fusarium_euwallaceae - Raffaelea_montetyi | -5.67893 | 0.716162 | 40 | -7.92968 | 4.26E-08 | 4.2611917883e-08 | *** |
| Fusarium_euwallaceae - Raffaelea_sulphurea | -0.1617 | 0.716162 | 40 | -0.22578 | 1 | 9.9999996178e-01 | |
| Graphium_euwallaceae - Raffaelea_amase | -5.3E-15 | 0.716162 | 40 | -7.4E-15 | 1 | 1.0000000000e+00 | |
| Graphium_euwallaceae - Raffaelea_canadensis | -9.88697 | 0.716162 | 40 | -13.8055 | 4.72E-13 | 4.7162274086e-13 | *** |
| Graphium_euwallaceae - Raffaelea_montetyi | -7.80897 | 0.716162 | 40 | -10.9039 | 7.16E-12 | 7.1573857952e-12 | *** |
| Graphium_euwallaceae - Raffaelea_sulphurea | -2.29173 | 0.716162 | 40 | -3.20002 | 0.071313 | 7.1313259322e-02 | . |
| Raffaelea_amase - Raffaelea_canadensis | -9.88697 | 0.716162 | 40 | -13.8055 | 4.72E-13 | 4.7162274086e-13 | *** |
| Raffaelea_amase - Raffaelea_montetyi | -7.80897 | 0.716162 | 40 | -10.9039 | 7.16E-12 | 7.1573857952e-12 | *** |
| Raffaelea_amase - Raffaelea_sulphurea | -2.29173 | 0.716162 | 40 | -3.20002 | 0.071313 | 7.1313259322e-02 | . |
| Raffaelea_canadensis - Raffaelea_montetyi | 2.078003 | 0.716162 | 40 | 2.901583 | 0.138805 | 1.3880548493e-01 | |
| Raffaelea_canadensis - Raffaelea_sulphurea | 7.595241 | 0.716162 | 40 | 10.60548 | 1.58E-11 | 1.5753176541e-11 | *** |
| Raffaelea_montetyi - Raffaelea_sulphurea | 5.517237 | 0.716162 | 40 | 7.703896 | 8.63E-08 | 8.6290618873e-08 | *** |

| **Sample** | **emmean** | **SE** | **df** | **lower.CL** | **upper.CL** | **.group** |
| --- | --- | --- | --- | --- | --- | --- |
| Graphium_euwallaceae | -3.6E-15 | 0.506403 | 40 | -1.50023 | 1.500232 | a |
| Raffaelea_amase | 1.78E-15 | 0.506403 | 40 | -1.50023 | 1.500232 | a |
| Ambrosiella_roeperi | 1.500463 | 0.506403 | 40 | 0.00023 | 3.000695 | a |
| Ambrosiella_grosmanniae | 1.678222 | 0.506403 | 40 | 0.17799 | 3.178454 | a |
| Ambrosiella_hartigii | 1.807435 | 0.506403 | 40 | 0.307202 | 3.307667 | a |
| Fusarium_euwallaceae | 2.130032 | 0.506403 | 40 | 0.6298 | 3.630265 | a |
| Raffaelea_sulphurea | 2.291729 | 0.506403 | 40 | 0.791497 | 3.791961 | ab |
| Alloascoidea_hylecoeti | 4.663963 | 0.506403 | 40 | 3.163731 | 6.164196 | b |
| Raffaelea_montetyi | 7.808966 | 0.506403 | 40 | 6.308734 | 9.309199 | c |
| Raffaelea_canadensis | 9.88697 | 0.506403 | 40 | 8.386737 | 11.3872 | c |

Bark beetle fungi

Modell

Sqrt_Normal

| Resid. Df | Resid. Dev | Df | Deviance | Pr(>Chi) | p.value_formatted | Significance |
| --- | --- | --- | --- | --- | --- | --- |
| 54 | 1416.944 |  |  |  | NA |  |
| 44 | 55.37829 | 10 | 1361.565 | 4.4E-226 | 4.3962557264e-226 | *** |

| contrast | estimate | SE | df | t.ratio | p.value | p.value_formatted | Significance |
| --- | --- | --- | --- | --- | --- | --- | --- |
| Ceratocystiopsis_brevicomi - Ceratocystiopsis_ranaculosus | -10.681 | 0.709534 | 44 | -15.0535 | 8.25E-13 | 8.2522877420e-13 | *** |
| Ceratocystiopsis_brevicomi - Endoconidiophora_polonica | 9E-15 | 0.709534 | 44 | 1.27E-14 | 1 | 1.0000000000e+00 | |
| Ceratocystiopsis_brevicomi - Grosmannia_clavigera | -13.8422 | 0.709534 | 44 | -19.5089 | 8.25E-13 | 8.2456264039e-13 | *** |
| Ceratocystiopsis_brevicomi - Grosmannia_penicillata | -15.062 | 0.709534 | 44 | -21.228 | 8.25E-13 | 8.2456264039e-13 | *** |
| Ceratocystiopsis_brevicomi - Leptographium_abietinum | -11.5724 | 0.709534 | 44 | -16.3099 | 8.25E-13 | 8.2478468499e-13 | *** |
| Ceratocystiopsis_brevicomi - Leptographium_longiclavatum | -10.3068 | 0.709534 | 44 | -14.5261 | 8.26E-13 | 8.2611695262e-13 | *** |
| Ceratocystiopsis_brevicomi - Ophiostoma_bicolor | -6.25689 | 0.709534 | 44 | -8.8183 | 1.49E-09 | 1.4943782878e-09 | *** |
| Ceratocystiopsis_brevicomi - Ophiostoma_ips | -9.1078 | 0.709534 | 44 | -12.8363 | 8.48E-13 | 8.4821039081e-13 | *** |
| Ceratocystiopsis_brevicomi - Ophiostoma_montium | -9.55348 | 0.709534 | 44 | -13.4644 | 8.32E-13 | 8.3200113465e-13 | *** |
| Ceratocystiopsis_brevicomi - Trichosporium_symbioticum | -2.66524 | 0.709534 | 44 | -3.75632 | 0.019228 | 1.9227896031e-02 | * |
| Ceratocystiopsis_ranaculosus - Endoconidiophora_polonica | 10.681 | 0.709534 | 44 | 15.05353 | 8.25E-13 | 8.2522877420e-13 | *** |
| Ceratocystiopsis_ranaculosus - Grosmannia_clavigera | -3.16123 | 0.709534 | 44 | -4.45536 | 0.002513 | 2.5128133866e-03 | ** |
| Ceratocystiopsis_ranaculosus - Grosmannia_penicillata | -4.38098 | 0.709534 | 44 | -6.17445 | 9.53E-06 | 9.5308188796e-06 | *** |
| Ceratocystiopsis_ranaculosus - Leptographium_abietinum | -0.89141 | 0.709534 | 44 | -1.25633 | 0.971381 | 9.7138070229e-01 | |
| Ceratocystiopsis_ranaculosus - Leptographium_longiclavatum | 0.374214 | 0.709534 | 44 | 0.527407 | 0.99998 | 9.9998013573e-01 | |
| Ceratocystiopsis_ranaculosus - Ophiostoma_bicolor | 4.424109 | 0.709534 | 44 | 6.235228 | 7.78E-06 | 7.7758763437e-06 | *** |
| Ceratocystiopsis_ranaculosus - Ophiostoma_ips | 1.573199 | 0.709534 | 44 | 2.217227 | 0.50548 | 5.0547986504e-01 | |
| Ceratocystiopsis_ranaculosus - Ophiostoma_montium | 1.127513 | 0.709534 | 44 | 1.589089 | 0.878424 | 8.7842359522e-01 | |
| Ceratocystiopsis_ranaculosus - Trichosporium_symbioticum | 8.015757 | 0.709534 | 44 | 11.29721 | 1.61E-12 | 1.6109336087e-12 | *** |
| Endoconidiophora_polonica - Grosmannia_clavigera | -13.8422 | 0.709534 | 44 | -19.5089 | 8.25E-13 | 8.2456264039e-13 | *** |
| Endoconidiophora_polonica - Grosmannia_penicillata | -15.062 | 0.709534 | 44 | -21.228 | 8.25E-13 | 8.2456264039e-13 | *** |
| Endoconidiophora_polonica - Leptographium_abietinum | -11.5724 | 0.709534 | 44 | -16.3099 | 8.25E-13 | 8.2478468499e-13 | *** |
| Endoconidiophora_polonica - Leptographium_longiclavatum | -10.3068 | 0.709534 | 44 | -14.5261 | 8.26E-13 | 8.2611695262e-13 | *** |
| Endoconidiophora_polonica - Ophiostoma_bicolor | -6.25689 | 0.709534 | 44 | -8.8183 | 1.49E-09 | 1.4943782878e-09 | *** |
| Endoconidiophora_polonica - Ophiostoma_ips | -9.1078 | 0.709534 | 44 | -12.8363 | 8.48E-13 | 8.4821039081e-13 | *** |
| Endoconidiophora_polonica - Ophiostoma_montium | -9.55348 | 0.709534 | 44 | -13.4644 | 8.32E-13 | 8.3200113465e-13 | *** |
| Endoconidiophora_polonica - Trichosporium_symbioticum | -2.66524 | 0.709534 | 44 | -3.75632 | 0.019228 | 1.9227896031e-02 | * |
| Grosmannia_clavigera - Grosmannia_penicillata | -1.21976 | 0.709534 | 44 | -1.71909 | 0.817734 | 8.1773392052e-01 | |
| Grosmannia_clavigera - Leptographium_abietinum | 2.269817 | 0.709534 | 44 | 3.199023 | 0.080209 | 8.0209432610e-02 | . |
| Grosmannia_clavigera - Leptographium_longiclavatum | 3.535443 | 0.709534 | 44 | 4.982765 | 0.000481 | 4.8147848922e-04 | *** |
| Grosmannia_clavigera - Ophiostoma_bicolor | 7.585338 | 0.709534 | 44 | 10.69059 | 5.32E-12 | 5.3247406484e-12 | *** |
| Grosmannia_clavigera - Ophiostoma_ips | 4.734428 | 0.709534 | 44 | 6.672585 | 1.79E-06 | 1.7930957799e-06 | *** |
| Grosmannia_clavigera - Ophiostoma_montium | 4.288743 | 0.709534 | 44 | 6.044447 | 1.47E-05 | 1.4720659006e-05 | *** |
| Grosmannia_clavigera - Trichosporium_symbioticum | 11.17699 | 0.709534 | 44 | 15.75256 | 8.25E-13 | 8.2478468499e-13 | *** |
| Grosmannia_penicillata - Leptographium_abietinum | 3.489572 | 0.709534 | 44 | 4.918115 | 0.000592 | 5.9189477236e-04 | *** |
| Grosmannia_penicillata - Leptographium_longiclavatum | 4.755198 | 0.709534 | 44 | 6.701857 | 1.63E-06 | 1.6253014244e-06 | *** |
| Grosmannia_penicillata - Ophiostoma_bicolor | 8.805093 | 0.709534 | 44 | 12.40968 | 8.73E-13 | 8.7319040887e-13 | *** |
| Grosmannia_penicillata - Ophiostoma_ips | 5.954183 | 0.709534 | 44 | 8.391677 | 5.95E-09 | 5.9471683134e-09 | *** |
| Grosmannia_penicillata - Ophiostoma_montium | 5.508498 | 0.709534 | 44 | 7.763539 | 4.69E-08 | 4.6877272175e-08 | *** |
| Grosmannia_penicillata - Trichosporium_symbioticum | 12.39674 | 0.709534 | 44 | 17.47166 | 8.25E-13 | 8.2456264039e-13 | *** |
| Leptographium_abietinum - Leptographium_longiclavatum | 1.265626 | 0.709534 | 44 | 1.783742 | 0.782885 | 7.8288491206e-01 | |
| Leptographium_abietinum - Ophiostoma_bicolor | 5.315522 | 0.709534 | 44 | 7.491563 | 1.16E-07 | 1.1567666847e-07 | *** |
| Leptographium_abietinum - Ophiostoma_ips | 2.464611 | 0.709534 | 44 | 3.473562 | 0.040754 | 4.0753851907e-02 | * |
| Leptographium_abietinum - Ophiostoma_montium | 2.018926 | 0.709534 | 44 | 2.845424 | 0.17499 | 1.7499007446e-01 | |
| Leptographium_abietinum - Trichosporium_symbioticum | 8.90717 | 0.709534 | 44 | 12.55354 | 8.63E-13 | 8.6297635704e-13 | *** |
| Leptographium_longiclavatum - Ophiostoma_bicolor | 4.049895 | 0.709534 | 44 | 5.707821 | 4.51E-05 | 4.5137558503e-05 | *** |
| Leptographium_longiclavatum - Ophiostoma_ips | 1.198985 | 0.709534 | 44 | 1.68982 | 0.832543 | 8.3254288131e-01 | |
| Leptographium_longiclavatum - Ophiostoma_montium | 0.7533 | 0.709534 | 44 | 1.061682 | 0.991542 | 9.9154219969e-01 | |
| Leptographium_longiclavatum - Trichosporium_symbioticum | 7.641543 | 0.709534 | 44 | 10.7698 | 4.39E-12 | 4.3949288653e-12 | *** |
| Ophiostoma_bicolor - Ophiostoma_ips | -2.85091 | 0.709534 | 44 | -4.018 | 0.009213 | 9.2127781652e-03 | ** |
| Ophiostoma_bicolor - Ophiostoma_montium | -3.2966 | 0.709534 | 44 | -4.64614 | 0.001395 | 1.3948650633e-03 | ** |
| Ophiostoma_bicolor - Trichosporium_symbioticum | 3.591648 | 0.709534 | 44 | 5.061979 | 0.000373 | 3.7339375565e-04 | *** |
| Ophiostoma_ips - Ophiostoma_montium | -0.44569 | 0.709534 | 44 | -0.62814 | 0.9999 | 9.9990033075e-01 | |
| Ophiostoma_ips - Trichosporium_symbioticum | 6.442558 | 0.709534 | 44 | 9.07998 | 6.47E-10 | 6.4690686141e-10 | *** |
| Ophiostoma_montium - Trichosporium_symbioticum | 6.888244 | 0.709534 | 44 | 9.708118 | 9.01E-11 | 9.0107699080e-11 | *** |

| Sample | emmean | SE | df | lower.CL | upper.CL | .group |
| --- | --- | --- | --- | --- | --- | --- |
| Endoconidiophora_polonica | -8.9E-15 | 0.501717 | 44 | -1.49622 | 1.496221 | a |
| Ceratocystiopsis_brevicomi | 1.2E-16 | 0.501717 | 44 | -1.49622 | 1.496221 | a |
| Trichosporium_symbioticum | 2.665239 | 0.501717 | 44 | 1.169018 | 4.161461 | b |
| Ophiostoma_bicolor | 6.256887 | 0.501717 | 44 | 4.760666 | 7.753109 | c |
| Ophiostoma_ips | 9.107798 | 0.501717 | 44 | 7.611577 | 10.60402 | d |
| Ophiostoma_montium | 9.553483 | 0.501717 | 44 | 8.057262 | 11.0497 | de |
| Leptographium_longiclavatum | 10.30678 | 0.501717 | 44 | 8.810562 | 11.803 | de |
| Ceratocystiopsis_ranaculosus | 10.681 | 0.501717 | 44 | 9.184775 | 12.17722 | de |
| Leptographium_abietinum | 11.57241 | 0.501717 | 44 | 10.07619 | 13.06863 | ef |
| Grosmannia_clavigera | 13.84223 | 0.501717 | 44 | 12.346 | 15.33845 | fg |
| Grosmannia_penicillata | 15.06198 | 0.501717 | 44 | 13.56576 | 16.5582 | g |
|  |  |  |  |  |  |  |

**Others**

Modell

Sqrt_Normal

| Resid. Df | Resid. Dev | Df | Deviance | Pr(>Chi) | p.value_formatted | Significance |
| --- | --- | --- | --- | --- | --- | --- |
| 44 | 887.5998 |  |  |  | NA |  |
| 36 | 7.686 | 8 | 879.9138 | 0 | 0.0000000000e+00 | *** |

| contrast | estimate | SE | df | t.ratio | p.value | p.value_formatted | Significance |
| --- | --- | --- | --- | --- | --- | --- | --- |
| Aspergillus_flavus - Beauveria_bassiana | 1.113376 | 0.292233 | 36 | 3.809894 | 0.013642 | 1.3641774257e-02 | * |
| Aspergillus_flavus - Chaetomium_globosum | 1.113376 | 0.292233 | 36 | 3.809894 | 0.013642 | 1.3641774257e-02 | * |
| Aspergillus_flavus - Esteya_vermicola | 1.113376 | 0.292233 | 36 | 3.809894 | 0.013642 | 1.3641774257e-02 | * |
| Aspergillus_flavus - Ophiostoma_minus | -6.91215 | 0.292233 | 36 | -23.6529 | 0 | 0.0000000000e+00 | *** |
| Aspergillus_flavus - Ophiostoma_quercus | -9.92889 | 0.292233 | 36 | -33.976 | 0 | 0.0000000000e+00 | *** |
| Aspergillus_flavus - Paracremonium_pembeum | 1.113376 | 0.292233 | 36 | 3.809894 | 0.013642 | 1.3641774257e-02 | * |
| Aspergillus_flavus - Penicillium_commune | 1.113376 | 0.292233 | 36 | 3.809894 | 0.013642 | 1.3641774257e-02 | * |
| Aspergillus_flavus - Sporothrix_stenoceras | -8.05957 | 0.292233 | 36 | -27.5793 | 0 | 0.0000000000e+00 | *** |
| Beauveria_bassiana - Chaetomium_globosum | -2.4E-15 | 0.292233 | 36 | -8.4E-15 | 1 | 1.0000000000e+00 | |
| Beauveria_bassiana - Esteya_vermicola | 1.55E-15 | 0.292233 | 36 | 5.32E-15 | 1 | 1.0000000000e+00 | |
| Beauveria_bassiana - Ophiostoma_minus | -8.02553 | 0.292233 | 36 | -27.4628 | 0 | 0.0000000000e+00 | *** |
| Beauveria_bassiana - Ophiostoma_quercus | -11.0423 | 0.292233 | 36 | -37.7858 | 0 | 0.0000000000e+00 | *** |
| Beauveria_bassiana - Paracremonium_pembeum | -6.7E-16 | 0.292233 | 36 | -2.3E-15 | 1 | 1.0000000000e+00 | |
| Beauveria_bassiana - Penicillium_commune | -6.7E-16 | 0.292233 | 36 | -2.3E-15 | 1 | 1.0000000000e+00 | |
| Beauveria_bassiana - Sporothrix_stenoceras | -9.17295 | 0.292233 | 36 | -31.3892 | 0 | 0.0000000000e+00 | *** |
| Chaetomium_globosum - Esteya_vermicola | 4E-15 | 0.292233 | 36 | 1.37E-14 | 1 | 1.0000000000e+00 | |
| Chaetomium_globosum - Ophiostoma_minus | -8.02553 | 0.292233 | 36 | -27.4628 | 0 | 0.0000000000e+00 | *** |
| Chaetomium_globosum - Ophiostoma_quercus | -11.0423 | 0.292233 | 36 | -37.7858 | 0 | 0.0000000000e+00 | *** |
| Chaetomium_globosum - Paracremonium_pembeum | 1.78E-15 | 0.292233 | 36 | 6.08E-15 | 1 | 1.0000000000e+00 | |
| Chaetomium_globosum - Penicillium_commune | 1.78E-15 | 0.292233 | 36 | 6.08E-15 | 1 | 1.0000000000e+00 | |
| Chaetomium_globosum - Sporothrix_stenoceras | -9.17295 | 0.292233 | 36 | -31.3892 | 0 | 0.0000000000e+00 | *** |
| Esteya_vermicola - Ophiostoma_minus | -8.02553 | 0.292233 | 36 | -27.4628 | 0 | 0.0000000000e+00 | *** |
| Esteya_vermicola - Ophiostoma_quercus | -11.0423 | 0.292233 | 36 | -37.7858 | 0 | 0.0000000000e+00 | *** |
| Esteya_vermicola - Paracremonium_pembeum | -2.2E-15 | 0.292233 | 36 | -7.6E-15 | 1 | 1.0000000000e+00 | |
| Esteya_vermicola - Penicillium_commune | -2.2E-15 | 0.292233 | 36 | -7.6E-15 | 1 | 1.0000000000e+00 | |
| Esteya_vermicola - Sporothrix_stenoceras | -9.17295 | 0.292233 | 36 | -31.3892 | 0 | 0.0000000000e+00 | *** |
| Ophiostoma_minus - Ophiostoma_quercus | -3.01674 | 0.292233 | 36 | -10.3231 | 9.24E-11 | 9.2362673065e-11 | *** |
| Ophiostoma_minus - Paracremonium_pembeum | 8.025526 | 0.292233 | 36 | 27.46278 | 0 | 0.0000000000e+00 | *** |
| Ophiostoma_minus - Penicillium_commune | 8.025526 | 0.292233 | 36 | 27.46278 | 0 | 0.0000000000e+00 | *** |
| Ophiostoma_minus - Sporothrix_stenoceras | -1.14742 | 0.292233 | 36 | -3.92639 | 0.009982 | 9.9822159396e-03 | ** |
| Ophiostoma_quercus - Paracremonium_pembeum | 11.04226 | 0.292233 | 36 | 37.78585 | 0 | 0.0000000000e+00 | *** |
| Ophiostoma_quercus - Penicillium_commune | 11.04226 | 0.292233 | 36 | 37.78585 | 0 | 0.0000000000e+00 | *** |
| Ophiostoma_quercus - Sporothrix_stenoceras | 1.869318 | 0.292233 | 36 | 6.396676 | 6.85E-06 | 6.8455289146e-06 | *** |
| Paracremonium_pembeum - Penicillium_commune | 0 | 0.292233 | 36 | 0 | 1 | 1.0000000000e+00 | |
| Paracremonium_pembeum - Sporothrix_stenoceras | -9.17295 | 0.292233 | 36 | -31.3892 | 0 | 0.0000000000e+00 | *** |
| Penicillium_commune - Sporothrix_stenoceras | -9.17295 | 0.292233 | 36 | -31.3892 | 0 | 0.0000000000e+00 | *** |

| Sample | emmean | SE | df | lower.CL | upper.CL | .group |
| --- | --- | --- | --- | --- | --- | --- |
| Esteya_vermicola | -1.3E-15 | 0.20664 | 36 | -0.60779 | 0.607793 | a |
| Beauveria_bassiana | 2.22E-16 | 0.20664 | 36 | -0.60779 | 0.607793 | a |
| Paracremonium_pembeum | 8.88E-16 | 0.20664 | 36 | -0.60779 | 0.607793 | a |
| Penicillium_commune | 8.88E-16 | 0.20664 | 36 | -0.60779 | 0.607793 | a |
| Chaetomium_globosum | 2.66E-15 | 0.20664 | 36 | -0.60779 | 0.607793 | a |
| Aspergillus_flavus | 1.113376 | 0.20664 | 36 | 0.505583 | 1.721169 | b |
| Ophiostoma_minus | 8.025526 | 0.20664 | 36 | 7.417733 | 8.633318 | c |
| Sporothrix_stenoceras | 9.172945 | 0.20664 | 36 | 8.565153 | 9.780738 | d |
| Ophiostoma_quercus | 11.04226 | 0.20664 | 36 | 10.43447 | 11.65006 | e |

**Ethyl acetate**

Ambrosia beetle fungi

Modell

Normal_log

| Resid. Df | Resid. Dev | Df | Deviance | Pr(>Chi) | p.value_formatted | Significance |
| --- | --- | --- | --- | --- | --- | --- |
| 49 | 195.0804 |  |  |  | NA |  |
| 40 | 30.28907 | 9 | 164.7913 | 6.61E-42 | 6.6093990062e-42 | *** |

| contrast | estimate | SE | df | t.ratio | p.value | p.value_formatted | Significance |
| --- | --- | --- | --- | --- | --- | --- | --- |
| Ambrosiella_grosmanniae - Ambrosiella_hartigii | -4.46941 | 0.550355 | 40 | -8.12096 | 2.35E-08 | 2.3523308856e-08 | *** |
| Ambrosiella_grosmanniae - Ambrosiella_roeperi | -4.2095 | 0.550355 | 40 | -7.64869 | 1.03E-07 | 1.0260605054e-07 | *** |
| Ambrosiella_grosmanniae - Ascoidea_hylecoeti | -1.03697 | 0.550355 | 40 | -1.88418 | 0.679058 | 6.7905754340e-01 | |
| Ambrosiella_grosmanniae - Fusarium_euwallaceae | 1.637942 | 0.550355 | 40 | 2.976155 | 0.11834 | 1.1833996321e-01 | |
| Ambrosiella_grosmanniae - Graphium_euwallaceae | -0.6698 | 0.550355 | 40 | -1.21704 | 0.965148 | 9.6514828837e-01 | |
| Ambrosiella_grosmanniae - Raffaelea_amase | -2.28221 | 0.550355 | 40 | -4.1468 | 0.005881 | 5.8807875841e-03 | ** |
| Ambrosiella_grosmanniae - Raffaelea_canadensis | -1.49589 | 0.550355 | 40 | -2.71805 | 0.201151 | 2.0115107035e-01 | |
| Ambrosiella_grosmanniae - Raffaelea_montetyi | -2.35199 | 0.550355 | 40 | -4.27358 | 0.004078 | 4.0776883358e-03 | ** |
| Ambrosiella_grosmanniae - Raffaelea_sulphurea | 0.070065 | 0.550355 | 40 | 0.12731 | 1 | 9.9999999977e-01 | |
| Ambrosiella_hartigii - Ambrosiella_roeperi | 0.259917 | 0.550355 | 40 | 0.472271 | 0.999976 | 9.9997568614e-01 | |
| Ambrosiella_hartigii - Ascoidea_hylecoeti | 3.432447 | 0.550355 | 40 | 6.236787 | 9.14E-06 | 9.1364254321e-06 | *** |
| Ambrosiella_hartigii - Fusarium_euwallaceae | 6.107356 | 0.550355 | 40 | 11.09712 | 4.41E-12 | 4.4113601660e-12 | *** |
| Ambrosiella_hartigii - Graphium_euwallaceae | 3.799612 | 0.550355 | 40 | 6.903928 | 1.08E-06 | 1.0838687873e-06 | *** |
| Ambrosiella_hartigii - Raffaelea_amase | 2.187204 | 0.550355 | 40 | 3.974168 | 0.009586 | 9.5864104599e-03 | ** |
| Ambrosiella_hartigii - Raffaelea_canadensis | 2.973522 | 0.550355 | 40 | 5.402916 | 0.00013 | 1.2975605180e-04 | *** |
| Ambrosiella_hartigii - Raffaelea_montetyi | 2.117425 | 0.550355 | 40 | 3.84738 | 0.013615 | 1.3614670004e-02 | * |
| Ambrosiella_hartigii - Raffaelea_sulphurea | 4.539479 | 0.550355 | 40 | 8.248274 | 1.59E-08 | 1.5871653214e-08 | *** |
| Ambrosiella_roeperi - Ascoidea_hylecoeti | 3.17253 | 0.550355 | 40 | 5.764515 | 4.13E-05 | 4.1261186679e-05 | *** |
| Ambrosiella_roeperi - Fusarium_euwallaceae | 5.847439 | 0.550355 | 40 | 10.62485 | 1.49E-11 | 1.4948264848e-11 | *** |
| Ambrosiella_roeperi - Graphium_euwallaceae | 3.539695 | 0.550355 | 40 | 6.431657 | 4.9E-06 | 4.8984559460e-06 | *** |
| Ambrosiella_roeperi - Raffaelea_amase | 1.927287 | 0.550355 | 40 | 3.501897 | 0.033995 | 3.3995139615e-02 | * |
| Ambrosiella_roeperi - Raffaelea_canadensis | 2.713605 | 0.550355 | 40 | 4.930645 | 0.000566 | 5.6632142861e-04 | *** |
| Ambrosiella_roeperi - Raffaelea_montetyi | 1.857508 | 0.550355 | 40 | 3.375109 | 0.046748 | 4.6748414557e-02 | * |
| Ambrosiella_roeperi - Raffaelea_sulphurea | 4.279562 | 0.550355 | 40 | 7.776003 | 6.88E-08 | 6.8847827661e-08 | *** |
| Ascoidea_hylecoeti - Fusarium_euwallaceae | 2.674909 | 0.550355 | 40 | 4.860333 | 0.000703 | 7.0301612531e-04 | *** |
| Ascoidea_hylecoeti - Graphium_euwallaceae | 0.367165 | 0.550355 | 40 | 0.667142 | 0.999569 | 9.9956934869e-01 | |
| Ascoidea_hylecoeti - Raffaelea_amase | -1.24524 | 0.550355 | 40 | -2.26262 | 0.433964 | 4.3396438687e-01 | |
| Ascoidea_hylecoeti - Raffaelea_canadensis | -0.45892 | 0.550355 | 40 | -0.83387 | 0.99752 | 9.9752039500e-01 | |
| Ascoidea_hylecoeti - Raffaelea_montetyi | -1.31502 | 0.550355 | 40 | -2.38941 | 0.358724 | 3.5872382240e-01 | |
| Ascoidea_hylecoeti - Raffaelea_sulphurea | 1.107032 | 0.550355 | 40 | 2.011488 | 0.596396 | 5.9639598227e-01 | |
| Fusarium_euwallaceae - Graphium_euwallaceae | -2.30774 | 0.550355 | 40 | -4.19319 | 0.005147 | 5.1468014495e-03 | ** |
| Fusarium_euwallaceae - Raffaelea_amase | -3.92015 | 0.550355 | 40 | -7.12295 | 5.4E-07 | 5.3997103899e-07 | *** |
| Fusarium_euwallaceae - Raffaelea_canadensis | -3.13383 | 0.550355 | 40 | -5.6942 | 5.16E-05 | 5.1599745506e-05 | *** |
| Fusarium_euwallaceae - Raffaelea_montetyi | -3.98993 | 0.550355 | 40 | -7.24974 | 3.61E-07 | 3.6116872315e-07 | *** |
| Fusarium_euwallaceae - Raffaelea_sulphurea | -1.56788 | 0.550355 | 40 | -2.84885 | 0.154918 | 1.5491825060e-01 | |
| Graphium_euwallaceae - Raffaelea_amase | -1.61241 | 0.550355 | 40 | -2.92976 | 0.130761 | 1.3076136111e-01 | |
| Graphium_euwallaceae - Raffaelea_canadensis | -0.82609 | 0.550355 | 40 | -1.50101 | 0.883844 | 8.8384351149e-01 | |
| Graphium_euwallaceae - Raffaelea_montetyi | -1.68219 | 0.550355 | 40 | -3.05655 | 0.099116 | 9.9116184047e-02 | . |
| Graphium_euwallaceae - Raffaelea_sulphurea | 0.739868 | 0.550355 | 40 | 1.344346 | 0.936528 | 9.3652803972e-01 | |
| Raffaelea_amase - Raffaelea_canadensis | 0.786319 | 0.550355 | 40 | 1.428748 | 0.910639 | 9.1063851214e-01 | |
| Raffaelea_amase - Raffaelea_montetyi | -0.06978 | 0.550355 | 40 | -0.12679 | 1 | 9.9999999978e-01 | |
| Raffaelea_amase - Raffaelea_sulphurea | 2.352276 | 0.550355 | 40 | 4.274106 | 0.004072 | 4.0715049038e-03 | ** |
| Raffaelea_canadensis - Raffaelea_montetyi | -0.8561 | 0.550355 | 40 | -1.55554 | 0.860813 | 8.6081349388e-01 | |
| Raffaelea_canadensis - Raffaelea_sulphurea | 1.565957 | 0.550355 | 40 | 2.845358 | 0.156034 | 1.5603357551e-01 | |
| Raffaelea_montetyi - Raffaelea_sulphurea | 2.422054 | 0.550355 | 40 | 4.400894 | 0.002808 | 2.8076145527e-03 | ** |

| Sample | emmean | SE | df | lower.CL | upper.CL | .group |
| --- | --- | --- | --- | --- | --- | --- |
| Fusarium_euwallaceae | 1.676795 | 0.38916 | 40 | 0.523899 | 2.829691 | a |
| Raffaelea_sulphurea | 3.244672 | 0.38916 | 40 | 2.091776 | 4.397568 | ab |
| Ambrosiella_grosmanniae | 3.314737 | 0.38916 | 40 | 2.161841 | 4.467634 | ab |
| Graphium_euwallaceae | 3.984539 | 0.38916 | 40 | 2.831643 | 5.137436 | bc |
| Ascoidea_hylecoeti | 4.351704 | 0.38916 | 40 | 3.198808 | 5.5046 | bc |
| Raffaelea_canadensis | 4.810629 | 0.38916 | 40 | 3.657733 | 5.963525 | bc |
| Raffaelea_amase | 5.596947 | 0.38916 | 40 | 4.444051 | 6.749844 | c |
| Raffaelea_montetyi | 5.666726 | 0.38916 | 40 | 4.51383 | 6.819622 | c |
| Ambrosiella_roeperi | 7.524234 | 0.38916 | 40 | 6.371338 | 8.67713 | d |
| Ambrosiella_hartigii | 7.784151 | 0.38916 | 40 | 6.631255 | 8.937047 | d |

Bark beetle fungi

Modell

Sqrt_Normal

| Resid. Df | Resid. Dev | Df | Deviance | Pr(>Chi) | p.value_formatted | Significance |
| --- | --- | --- | --- | --- | --- | --- |
| 54 | 4497.608 |  |  |  | NA |  |
| 44 | 190.3302 | 10 | 4307.277 | 1.5E-207 | 1.5440060579e-207 | *** |

| contrast | estimate | SE | df | t.ratio | p.value | p.value_formatted | Significance |
| --- | --- | --- | --- | --- | --- | --- | --- |
| Ceratocystiopsis_brevicomi - Ceratocystiopsis_ranaculosus | 0.309605 | 1.315399 | 44 | 0.23537 | 1 | 9.9999999190e-01 | |
| Ceratocystiopsis_brevicomi - Endoconidiophora_polonica | -22.6119 | 1.315399 | 44 | -17.1901 | 8.25E-13 | 8.2456264039e-13 | *** |
| Ceratocystiopsis_brevicomi - Grosmannia_clavigera | -18.2304 | 1.315399 | 44 | -13.8592 | 8.28E-13 | 8.2833739867e-13 | *** |
| Ceratocystiopsis_brevicomi - Grosmannia_penicillata | -7.91374 | 1.315399 | 44 | -6.01623 | 1.62E-05 | 1.6175503406e-05 | *** |
| Ceratocystiopsis_brevicomi - Leptographium_abietinum | 2.105457 | 1.315399 | 44 | 1.600623 | 0.873585 | 8.7358544046e-01 | |
| Ceratocystiopsis_brevicomi - Leptographium_longiclavatum | -12.7434 | 1.315399 | 44 | -9.68785 | 9.59E-11 | 9.5924157506e-11 | *** |
| Ceratocystiopsis_brevicomi - Ophiostoma_bicolor | -0.1048 | 1.315399 | 44 | -0.07967 | 1 | 1.0000000000e+00 | |
| Ceratocystiopsis_brevicomi - Ophiostoma_ips | 1.180787 | 1.315399 | 44 | 0.897665 | 0.997769 | 9.9776859491e-01 | |
| Ceratocystiopsis_brevicomi - Ophiostoma_montium | 2.235987 | 1.315399 | 44 | 1.699854 | 0.827538 | 8.2753773102e-01 | |
| Ceratocystiopsis_brevicomi - Trichosporium_symbioticum | 4.532327 | 1.315399 | 44 | 3.445591 | 0.043774 | 4.3774376752e-02 | * |
| Ceratocystiopsis_ranaculosus - Endoconidiophora_polonica | -22.9215 | 1.315399 | 44 | -17.4255 | 8.25E-13 | 8.2456264039e-13 | *** |
| Ceratocystiopsis_ranaculosus - Grosmannia_clavigera | -18.54 | 1.315399 | 44 | -14.0946 | 8.27E-13 | 8.2711615335e-13 | *** |
| Ceratocystiopsis_ranaculosus - Grosmannia_penicillata | -8.22334 | 1.315399 | 44 | -6.2516 | 7.36E-06 | 7.3610216024e-06 | *** |
| Ceratocystiopsis_ranaculosus - Leptographium_abietinum | 1.795853 | 1.315399 | 44 | 1.365253 | 0.950431 | 9.5043087875e-01 | |
| Ceratocystiopsis_ranaculosus - Leptographium_longiclavatum | -13.053 | 1.315399 | 44 | -9.92322 | 4.67E-11 | 4.6674220044e-11 | *** |
| Ceratocystiopsis_ranaculosus - Ophiostoma_bicolor | -0.4144 | 1.315399 | 44 | -0.31504 | 1 | 9.9999985809e-01 | |
| Ceratocystiopsis_ranaculosus - Ophiostoma_ips | 0.871183 | 1.315399 | 44 | 0.662295 | 0.999839 | 9.9983910124e-01 | |
| Ceratocystiopsis_ranaculosus - Ophiostoma_montium | 1.926382 | 1.315399 | 44 | 1.464485 | 0.923602 | 9.2360213872e-01 | |
| Ceratocystiopsis_ranaculosus - Trichosporium_symbioticum | 4.222722 | 1.315399 | 44 | 3.210222 | 0.078113 | 7.8112653271e-02 | . |
| Endoconidiophora_polonica - Grosmannia_clavigera | 4.381524 | 1.315399 | 44 | 3.330946 | 0.058341 | 5.8341271577e-02 | . |
| Endoconidiophora_polonica - Grosmannia_penicillata | 14.69816 | 1.315399 | 44 | 11.17391 | 1.94E-12 | 1.9352297542e-12 | *** |
| Endoconidiophora_polonica - Leptographium_abietinum | 24.71735 | 1.315399 | 44 | 18.79076 | 8.25E-13 | 8.2456264039e-13 | *** |
| Endoconidiophora_polonica - Leptographium_longiclavatum | 9.868507 | 1.315399 | 44 | 7.502291 | 1.12E-07 | 1.1161798597e-07 | *** |
| Endoconidiophora_polonica - Ophiostoma_bicolor | 22.50709 | 1.315399 | 44 | 17.11047 | 8.25E-13 | 8.2456264039e-13 | *** |
| Endoconidiophora_polonica - Ophiostoma_ips | 23.79268 | 1.315399 | 44 | 18.0878 | 8.25E-13 | 8.2456264039e-13 | *** |
| Endoconidiophora_polonica - Ophiostoma_montium | 24.84788 | 1.315399 | 44 | 18.88999 | 8.25E-13 | 8.2456264039e-13 | *** |
| Endoconidiophora_polonica - Trichosporium_symbioticum | 27.14422 | 1.315399 | 44 | 20.63573 | 8.25E-13 | 8.2456264039e-13 | *** |
| Grosmannia_clavigera - Grosmannia_penicillata | 10.31663 | 1.315399 | 44 | 7.842967 | 3.6E-08 | 3.6042266616e-08 | *** |
| Grosmannia_clavigera - Leptographium_abietinum | 20.33583 | 1.315399 | 44 | 15.45982 | 8.25E-13 | 8.2478468499e-13 | *** |
| Grosmannia_clavigera - Leptographium_longiclavatum | 5.486983 | 1.315399 | 44 | 4.171345 | 0.005894 | 5.8944253990e-03 | ** |
| Grosmannia_clavigera - Ophiostoma_bicolor | 18.12557 | 1.315399 | 44 | 13.77952 | 8.29E-13 | 8.2944762170e-13 | *** |
| Grosmannia_clavigera - Ophiostoma_ips | 19.41116 | 1.315399 | 44 | 14.75686 | 8.26E-13 | 8.2611695262e-13 | *** |
| Grosmannia_clavigera - Ophiostoma_montium | 20.46636 | 1.315399 | 44 | 15.55905 | 8.25E-13 | 8.2478468499e-13 | *** |
| Grosmannia_clavigera - Trichosporium_symbioticum | 22.7627 | 1.315399 | 44 | 17.30478 | 8.25E-13 | 8.2456264039e-13 | *** |
| Grosmannia_penicillata - Leptographium_abietinum | 10.0192 | 1.315399 | 44 | 7.616849 | 7.63E-08 | 7.6257495429e-08 | *** |
| Grosmannia_penicillata - Leptographium_longiclavatum | -4.82965 | 1.315399 | 44 | -3.67162 | 0.024204 | 2.4204401025e-02 | * |
| Grosmannia_penicillata - Ophiostoma_bicolor | 7.808939 | 1.315399 | 44 | 5.936555 | 2.11E-05 | 2.1100635513e-05 | *** |
| Grosmannia_penicillata - Ophiostoma_ips | 9.094526 | 1.315399 | 44 | 6.913891 | 7.98E-07 | 7.9795977859e-07 | *** |
| Grosmannia_penicillata - Ophiostoma_montium | 10.14973 | 1.315399 | 44 | 7.716081 | 5.49E-08 | 5.4860567755e-08 | *** |
| Grosmannia_penicillata - Trichosporium_symbioticum | 12.44607 | 1.315399 | 44 | 9.461817 | 1.94E-10 | 1.9372037308e-10 | *** |
| Leptographium_abietinum - Leptographium_longiclavatum | -14.8488 | 1.315399 | 44 | -11.2885 | 1.63E-12 | 1.6278089987e-12 | *** |
| Leptographium_abietinum - Ophiostoma_bicolor | -2.21026 | 1.315399 | 44 | -1.68029 | 0.837224 | 8.3722357364e-01 | |
| Leptographium_abietinum - Ophiostoma_ips | -0.92467 | 1.315399 | 44 | -0.70296 | 0.999726 | 9.9972595686e-01 | |
| Leptographium_abietinum - Ophiostoma_montium | 0.130529 | 1.315399 | 44 | 0.099232 | 1 | 1.0000000000e+00 | |
| Leptographium_abietinum - Trichosporium_symbioticum | 2.42687 | 1.315399 | 44 | 1.844969 | 0.747445 | 7.4744479258e-01 | |
| Leptographium_longiclavatum - Ophiostoma_bicolor | 12.63859 | 1.315399 | 44 | 9.608177 | 1.23E-10 | 1.2275125361e-10 | *** |
| Leptographium_longiclavatum - Ophiostoma_ips | 13.92417 | 1.315399 | 44 | 10.58551 | 6.97E-12 | 6.9698691263e-12 | *** |
| Leptographium_longiclavatum - Ophiostoma_montium | 14.97937 | 1.315399 | 44 | 11.3877 | 1.44E-12 | 1.4381829061e-12 | *** |
| Leptographium_longiclavatum - Trichosporium_symbioticum | 17.27571 | 1.315399 | 44 | 13.13344 | 8.37E-13 | 8.3688611596e-13 | *** |
| Ophiostoma_bicolor - Ophiostoma_ips | 1.285587 | 1.315399 | 44 | 0.977336 | 0.99556 | 9.9555983518e-01 | |
| Ophiostoma_bicolor - Ophiostoma_montium | 2.340787 | 1.315399 | 44 | 1.779526 | 0.785242 | 7.8524248170e-01 | |
| Ophiostoma_bicolor - Trichosporium_symbioticum | 4.637127 | 1.315399 | 44 | 3.525263 | 0.035659 | 3.5658715817e-02 | * |
| Ophiostoma_ips - Ophiostoma_montium | 1.055199 | 1.315399 | 44 | 0.80219 | 0.999133 | 9.9913266203e-01 | |
| Ophiostoma_ips - Trichosporium_symbioticum | 3.35154 | 1.315399 | 44 | 2.547926 | 0.306383 | 3.0638348420e-01 | |
| Ophiostoma_montium - Trichosporium_symbioticum | 2.29634 | 1.315399 | 44 | 1.745737 | 0.803717 | 8.0371655731e-01 | |

| Sample | emmean | SE | df | lower.CL | upper.CL | .group |
| --- | --- | --- | --- | --- | --- | --- |
| Trichosporium_symbioticum | 0 | 0.930128 | 44 | -2.77383 | 2.77383 | a |
| Ophiostoma_montium | 2.29634 | 0.930128 | 44 | -0.47749 | 5.07017 | ab |
| Leptographium_abietinum | 2.42687 | 0.930128 | 44 | -0.34696 | 5.2007 | ab |
| Ophiostoma_ips | 3.35154 | 0.930128 | 44 | 0.57771 | 6.12537 | ab |
| Ceratocystiopsis_ranaculosus | 4.222722 | 0.930128 | 44 | 1.448892 | 6.996552 | ab |
| Ceratocystiopsis_brevicomi | 4.532327 | 0.930128 | 44 | 1.758497 | 7.306157 | b |
| Ophiostoma_bicolor | 4.637127 | 0.930128 | 44 | 1.863297 | 7.410957 | b |
| Grosmannia_penicillata | 12.44607 | 0.930128 | 44 | 9.672236 | 15.2199 | c |
| Leptographium_longiclavatum | 17.27571 | 0.930128 | 44 | 14.50188 | 20.04954 | d |
| Grosmannia_clavigera | 22.7627 | 0.930128 | 44 | 19.98887 | 25.53653 | e |
| Endoconidiophora_polonica | 27.14422 | 0.930128 | 44 | 24.37039 | 29.91805 | e |

Others

Modell

Sqrt_Normal

| Resid. Df | Resid. Dev | Df | Deviance | Pr(>Chi) | p.value_formatted | Significance |
| --- | --- | --- | --- | --- | --- | --- |
| 44 | 523.6233 |  |  |  | NA |  |
| 36 | 139.851 | 8 | 383.7723 | 7.55E-18 | 7.5451651001e-18 | *** |

| contrast | estimate | SE | df | t.ratio | p.value | p.value_formatted | Significance |
| --- | --- | --- | --- | --- | --- | --- | --- |
| Aspergillus_flavus - Beauveria_bassiana | -0.93173 | 1.246555 | 36 | -0.74745 | 0.997601 | 9.9760101115e-01 | |
| Aspergillus_flavus - Caetomium_globosum | -1.46711 | 1.246555 | 36 | -1.17693 | 0.956349 | 9.5634937223e-01 | |
| Aspergillus_flavus - Esteya_vermicola | 3.091539 | 1.246555 | 36 | 2.480065 | 0.274935 | 2.7493532022e-01 | |
| Aspergillus_flavus - Ophiostoma_minus | 1.436115 | 1.246555 | 36 | 1.152067 | 0.961359 | 9.6135875060e-01 | |
| Aspergillus_flavus - Ophiostoma_quercus | -2.65058 | 1.246555 | 36 | -2.12633 | 0.472445 | 4.7244502381e-01 | |
| Aspergillus_flavus - Paracremonium_pembeum | -2.03816 | 1.246555 | 36 | -1.63504 | 0.779544 | 7.7954399469e-01 | |
| Aspergillus_flavus - Penicillium_commune | -0.23792 | 1.246555 | 36 | -0.19086 | 1 | 9.9999992843e-01 | |
| Aspergillus_flavus - Sporothrix_stenoceras | -7.98767 | 1.246555 | 36 | -6.40779 | 6.62E-06 | 6.6189546547e-06 | *** |
| Beauveria_bassiana - Caetomium_globosum | -0.53538 | 1.246555 | 36 | -0.42949 | 0.999959 | 9.9995931190e-01 | |
| Beauveria_bassiana - Esteya_vermicola | 4.023271 | 1.246555 | 36 | 3.227511 | 0.058964 | 5.8964200799e-02 | . |
| Beauveria_bassiana - Ophiostoma_minus | 2.367847 | 1.246555 | 36 | 1.899512 | 0.618386 | 6.1838581383e-01 | |
| Beauveria_bassiana - Ophiostoma_quercus | -1.71885 | 1.246555 | 36 | -1.37888 | 0.898419 | 8.9841949244e-01 | |
| Beauveria_bassiana - Paracremonium_pembeum | -1.10643 | 1.246555 | 36 | -0.88759 | 0.99231 | 9.9230951235e-01 | |
| Beauveria_bassiana - Penicillium_commune | 0.693812 | 1.246555 | 36 | 0.556583 | 0.999713 | 9.9971320802e-01 | |
| Beauveria_bassiana - Sporothrix_stenoceras | -7.05594 | 1.246555 | 36 | -5.66035 | 6.38E-05 | 6.3774674211e-05 | *** |
| Caetomium_globosum - Esteya_vermicola | 4.558651 | 1.246555 | 36 | 3.656999 | 0.020374 | 2.0373752225e-02 | * |
| Caetomium_globosum - Ophiostoma_minus | 2.903228 | 1.246555 | 36 | 2.329 | 0.352438 | 3.5243820617e-01 | |
| Caetomium_globosum - Ophiostoma_quercus | -1.18347 | 1.246555 | 36 | -0.94939 | 0.988099 | 9.8809921174e-01 | |
| Caetomium_globosum - Paracremonium_pembeum | -0.57105 | 1.246555 | 36 | -0.4581 | 0.999933 | 9.9993349462e-01 | |
| Caetomium_globosum - Penicillium_commune | 1.229193 | 1.246555 | 36 | 0.986071 | 0.984867 | 9.8486723895e-01 | |
| Caetomium_globosum - Sporothrix_stenoceras | -6.52056 | 1.246555 | 36 | -5.23086 | 0.000233 | 2.3264658410e-04 | *** |
| Esteya_vermicola - Ophiostoma_minus | -1.65542 | 1.246555 | 36 | -1.328 | 0.916039 | 9.1603918681e-01 | |
| Esteya_vermicola - Ophiostoma_quercus | -5.74212 | 1.246555 | 36 | -4.60639 | 0.001473 | 1.4733296352e-03 | ** |
| Esteya_vermicola - Paracremonium_pembeum | -5.1297 | 1.246555 | 36 | -4.1151 | 0.005953 | 5.9532364875e-03 | ** |
| Esteya_vermicola - Penicillium_commune | -3.32946 | 1.246555 | 36 | -2.67093 | 0.194287 | 1.9428689824e-01 | |
| Esteya_vermicola - Sporothrix_stenoceras | -11.0792 | 1.246555 | 36 | -8.88786 | 4.57E-09 | 4.5662443826e-09 | *** |
| Ophiostoma_minus - Ophiostoma_quercus | -4.0867 | 1.246555 | 36 | -3.27839 | 0.052281 | 5.2281306118e-02 | . |
| Ophiostoma_minus - Paracremonium_pembeum | -3.47428 | 1.246555 | 36 | -2.7871 | 0.154655 | 1.5465472888e-01 | |
| Ophiostoma_minus - Penicillium_commune | -1.67403 | 1.246555 | 36 | -1.34293 | 0.911085 | 9.1108485020e-01 | |
| Ophiostoma_minus - Sporothrix_stenoceras | -9.42378 | 1.246555 | 36 | -7.55986 | 2.1E-07 | 2.0991146688e-07 | *** |
| Ophiostoma_quercus - Paracremonium_pembeum | 0.612418 | 1.246555 | 36 | 0.491288 | 0.999887 | 9.9988712630e-01 | |
| Ophiostoma_quercus - Penicillium_commune | 2.412662 | 1.246555 | 36 | 1.935463 | 0.595107 | 5.9510707465e-01 | |
| Ophiostoma_quercus - Sporothrix_stenoceras | -5.33709 | 1.246555 | 36 | -4.28147 | 0.003738 | 3.7376058461e-03 | ** |
| Paracremonium_pembeum - Penicillium_commune | 1.800244 | 1.246555 | 36 | 1.444175 | 0.87277 | 8.7277046171e-01 | |
| Paracremonium_pembeum - Sporothrix_stenoceras | -5.94951 | 1.246555 | 36 | -4.77276 | 0.000906 | 9.0648586899e-04 | *** |
| Penicillium_commune - Sporothrix_stenoceras | -7.74975 | 1.246555 | 36 | -6.21693 | 1.18E-05 | 1.1801123220e-05 | *** |

| Sample | emmean | SE | df | lower.CL | upper.CL | .group |
| --- | --- | --- | --- | --- | --- | --- |
| Esteya_vermicola | 5.33E-15 | 0.881448 | 36 | -2.59262 | 2.592615 | a |
| Ophiostoma_minus | 1.655424 | 0.881448 | 36 | -0.93719 | 4.248039 | ab |
| Aspergillus_flavus | 3.091539 | 0.881448 | 36 | 0.498924 | 5.684154 | ab |
| Penicillium_commune | 3.329459 | 0.881448 | 36 | 0.736844 | 5.922074 | ab |
| Beauveria_bassiana | 4.023271 | 0.881448 | 36 | 1.430655 | 6.615886 | ab |
| Caetomium_globosum | 4.558651 | 0.881448 | 36 | 1.966036 | 7.151267 | b |
| Paracremonium_pembeum | 5.129703 | 0.881448 | 36 | 2.537088 | 7.722319 | b |
| Ophiostoma_quercus | 5.742121 | 0.881448 | 36 | 3.149506 | 8.334736 | b |
| Sporothrix_stenoceras | 11.07921 | 0.881448 | 36 | 8.486593 | 13.67182 | c |

**Fig. 5**

**Essential amino acids**

Modell

Normal_log

| Resid. Df | Resid. Dev | Df | Deviance | Pr(>Chi) |
| --- | --- | --- | --- | --- |
| 29 | 12.00175 |  |  |  |
| 27 | 9.512222 | 2 | 2.489531 | 0.029211 |

| contrast | estimate | SE | df | t.ratio | p.value | Signif |
| --- | --- | --- | --- | --- | --- | --- |
| Ambrosia beetle - Bark beetle | -0.3228 | 0.259342 | 27 | -1.24469 | 0.437967 |  |
| Ambrosia beetle - Others | 0.386367 | 0.272719 | 27 | 1.416724 | 0.346676 |  |
| Bark beetle - Others | 0.709167 | 0.266782 | 27 | 2.658226 | 0.033776 | * |

**Non-essential amino acids**

Modell

Normal_log

| Resid. Df | Resid. Dev | Df | Deviance | Pr(>Chi) |
| --- | --- | --- | --- | --- |
| 29 | 7.710529 |  |  |  |
| 27 | 4.691581 | 2 | 3.018947 | 0.000169 |

| contrast | estimate | SE | df | t.ratio | p.value | Signif |
| --- | --- | --- | --- | --- | --- | --- |
| Ambrosia beetle - Bark beetle | -0.62357 | 0.182134 | 27 | -3.42371 | 0.005458 | ** |
| Ambrosia beetle - Others | 0.068202 | 0.191528 | 27 | 0.356096 | 0.932664 |  |
| Bark beetle - Others | 0.691776 | 0.187359 | 27 | 3.692245 | 0.002765 | ** |

**Individual amino acids**

**Ala**

Modell

Normal_log

| Resid. Df | Resid. Dev | Df | Deviance | Pr(>Chi) |
| --- | --- | --- | --- | --- |
| 29 | 9.231326 |  |  |  |
| 27 | 6.461873 | 2 | 2.769453 | 0.003071 |

| contrast | estimate | SE | df | t.ratio | p.value | Signif |
| --- | --- | --- | --- | --- | --- | --- |
| Ambrosia beetle - Bark beetle | -0.32069 | 0.213752 | 27 | -1.50028 | 0.306657 |  |
| Ambrosia beetle - Others | 0.427075 | 0.224778 | 27 | 1.899988 | 0.15807 |  |
| Bark beetle - Others | 0.747763 | 0.219885 | 27 | 3.400706 | 0.005781 | ** |

**Ser**

Modell

Sqrt_Normal

| Resid. Df | Resid. Dev | Df | Deviance | Pr(>Chi) |
| --- | --- | --- | --- | --- |
| 29 | 6.536825 |  |  |  |
| 27 | 4.882468 | 2 | 1.654357 | 0.010314 |

| contrast | estimate | SE | df | t.ratio | p.value | Signif |
| --- | --- | --- | --- | --- | --- | --- |
| Ambrosia beetle - Bark beetle | -0.29383 | 0.185802 | 27 | -1.5814 | 0.270744 |  |
| Ambrosia beetle - Others | 0.282913 | 0.195386 | 27 | 1.44797 | 0.331359 |  |
| Bark beetle - Others | 0.57674 | 0.191133 | 27 | 3.017485 | 0.014727 | * |

**Pro**

Modell

Sqrt_Normal

| Resid. Df | Resid. Dev | Df | Deviance | Pr(>Chi) |
| --- | --- | --- | --- | --- |
| 29 | 5.089222 |  |  |  |
| 27 | 5.074862 | 2 | 0.01436 | 0.962519 |

| contrast | estimate | SE | df | t.ratio | p.value |
| --- | --- | --- | --- | --- | --- |
| Ambrosia beetle - Bark beetle | -0.03698 | 0.189428 | 27 | -0.19522 | 0.979226 |
| Ambrosia beetle - Others | 0.01443 | 0.199198 | 27 | 0.072439 | 0.997111 |
| Bark beetle - Others | 0.051409 | 0.194862 | 27 | 0.263823 | 0.962411 |

**Val**

Modell

Sqrt_Normal

| Resid. Df | Resid. Dev | Df | Deviance | Pr(>Chi) |
| --- | --- | --- | --- | --- |
| 28 | 4.482524 |  |  |  |
| 26 | 4.052473 | 2 | 0.430051 | 0.251687 |

| contrast | estimate | SE | df | t.ratio | p.value |
| --- | --- | --- | --- | --- | --- |
| Ambrosia beetle - Bark beetle | 0.039775 | 0.177448 | 26 | 0.224153 | 0.972711 |
| Ambrosia beetle - Others | 0.282691 | 0.186109 | 26 | 1.518956 | 0.29873 |
| Bark beetle - Others | 0.242916 | 0.177448 | 26 | 1.368942 | 0.371391 |

**Thr**

Modell

Normal_log

| Resid. Df | Resid. Dev | Df | Deviance | Pr(>Chi) |
| --- | --- | --- | --- | --- |
| 29 | 9.66586 |  |  |  |
| 27 | 7.407776 | 2 | 2.258084 | 0.016323 |

| contrast | estimate | SE | df | t.ratio | p.value | Signif |
| --- | --- | --- | --- | --- | --- | --- |
| Ambrosia beetle - Bark beetle | -0.44125 | 0.228863 | 27 | -1.928 | 0.150239 |  |
| Ambrosia beetle - Others | 0.212205 | 0.240668 | 27 | 0.881733 | 0.656216 |  |
| Bark beetle - Others | 0.653451 | 0.235429 | 27 | 2.775581 | 0.025903 | * |

**Phe**

Modell

Gamma_log

| Resid. Df | Resid. Dev | Df | Deviance | Pr(>Chi) |
| --- | --- | --- | --- | --- |
| 29 | 15.7993 |  |  |  |
| 27 | 15.44272 | 2 | 0.356581 | 0.669044 |

| contrast | estimate | SE | df | t.ratio | p.value |
| --- | --- | --- | --- | --- | --- |
| Ambrosia beetle - Bark beetle | 0.124271 | 0.291015 | 27 | 0.427028 | 0.904726 |
| Ambrosia beetle - Others | 0.275434 | 0.306025 | 27 | 0.900036 | 0.644924 |
| Bark beetle - Others | 0.151162 | 0.299364 | 27 | 0.504945 | 0.869562 |

**Iso**

Modell

Sqrt_Normal

| Resid. Df | Resid. Dev | Df | Deviance | Pr(>Chi) |
| --- | --- | --- | --- | --- |
| 29 | 5.226363 |  |  |  |
| 27 | 4.646516 | 2 | 0.579847 | 0.185502 |

| contrast | estimate | SE | df | t.ratio | p.value |
| --- | --- | --- | --- | --- | --- |
| Ambrosia beetle - Bark beetle | 0.139655 | 0.181257 | 27 | 0.770477 | 0.723998 |
| Ambrosia beetle - Others | 0.348512 | 0.190606 | 27 | 1.828441 | 0.179535 |
| Bark beetle - Others | 0.208858 | 0.186457 | 27 | 1.120139 | 0.510244 |

**Asp**

Modell

Normal_log

| Resid. Df | Resid. Dev | Df | Deviance | Pr(>Chi) |
| --- | --- | --- | --- | --- |
| 29 | 10.40016 |  |  |  |
| 27 | 10.29669 | 2 | 0.10347 | 0.873139 |

| contrast | estimate | SE | df | t.ratio | p.value |
| --- | --- | --- | --- | --- | --- |
| Ambrosia beetle - Bark beetle | 0.139999 | 0.269824 | 27 | 0.518853 | 0.862842 |
| Ambrosia beetle - Others | 0.084639 | 0.283741 | 27 | 0.298296 | 0.95222 |
| Bark beetle - Others | -0.05536 | 0.277565 | 27 | -0.19945 | 0.978326 |

**Glu**

Modell

Normal_log

| Resid. Df | Resid. Dev | Df | Deviance | Pr(>Chi) |
| --- | --- | --- | --- | --- |
| 29 | 8.306732 |  |  |  |
| 27 | 6.31939 | 2 | 1.987342 | 0.014328 |

| contrast | estimate | SE | df | t.ratio | p.value | Signif |
| --- | --- | --- | --- | --- | --- | --- |
| Ambrosia beetle - Bark beetle | -0.61564 | 0.211382 | 27 | -2.91246 | 0.01887 | * |
| Ambrosia beetle - Others | -0.34035 | 0.222286 | 27 | -1.53115 | 0.292645 |  |
| Bark beetle - Others | 0.275292 | 0.217447 | 27 | 1.26602 | 0.426056 |  |

**Met**

Modell

Sqrt_Normal

| Resid. Df | Resid. Dev | Df | Deviance | Pr(>Chi) |
| --- | --- | --- | --- | --- |
| 29 | 2.973091 |  |  |  |
| 27 | 2.777435 | 2 | 0.195656 | 0.386352 |

| contrast | estimate | SE | df | t.ratio | p.value |
| --- | --- | --- | --- | --- | --- |
| Ambrosia beetle - Bark beetle | 0.073565 | 0.140137 | 27 | 0.524947 | 0.859858 |
| Ambrosia beetle - Others | 0.201497 | 0.147365 | 27 | 1.367328 | 0.371719 |
| Bark beetle - Others | 0.127932 | 0.144158 | 27 | 0.887448 | 0.652693 |

**Hist**

Modell

Sqrt_Normal

| Resid. Df | Resid. Dev | Df | Deviance | Pr(>Chi) |
| --- | --- | --- | --- | --- |
| 29 | 12.5732 |  |  |  |
| 27 | 7.983229 | 2 | 4.589971 | 0.000426 |

| contrast | estimate | SE | df | t.ratio | p.value | Signif |
| --- | --- | --- | --- | --- | --- | --- |
| Ambrosia beetle - Bark beetle | -0.74973 | 0.237586 | 27 | -3.15564 | 0.010567 | * |
| Ambrosia beetle - Others | 0.118369 | 0.249841 | 27 | 0.473779 | 0.884149 |  |
| Bark beetle - Others | 0.868104 | 0.244402 | 27 | 3.551951 | 0.003952 | ** |

**Leu**

Modell

Sqrt_Normal

| Resid. Df | Resid. Dev | Df | Deviance | Pr(>Chi) |
| --- | --- | --- | --- | --- |
| 29 | 6.210406 |  |  |  |
| 27 | 5.905132 | 2 | 0.305274 | 0.497629 |

| contrast | estimate | SE | df | t.ratio | p.value |
| --- | --- | --- | --- | --- | --- |
| Ambrosia beetle - Bark beetle | 0.124837 | 0.204337 | 27 | 0.610936 | 0.815415 |
| Ambrosia beetle - Others | 0.253802 | 0.214876 | 27 | 1.181153 | 0.474299 |
| Bark beetle - Others | 0.128965 | 0.210199 | 27 | 0.613539 | 0.814007 |

**Arg**

Modell

Sqrt_Normal

| Resid. Df | Resid. Dev | Df | Deviance | Pr(>Chi) |
| --- | --- | --- | --- | --- |
| 29 | 24.63636 |  |  |  |
| 27 | 18.90436 | 2 | 5.732007 | 0.016683 |

| contrast | estimate | SE | df | t.ratio | p.value | Signif |
| --- | --- | --- | --- | --- | --- | --- |
| Ambrosia beetle - Bark beetle | -0.80498 | 0.365605 | 27 | -2.20178 | 0.088985 |  |
| Ambrosia beetle - Others | 0.187497 | 0.384463 | 27 | 0.487686 | 0.877722 |  |
| Bark beetle - Others | 0.99248 | 0.376094 | 27 | 2.638914 | 0.035263 | * |

**Tyr**

Modell

Sqrt_Normal

| Resid. Df | Resid. Dev | Df | Deviance | Pr(>Chi) |
| --- | --- | --- | --- | --- |
| 29 | 1.049086 |  |  |  |
| 27 | 0.892468 | 2 | 0.156619 | 0.093564 |

| contrast | estimate | SE | df | t.ratio | p.value |
| --- | --- | --- | --- | --- | --- |
| Ambrosia beetle - Bark beetle | 0.168512 | 0.079438 | 27 | 2.121311 | 0.104304 |
| Ambrosia beetle - Others | 0.052912 | 0.083535 | 27 | 0.633415 | 0.803149 |
| Bark beetle - Others | -0.1156 | 0.081717 | 27 | -1.41464 | 0.347712 |

**Trp**

Modell

Sqrt_Normal

| Resid. Df | Resid. Dev | Df | Deviance | Pr(>Chi) |
| --- | --- | --- | --- | --- |
| 29 | 1.049086 |  |  |  |
| 27 | 0.892468 | 2 | 0.156619 | 0.093564 |

| contrast | estimate | SE | df | t.ratio | p.value |
| --- | --- | --- | --- | --- | --- |
| Ambrosia beetle - Bark beetle | 0.168512 | 0.079438 | 27 | 2.121311 | 0.104304 |
| Ambrosia beetle - Others | 0.052912 | 0.083535 | 27 | 0.633415 | 0.803149 |
| Bark beetle - Others | -0.1156 | 0.081717 | 27 | -1.41464 | 0.347712 |

**Asn**

Modell

Sqrt_Normal

| Resid. Df | Resid. Dev | Df | Deviance | Pr(>Chi) |
| --- | --- | --- | --- | --- |
| 29 | 6.765223 |  |  |  |
| 27 | 3.654774 | 2 | 3.110449 | 1.02E-05 |

| contrast | estimate | SE | df | t.ratio | p.value | Signif |
| --- | --- | --- | --- | --- | --- | --- |
| Ambrosia beetle - Bark beetle | -0.72442 | 0.160754 | 27 | -4.5064 | 0.000328 | *** |
| Ambrosia beetle - Others | -0.1399 | 0.169046 | 27 | -0.82756 | 0.689459 |  |
| Bark beetle - Others | 0.584526 | 0.165366 | 27 | 3.534747 | 0.004128 | ** |

**Gln**

Modell

Normal_log

| Resid. Df | Resid. Dev | Df | Deviance | Pr(>Chi) |
| --- | --- | --- | --- | --- |
| 29 | 25.46117 |  |  |  |
| 27 | 15.18407 | 2 | 10.2771 | 0.000108 |

| contrast | estimate | SE | df | t.ratio | p.value | Signif |
| --- | --- | --- | --- | --- | --- | --- |
| Ambrosia beetle - Bark beetle | -1.10089 | 0.327662 | 27 | -3.35983 | 0.0064 | ** |
| Ambrosia beetle - Others | 0.213036 | 0.344562 | 27 | 0.61828 | 0.811434 |  |
| Bark beetle - Others | 1.313924 | 0.337062 | 27 | 3.89817 | 0.001626 | ** |

**Lys**

Modell

Sqrt_Normal

| Resid. Df | Resid. Dev | Df | Deviance | Pr(>Chi) |
| --- | --- | --- | --- | --- |
| 29 | 11.18494 |  |  |  |
| 27 | 9.915975 | 2 | 1.268961 | 0.177708 |

| contrast | estimate | SE | df | t.ratio | p.value |
| --- | --- | --- | --- | --- | --- |
| Ambrosia beetle - Bark beetle | -0.30744 | 0.264789 | 27 | -1.16108 | 0.486019 |
| Ambrosia beetle - Others | 0.189436 | 0.278446 | 27 | 0.680334 | 0.77682 |
| Bark beetle - Others | 0.496878 | 0.272385 | 27 | 1.824174 | 0.180882 |

**Fig.6**

**Adult beetles_X. saxesenii**

| Fungi | PDA.Fungus | PDA | p_value | p_adj_Bonferroni | Significance |
| --- | --- | --- | --- | --- | --- |
| R. canadensis | 11 | 2 | 0.00120262 | 0.00721574 | ** |
| R. sulphurea | 10 | 5 | 0.14311098 | 0.85866587 |  |
| R. montetyi | 7 | 5 | 0.68427265 | 1 |  |
| A. hartigii | 5 | 3 | 0.61926962 | 1 |  |
| C. globosum | 5 | 5 | 1 | 1 |  |
| B. bassiana | 3 | 7 | 0.17889541 | 1 |  |

**Larvae_X. saxesenii**

| Fungi | PDA.Fungus | PDA | p_value | p_adj_Bonferroni | Significance |
| --- | --- | --- | --- | --- | --- |
| R. canadensis | 6 | 6 | 1 | 1 |  |
| R. sulphurea | 13 | 0 | 1.923E-07 | 1.1538E-06 | *** |
| R. montetyi | 6 | 3 | 0.34693542 | 1 |  |
| A. hartigii | 2 | 3 | 1 | 1 |  |
| C. globosum | 6 | 4 | 0.6562818 | 1 |  |
| B. bassiana | 5 | 3 | 0.61926962 | 1 |  |

**Adults_X. germanus**

| Fungi | PDA.Fungus | PDA | p_value | p_adj_Bonferroni | Significance |
| --- | --- | --- | --- | --- | --- |
| A. grosmanniae | 15 | 5 | 0.00384753 | 0.01923764 | * |
| A. hartigii | 9 | 7 | 0.7244347 | 1 |  |
| R. sulphurea | 3 | 8 | 0.08610894 | 0.43054469 |  |
| C. globosum | 5 | 4 | 1 | 1 |  |
| B. bassiana | 5 | 6 | 1 | 1 |  |

**Larvae_X. germanus**

| Fungi | PDA.Fungus | PDA | p_value | p_adj_Bonferroni | Significance |
| --- | --- | --- | --- | --- | --- |
| A.grosmanniae | 11 | 1 | 0.00010724 | 0.00053621 | *** |
| A.hartigii | 13 | 0 | 1.923E-07 | 9.6148E-07 | *** |
| R. sulphurea | 4 | 2 | 0.56709957 | 1 |  |
| C. globosum | 6 | 1 | 0.02913753 | 0.14568765 |  |
| B. bassiana | 1 | 0 | 1 | 1 |  |

**Supplementary Figures**

**Figure S4**

Global PERMANOVA

| Transformation | NMDS_Stress | PERMANOVA_F | PERMANOVA_R2 | PERMANOVA_p | Betadisp_F | Betadisp_p |
| --- | --- | --- | --- | --- | --- | --- |
| log1p | 0.088176809 | 3.984512 | 0.227888 | 0.007 | 0.939171 | 0.392 |

Pairwise PERMANOVAs

| Group1 | Group2 | PERMANOVA_F | PERMANOVA_R2 | PERMANOVA_p_raw | Betadisp_F | Betadisp_p | PERMANOVA_p_adj |
| --- | --- | --- | --- | --- | --- | --- | --- |
| Ambrosia | Bark | 4.544239 | 0.193009 | 0.004 | 1.014757 | 0.309 | 0.012 |
| Ambrosia | others | 1.294737 | 0.070771 | 0.248 | 0.119635 | 0.74 | 0.248 |
| Bark | others | 6.538539 | 0.26646 | 0.008 | 1.719799 | 0.206 | 0.012 |

**Figure S5**

**Total amino acids**

Modell

Normal_log

| Resid. Df | Resid. Dev | Df | Deviance | Pr(>Chi) |
| --- | --- | --- | --- | --- |
| 29 | 7.82454 |  |  |  |
| 27 | 5.09405 | 2 | 2.73049 | 0.00072 |

| contrast | estimate | SE | df | t.ratio | p.value | Signif |
| --- | --- | --- | --- | --- | --- | --- |
| Ambrosia beetle - Bark beetle | -0.53806 | 0.189785 | 27 | -2.83509 | 0.022591 | * |
| Ambrosia beetle - Others | 0.157139 | 0.199575 | 27 | 0.787371 | 0.713845 |  |
| Bark beetle - Others | 0.695197 | 0.19523 | 27 | 3.56091 | 0.003864 | ** |

**Woody amino acids**

Modell

Normal_log

| Resid. Df | Resid. Dev | Df | Deviance | Pr(>Chi) |
| --- | --- | --- | --- | --- |
| 29 | 11.34271 |  |  |  |
| 27 | 6.611814 | 2 | 4.730894 | 6.38E-05 |

| contrast | estimate | SE | df | t.ratio | p.value | Signif |
| --- | --- | --- | --- | --- | --- | --- |
| Ambrosia beetle - Bark beetle | -0.85412 | 0.216218 | 27 | -3.95029 | 0.00142 | ** |
| Ambrosia beetle - Others | -0.06743 | 0.22737 | 27 | -0.29657 | 0.952758 |  |
| Bark beetle - Others | 0.786692 | 0.222421 | 27 | 3.536952 | 0.004105 | ** |

**Suppl. Fig. S6**

**Volatiles**

| Metabolite | Group | Test | Statistic | P_value | Significance |
| --- | --- | --- | --- | --- | --- |
| Ethanol | Ambrosia beetle fungi | Wilcoxon | 74 | 0.0734632 |  |
| Ethanol | Bark beetle fungi | Wilcoxon | 106 | 0.01455937 | * |
| Ethanol | Others | Wilcoxon | 49 | 0.42168965 |  |
| Ethyl_acetate | Ambrosia beetle fungi | Wilcoxon | 92 | 0.00072528 | *** |
| Ethyl_acetate | Bark beetle fungi | Wilcoxon | 95.5 | 0.07421791 |  |
| Ethyl_acetate | Others | Wilcoxon | 71 | 0.00743216 | ** |
| Isoamyl_alcohol | Ambrosia beetle fungi | Wilcoxon | 63.5 | 0.32556941 |  |
| Isoamyl_alcohol | Bark beetle fungi | Wilcoxon | 104 | 0.02037045 | * |
| Isoamyl_alcohol | Others | Wilcoxon | 48.5 | 0.46719311 |  |

**Total amino acids**

| Group | Test | Statistic | P_value |
| --- | --- | --- | --- |
| Ambrosia beetle fungi | Welch t-test | -1.1476935 | 0.27128278 |
| Bark beetle fungi | Welch t-test | 0.24680005 | 0.80797716 |
| others | Wilcoxon | 25 | 0.19025093 |

**Leucine and Phenylalanine**

| Group | Test | Statistic | P_value |
| --- | --- | --- | --- |
| Amrosia beetle fungi | Wilcoxon | 74 | 0.07566157 |
| Bark beetle fungi | Wilcoxon | 83.5 | 0.13921631 |
| others | Welch t-test | 1.25209919 | 0.22965851 |
