# Supplementary material for: Bark and ambrosia beetle fungi emit a greater diversity of volatile organic compounds than other fungi
Source: FEMS Microbiol Ecol. 2026 Oct 5;102(11):fiag115. doi: 10.1093/femsec/fiag115 (PMC13673984; doi:10.1093/femsec/fiag115)
Supplement: Supplementary file 1 [file fiag115_supplemental_files.zip › Supporting Information.docx]

Overview of supplementary material

**Supplementary Figure 1 till 6:** See main manuscript as well as Figure Legends doc file for individual legends.

**Supplementary Table 1-6:** See main manuscript as well as Figure Legends doc file for individual legends.

**SI_Methods Lehenberger et al:** Supplementary Methods containing details about the DNA extraction and barcoding, the maximum likelihood analyses as well as the statistical analyses of volatiles including references.

**Supplementary Data**

Suppl. Data Fig. 2: Data included in Fig. 2

Suppl. Data Fig. 3: Data included in Fig. 3

Suppl. Data Fig. 4: Data included in Fig. 4

Suppl. Data Fig. 5: Data included in Fig. 5

Suppl. Data Suppl. Fig. S1: Data included in Suppl. Fig. S1

Suppl. Data Suppl. Fig. S2-3: Data included in Suppl. Fig. S2-3

Suppl. Data Suppl. Fig. S4: Data included in Suppl. Fig. S4

Suppl. Data Suppl. Fig. S5: Data included in Suppl. Fig. S5

Suppl. Data Suppl. Fig. S6: Data included in Suppl. Fig. S6
